# Mitochondrial localization of key NAD^+^-dependent dehydrogenases explains the exceptionally high biomass yield on ethanol of the yeast *Cyberlindnera jadinii*

**DOI:** 10.1128/aem.00362-26

**Published:** 2026-07-10

**Authors:** Marieke Warmerdam, Yared O. Pijman, Marc P. Pedersen, Koen Wattel, Marcel A. Vieira-Lara, Jack T. Pronk

**Affiliations:** 1 Department of Biotechnology, Delft University of Technology 2860, Delft, the Netherlands; Chalmers tekniska hogskola AB, Gothenburg, Sweden

**Keywords:** *Hansenula polymorpha*, *Candida utilis*, bioreactors, mitochondria

## Abstract

Ethanol is an interesting renewable feedstock for microbial biotechnology. Compared to other yeasts, *Cyberlindnera jadinii* has an exceptionally high biomass yield on ethanol. Simulations with a stoichiometric metabolic model identified an intramitochondrial rather than cytosolic localization of NAD^+^-dependent alcohol and acetaldehyde dehydrogenases as a possible explanation for this high yield. This hypothesis was tested in a comparative study with *Ogataea parapolymorpha,* which, like *C. jadinii*, contains a proton-pumping Complex I NADH dehydrogenase, but shows a lower biomass yield on ethanol. Experiments with an *aox* null mutant showed that the lower biomass yield of *O. parapolymorpha* was not caused by *in vivo* alcohol oxidase activity. Proteome analyses showed that the most abundant ADH isozyme in *C. jadinii* and *O. parapolymorpha* contained and lacked a predicted mitochondrial targeting sequence, respectively. The ADH protein with the highest prevalence in ethanol-limited cultures of *C. jadinii* was predicted to be mitochondrial and accounted for almost 4% of the detected proteome. In subcellular fractionation studies with cells grown under ethanol limitation, ADH showed 10-fold lower specific activity in the mitochondria-enriched fraction of cell homogenates of *O. parapolymorpha* than in the cytosolic fraction. Conversely, *C. jadinii* showed a 50% higher ADH activity in the mitochondria-enriched fraction. A low overall recovery of ADH in mitochondria-enriched fractions of *C. jadinii* was attributed to disruption of the reticulate mitochondrial structures observed in ethanol-grown cells during preparation of cell homogenates. This study demonstrates how subcellular localization of an NAD^+^-dependent dehydrogenase can influence respiratory energy coupling in yeasts that harbor a Complex I NADH dehydrogenase.

**IMPORTANCE:**

This study provides theoretical and experimental evidence that intracellular localization of NAD^+^-dependent dehydrogenases can be a key factor for respiratory energy coupling in yeasts. Based on this, alteration of the cellular localization of NAD^+^-dehydrogenases is identified as an interesting metabolic engineering strategy for improving respiratory energy coupling in eukaryotic cell factories.

## INTRODUCTION

Ethanol is emerging as a promising carbon source for aerobic processes in industrial biotechnology (1, 2). Interest in ethanol is motivated by a need to ultimately replace current plant-based, partially refined carbohydrate feedstocks which, while renewable, are associated with greenhouse gas emissions related to agriculture and processing (3). Low-emission processes for ethanol production by syngas fermentation (4) and fermentation of lignocellulosic agricultural residues (5, 6) already operate at industrial scale. In parallel, ethanol production by (bio)electrochemical reduction of CO_2_ is being intensively investigated (7–10). In addition to providing the prospect for reduction of greenhouse gas emissions, feeding of fed-batch and continuous cultures with concentrated, self-sterilizing ethanol solutions aids process intensification, reduces sterilization costs, and circumvents caramelization issues during heat sterilization of carbohydrate feedstocks (11, 12).

**Fig 1:**
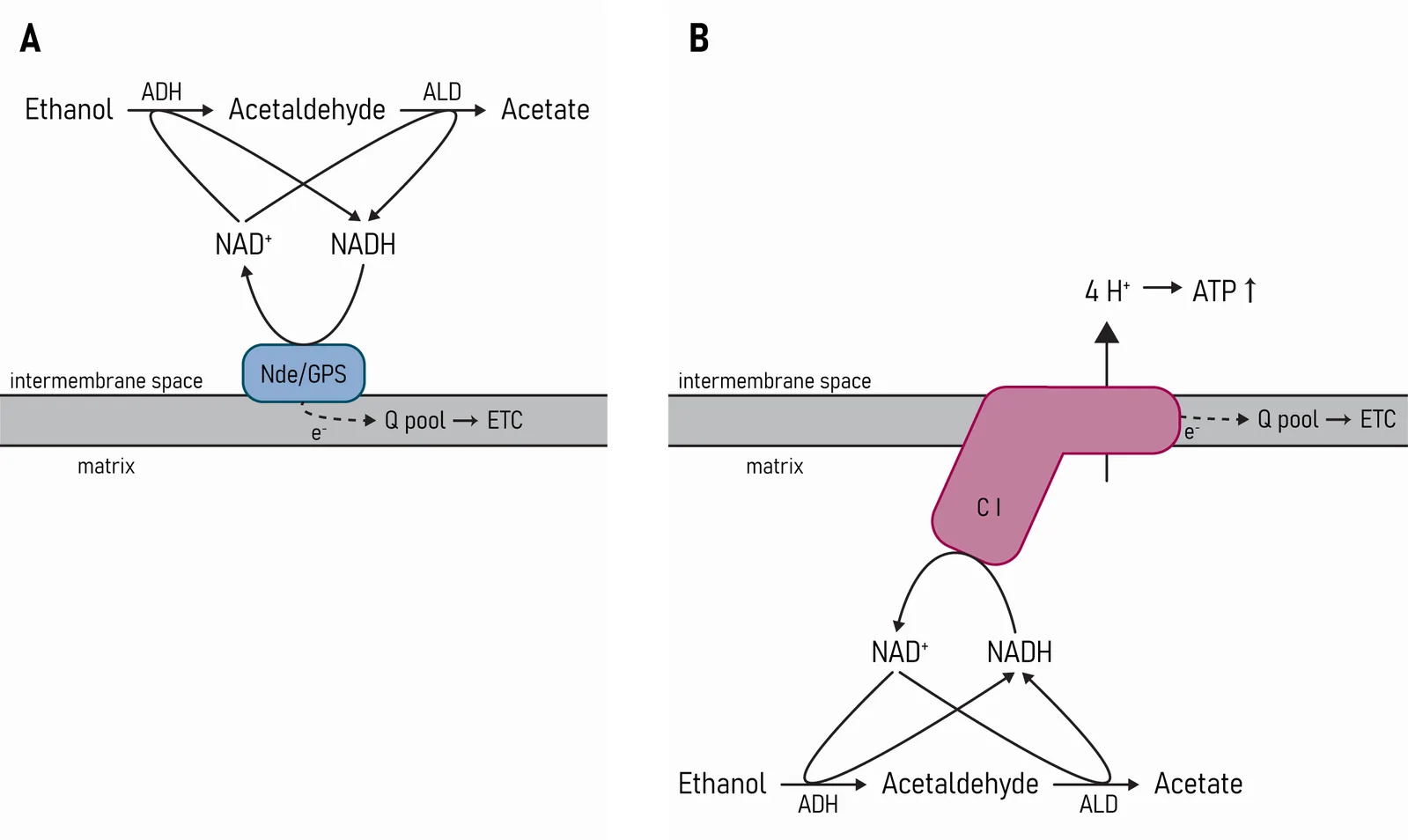
Potential impact of the subcellular localization of alcohol and aldehyde dehydrogenases (ADH and ALD, respectively) on respiratory energy coupling in ethanol-grown cultures of yeasts. (**A**) Oxidation of ethanol and acetaldehyde by cytosolic NAD^+^-dependent dehydrogenases only allows for NADH reoxidation via external non-proton-pumping type-2 NADH dehydrogenase (Nde) or a non-proton-pumping glycerol-3-phosphate shuttle (GPS). These systems donate electrons from NADH to the electron transport chain (ETC) via the ubiquinone:ubiquinol (Q) pool, where further electron transfer through respiratory chain complexes III and IV leads to the translocation of six protons per electron pair (not shown in figure). In the absence of a proton-pumping Complex I as displayed in panel B, intramitochondrial NADH is reoxidized via an internal alternative NADH dehydrogenase (Ndi) which donates the electrons from NADH to the Q pool without additional proton translocation (not shown in figure). (**B**) In yeasts that harbor a multi-subunit Complex I (NADH:ubiquinone oxidoreductase, CI), intramitochondrial localization of NAD^+^-dependent ADH and ALD enables translocation of four additional protons. This additional proton translocation is supposed to allow for the synthesis of more ATP and, thereby, to higher biomass yields in ethanol-grown cultures than in scenario A.

Most industrially relevant yeast species readily use ethanol as sole carbon and energy substrate (13, 14). The reactions that feed ethanol into yeast central carbon metabolism are strongly conserved. After ethanol uptake via passive diffusion (15), NAD^+^-dependent alcohol dehydrogenase (ADH) and NAD(P)^+^-dependent aldehyde dehydrogenase (NAD(P)^+^-ALD) oxidize ethanol to acetate (Fig. 1). Acetyl-CoA synthase (ACS) then activates acetate to acetyl-CoA, which is fed into central carbon metabolism (16). The ACS reaction involves hydrolysis of adenosine triphosphate (ATP) to adenosine monophosphate (AMP) and pyrophosphate (PP_i_). Due to the constitutive expression of adenylate kinase and pyrophosphatase, the *in vivo* energy requirement of this reaction is equivalent to hydrolysis of two molecules of ATP to ADP and inorganic phosphate (17). Despite the apparent uniformity of the entry of ethanol into central carbon metabolism, biomass yields on ethanol differ among yeast species (13, 18, 19). These differences have been attributed to diversity of mitochondrial respiratory energy coupling (13).

Because the yeast inner mitochondrial membrane is impermeable to NADH and NAD^+^, respiratory reoxidation of NADH is strictly compartmentalized (20). In the model yeast *Saccharomyces cerevisiae*, oxidation of NADH generated in the mitochondrial matrix is coupled to respiration by a simple, non-proton-translocating NADH dehydrogenase (Ndi) (21). NADH generated in the yeast cytosol is predominantly re-oxidized by similar NADH dehydrogenases (Nde1, Nde2), whose catalytic sites face the mitochondrial intermembrane space (Fig. 1A) (22), or via the glycerol-3-phosphate shuttle (23). Ndi1, Nde1, Nde2, and the glycerol-3-phosphate shuttle all donate electrons to the ubiquinone:ubiquinol (Q) pool (20). This entry point limits the maximum number of protons translocated per electron pair transferred to oxygen (H^+^/O ratio) to six. Yeasts with this respiratory-chain configuration, such as *S. cerevisiae* and *Kluyveromyces lactis*, exhibit biomass yields on ethanol of approximately 0.6 g biomass∙(g ethanol)^−1^ (13, 18).

Multiple non-*Saccharomyces* yeast species show higher biomass yields on ethanol than *S. cerevisiae* (13). Such yeasts invariably harbor the genes encoding at least 35 subunits of a Complex I NADH dehydrogenase (“Complex I”) (24, 25). Complex I couples the oxidation of intramitochondrial NADH, with ubiquinone as the electron acceptor, to the translocation of four protons (Fig. 1B). Involvement of Complex I thereby increases the maximum H^+^/O ratio for oxidation of intramitochondrial NADH to 10. Whether this ratio is achieved *in vivo* depends on regulation of the expression of Complex I genes and on competition for NADH with Ndi1 orthologs (26).

In a recent study, we compared growth energetics of five ascomycete yeast species whose genomes harbor Complex I genes in ethanol-limited chemostat cultures. In that study, *Cyberlindnera jadinii* (formerly *Candida utilis*) exhibited a significantly higher biomass yield on ethanol than the other four species [at least 9% higher; 0.73 g biomass∙(g ethanol)^−1^] (13). This result was consistent with earlier reports on high biomass yields on ethanol of *C. jadinii* and the related species *C. saturnus* (18, 19). Analysis of the growth energetics of multiple *C. jadinii* strains showed that, in fast-growing cultures, their biomass yields on ethanol approached the theoretical maximum yield imposed by the stoichiometry of assimilatory ethanol metabolism (12). This exceptionally high biomass yield was reached even though biomass in these cultures showed a high content (0.54 g·g^−1^) of protein, which, in terms of ATP costs, is the most expensive macromolecule in microbial biomass (27). Under identical conditions, the industrial yeast *Ogataea parapolymorpha* (formerly *Hansenula polymorpha*), which also harbors a functional Complex I, showed a biomass yield of only 0.67 g biomass∙(g ethanol)^−1^ (13). This indicates metabolic differences beyond the presence or absence of Complex I that affect energy coupling in ethanol-grown yeast cultures.

*C. jadinii* has a long history in the production of single-cell protein for animal and human nutrition (28, 29). Understanding the basis for its efficient ethanol metabolism could contribute to further ethanol-based biotechnological applications of *C. jadinii* and guide metabolic engineering strategies for improving ATP yields in other yeasts and filamentous fungi. The goal of this study is therefore to investigate the basis for the exceptionally high biomass yield of *C. jadinii* on ethanol. Based on simulations with a stoichiometric metabolic network model, we hypothesize a key role for a predominantly mitochondrial localization of NAD^+^-dependent ADH and ALD. This hypothesis was tested by a combination of growth studies in ethanol-limited chemostat cultures of *C. jadinii*, *S. cerevisiae*, and *O. parapolymorpha*; proteome analyses; and cellular fractionation experiments.

## RESULTS

### Analysis of the involvement of alcohol oxidase and Complex I in ethanol dissimilation by *Ogataea parapolymorpha*

In a previous study, *O. parapolymorpha* CBS 11895 showed a 9% lower biomass yield in ethanol-limited chemostat cultures grown at a dilution rate (D) of 0.10 h^−1^ than *C. jadinii* CBS 621 (0.67 versus 0.73 g biomass∙(g ethanol)^−1^) (13). When grown at the same D in aerobic glucose-limited cultures, biomass yields of these two yeasts were similar (0.53 and 0.51 g biomass·(g glucose)^−1^, respectively) (12, 30). *O. parapolymorpha* and *C. jadinii* were therefore selected for a comparative study aimed at identifying key factors involved in energy coupling in ethanol-grown yeast cultures.

A possible complication for studying ethanol metabolism in *O. parapolymorpha* is that, in contrast to *C. jadinii*, this yeast can grow on methanol (31, 32). In methylotrophic yeasts, oxidation of methanol to formaldehyde is catalyzed by a hydrogen-peroxide-generating alcohol oxidase (AOX) that can also use ethanol as a substrate (33). Because the AOX reaction does not produce NADH and consequently cannot be linked directly to oxidative phosphorylation, its *in vivo* involvement in ethanol oxidation could cause a lower biomass yield on ethanol. Simulation with a stoichiometric metabolic network model predicted a 14% lower biomass yield on ethanol when AOX completely replaces alcohol dehydrogenase (ADH) (Supplemental Material 2). The effect of abolishing AOX activity on biomass yield of *O. parapolymorpha* on ethanol was therefore experimentally investigated.

**Fig 2:**
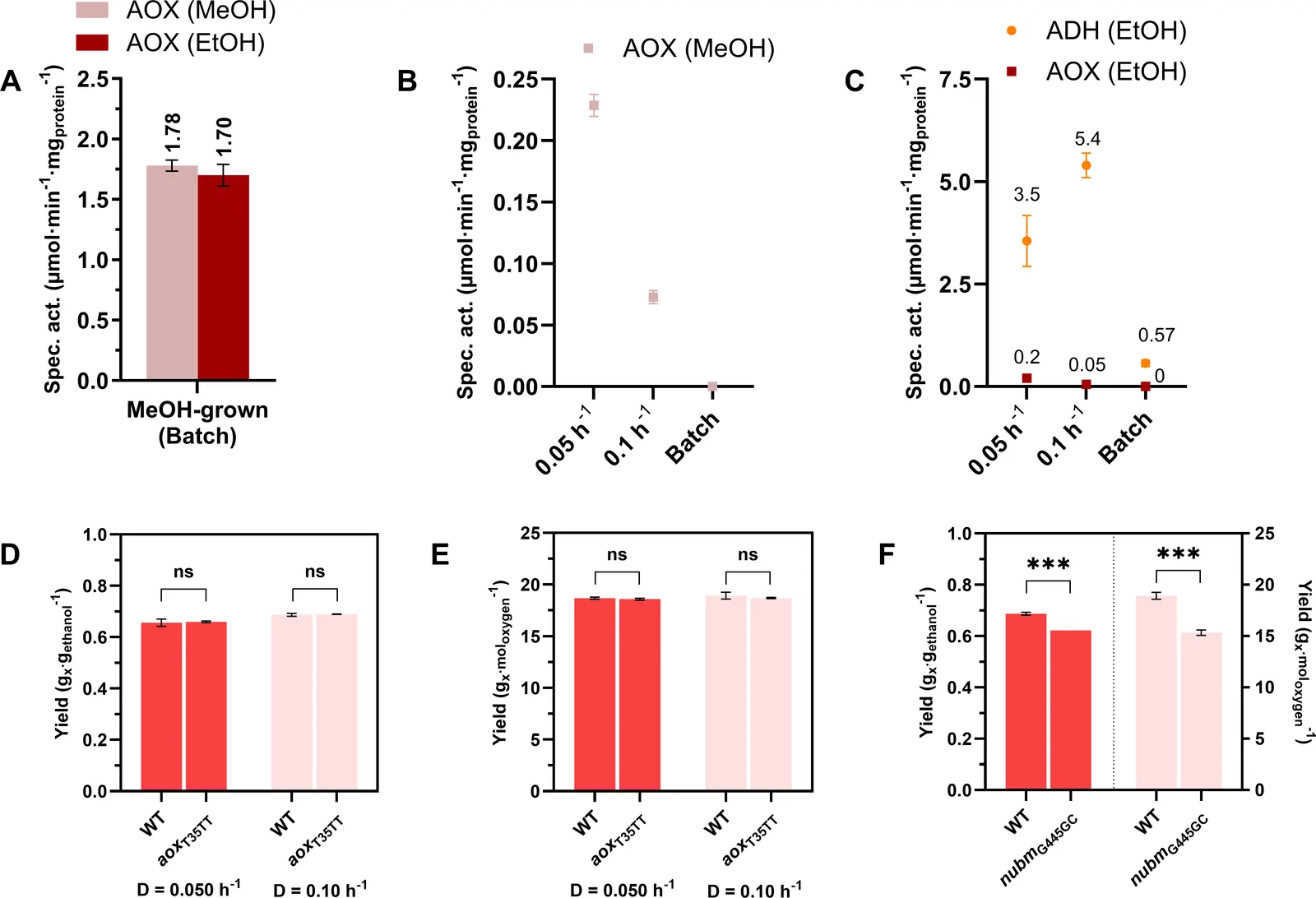
Alcohol oxidase (AOX) activity in cell-free extracts of methanol- and ethanol-grown *O. parapolymorpha* and the impact of the presence of AOX and functional Complex I on biomass yields in ethanol-limited chemostat cultures. Except for panel A, all panels show data on ethanol-grown cultures. (**A**) AOX activity with methanol or ethanol as substrates in cell extracts of methanol-grown batch cultures of strain CBS 11895 (WT). (**B**) AOX activities in cell extracts of ethanol-grown cultures of strain CBS 11895, measured with methanol as a substrate. (**C**) Comparison of AOX and ADH activities, both measured with ethanol as a substrate, in cell extracts of ethanol-grown cultures of strain CBS 11,895. (**D**) Biomass yields on ethanol of *O. parapolymorpha* CBS 11895 and of the congenic *aox* null strain IMD035 in ethanol-limited chemostat cultures. (**E**) Biomass yields on oxygen of strains CBS 11,895 and IMD035 in ethanol-limited chemostat cultures. Aerobic, ethanol-limited chemostats were run at 30°C and pH 5.0, at *D* = 0.050 h^−1^ and *D* = 0.10 h^−1^ (ns = *P* > .05; two-sample *t*-test). (**F**) Biomass yields on ethanol of *O. parapolymorpha* CBS 11895 and the congenic *nubm* null strain IMD010 (nubm encodes an essential Complex I subunit) in aerobic, ethanol-limited chemostat cultures run at 30°C, pH 5.0 and *D* = 0.10 h^−1^ (***P < 0.001, two-sample *t*-test). Data shown in panels **B** and **D–F** are also included in Table 1. Data show mean ± standard deviation, *n* = 2.

Consistent with earlier reports (33), cell extracts of methanol-grown batch cultures of *O. parapolymorpha* CBS 11895 showed similar AOX activities with methanol and ethanol as substrates (Fig. 2A). In line with literature reports that ethanol represses AOX synthesis in *O. parapolymorpha* (34–36), no AOX activity using methanol was observed in cell extracts of ethanol-grown batch cultures (Fig. 2B). In cell extracts of ethanol-limited chemostat cultures, AOX activity was approximately 5% of the activity of ADH when cells were grown at *D* = 0.050 h^−1^ and approximately 1% when cells were grown at *D* = 0.10 h^−1^ (Fig. 2C). These results indicated partial de-repression of AOX synthesis at the low residual ethanol concentrations in ethanol-limited cultures. To investigate whether these low AOX activities affected biomass yields, the congenic *aox* null mutant *O. parapolymorpha* IMD035 was constructed. No AOX activity was detected in cell extracts of ethanol-limited chemostat cultures (*D* = 0.050 h^−1^ and *D* = 0.10 h^−1^) of this strain grown under ethanol limitation (Table 1). In ethanol-limited chemostat cultures, biomass yields on ethanol and oxygen were not significantly different for strains CBS 11895 and IMD035 (Fig. 2D and E, Table 1). These results indicated that AOX does not significantly influence biomass yield in ethanol-limited chemostat cultures of strain CBS 11895.

**TABLE 1:** Physiological parameters of yeast strains grown in steady-state, aerobic, ethanol-limited chemostats at 30°C and pH 5.0, with ethanol concentrations below the HPLC detection limit (3 mg∙L^−1^) in all cultures

	D (h^−1^)	Y_x/s_ (g_x_∙g_ethanol_^−1^)	q_s_ (g_s_∙g_x_^−1^∙h^−1^)	C rec (%)	Y_x/O2_ (g_x_∙mol_O2_^−1^)	RQ (mol_CO2_∙mol_O2_^−1^)	AOX activity (µmol_ethanol_∙mg_protein_^−1^∙min^−1^)
*S. cerevisiae* CEN.PK113-7D (*n* = 4)	0.102 ± 0.002	0.604 ± 0.006	0.169 ± 0.003	100 ± 2	15.1 ± 0.4	0.52 ± 0.02	n.d.^*a*^
*C. jadinii* CBS621 (*n* = 6)	0.100 ± 0.001	0.745 ± 0.007	0.134 ± 0.001	103 ± 2	22.2 ± 0.4	0.48 ± 0.03	n.d.^*a*^
*O. parapolymorpha* CBS 11895 (*n* = 5)	0.099 ± 0.002	0.687 ± 0.006	0.144 ± 0.003	101 ± 2	18.9 ± 0.3	0.49 ± 0.02	0.05 ± 0.01^*b*^
*O. parapolymorpha* IMD010 (*nubm_G445GC_*, *n* = 2)	0.101 ± 0.001	0.622 ± 0.001	0.162 ± 0.001	101 ± 1	15.3 ± 0.3	0.50 ± 0.01	n.d.^*a*^
*O. parapolymorpha* IMD035 (*aox_T35TT_*, *n* = 2)	0.099 ± 0.001	0.690 ± 0.001	0.143 ± 0.001	103 ± 1	18.7 ± 0.1	0.51 ± 0.01	<0.004
*O. parapolymorpha* CBS 11895 (*n* = 2)	0.049 ± 0.001	0.66 ± 0.01	0.0746 ± 0.0008	105 ± 2	18.7 ± 0.1	0.59 ± 0.01	0.20 ± 0.03^*b*^
*O. parapolymorpha* IMD035 (*aox_T35TT_*, *n* = 2)	0.050 ± 0.001	0.660 ± 0.004	0.0751 ± 0.0002	106 ± 1	18.6 ± 0.1	0.59 ± 0.01	<0.004

^*a*^ n.d., not determined.

^*b*^ *n *= 2.

To investigate the involvement of Complex I in ethanol dissimilation in ethanol-limited chemostat cultures of *O. parapolymorpha* CBS 11895, its biomass yield on ethanol was compared with that of the congenic strain IMD010. In this strain, inactivation of the *nubm* gene abolishes Complex I activity (30). In ethanol-limited chemostat cultures grown at *D* = 0.10 h^−1^, the *nubm* mutant strain showed a significantly lower biomass yield on both ethanol and oxygen than the wild type (Fig. 2F, Table 1). The result confirmed that a lack of NADH reoxidation via Complex I negatively affects biomass yields of ethanol-limited cultures of *O. parapolymorpha*.

### Model-based prediction of the impact of cellular location of key dehydrogenases on biomass yield in ethanol-grown cultures

The proton-pumping Complex I in yeasts only accepts electrons from NADH located in the mitochondrial matrix (Fig. 1) (20). Thus, a mitochondrial localization of NADH-generating dehydrogenases involved in respiratory ethanol dissimilation has a clear bioenergetic advantage over a cytosolic localization. We therefore hypothesize that differences in cellular localization of alcohol and/or acetaldehyde dehydrogenases (ADH and ALD, respectively) may be responsible for the different biomass yields of Complex I-containing yeast species in ethanol-grown cultures (13). To investigate this hypothesis, biomass yields and biomass composition were measured in three yeast species: (i) *S. cerevisiae*, whose genome lacks genes encoding the subunits of Complex I (37, 38); (ii) *C. jadinii*, whose genome does encode Complex I subunits and which shows exceptionally high biomass yields on ethanol (12); and (iii) *O. parapolymorpha*. The latter is a genetically tractable yeast that harbors a functional Complex I and for which high-quality genome sequence information is available (30, 39, 40) but shows a 9% lower biomass yield on ethanol than *C. jadinii* (13).

A compartmented stoichiometric metabolic network model, originally developed for *S. cerevisiae* (13, 41), was adapted to compare and assess the theoretical impact on biomass yield of the subcellular localization of ADH and ALD in *S. cerevisiae*, *C. jadinii*, and *O. parapolymorpha* (Table A1, Supplemental Material 2). The reaction catalyzed by Complex I was added to the model to simulate respiratory energy coupling in *C. jadinii* and *O. parapolymorpha*. Published data on macromolecular composition of biomass (protein, carbohydrate, RNA, and lipid content) for these species in ethanol-grown chemostat cultures (Table A2) (13) were implemented in the model to account for ATP costs of the synthesis of protein, carbohydrates, nucleic acids, and lipids. Metabolic pathways involved in the synthesis of these macromolecules were assumed to be conserved in the three yeast species (42).

**Fig 3:**
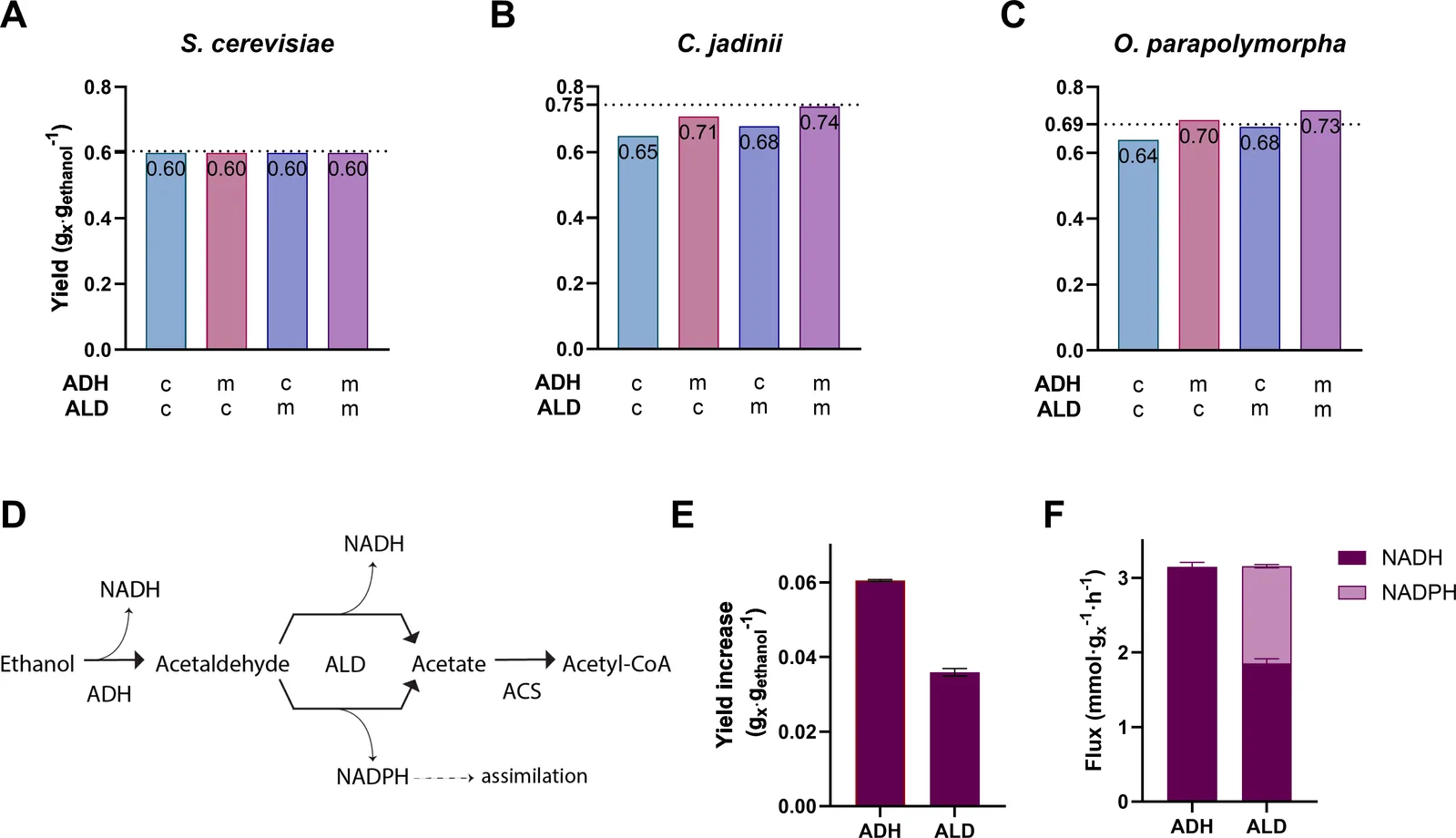
Model predictions of the impact of the cellular localization of NAD^+^-dependent alcohol dehydrogenase (ADH) and NAD^+^-dependent acetaldehyde dehydrogenase (ALD) on biomass yields on ethanol of *S. cerevisiae*, *C. jadinii,* and *O. parapolymorpha*. Biomass yields were calculated with a compartmented metabolic network model based on measured macromolecular biomass compositions in aerobic, ethanol-limited chemostat cultures (*D* = 0.10 h^−1^) (13) and the presence/absence of a proton-pumping Complex I NADH dehydrogenase complex. The simulated localization of ADH and ALD is indicated by c (cytosolic) or m (mitochondrial). Dotted lines indicate experimentally observed biomass yields. (**A**) *S. cerevisiae* (experimental biomass yield 0.604 ± 0.006 g∙g^−1^, *n* = 4, Table 1). (**B**) *C. jadinii* (experimental biomass yield 0.745 ± 0.007 g∙g^−1^, *n* = 6, Table 1). (**C**) *O. parapolymorpha* (experimental biomass yield 0.687 ± 0.006 g∙g^−1^, *n* = 5, Table 1). (**D**) Assumption in model: NADPH for use in assimilation is preferentially generated by NADP^+^-ALD. (**E**) Predicted impact on biomass yield on ethanol, averaged for *C. jadinii* and *O. parapolymorpha* (panels **B** and **C**, respectively) upon simulated mitochondrial targeting of either ADH or NAD^+^-dependent ALD. (**F**) Model-based simulation of *in vivo* cofactor use by ADH and ALD, averaged over the 12 simulated scenarios shown in panels **A–C** (mean ± SEM). Model parameters, solutions, and code are provided in Supplemental Material 2 through 4.

Due to the absence of Complex I in *S. cerevisiae*, the localization of ADH and NAD^+^-dependent ALD to either the cytosol or mitochondrial matrix did not influence model predictions for biomass yields on ethanol of this yeast (Fig. 3A). Regardless of the subcellular localization of ADH and ALD, the presence of Complex I in *C. jadinii* and *O. parapolymorpha* led to a higher predicted biomass yield on ethanol than calculated for *S. cerevisiae* (Fig. 3B and C). A higher predicted yield was anticipated because three of the six electron pairs released during ethanol dissimilation are released as NADH in mitochondrial TCA-cycle reactions. In *C. jadinii* and *O. parapolymorpha*, this enables translocation of additional protons by Complex I (Fig. 1; Supplemental Material 2).

Individually localizing ADH or ALD to the mitochondrial matrix led to higher simulated biomass yields on ethanol (8–9% and 5%, respectively) for both *C. jadinii* and *O. parapolymorpha*, relative to a scenario in which ADH and ALD were both cytosolic. Simulation of a mitochondrial localization of both ADH and ALD even led to a 12–14% higher biomass yield on ethanol for both yeasts (Fig. 3B and C), which corresponded well with the experimentally observed biomass yield of *C. jadinii* (Fig. 3B). A 70% higher impact was predicted of the cellular localization of ADH than of the localization of ALD (Fig. 3E). This outcome was linked to the assumption in the model that NADP^+^-dependent ALD is a preferred source of NADPH for biosynthetic reactions and that NADPH is not re-oxidized by mitochondrial respiration (Fig. 3D and E). As a result, in the 12 simulated scenarios, 38 ± 5% of the ALD activity was NADP^+^-dependent (Fig. 3F; Supplemental Material 2).

A comparison with experimental data (Fig. 3B, Table 1) indicated that the observed biomass yield of *C. jadinii* is consistent with the hypothesis that *in vivo* oxidation of ethanol to acetate via NAD^+^-dependent ADH and ALD predominantly occurs in the mitochondrial matrix. The lower experimental biomass yield of *O. parapolymorpha* (Fig. 3C, Table 1) suggested that a significant fraction of the NADH was generated in the cytosol of this yeast instead of the mitochondrial matrix.

### Genome- and proteome-based assessment of the localization of ADH and ALD isozymes in *O. parapolymorpha* and *C. jadinii*

To assess whether different biomass yields on ethanol of *C. jadinii* and *O. parapolymorpha* indeed correlate with a different localization of ADH and/or ALD, topologies of these enzymes were investigated by genome and proteome analyses.

The *S. cerevisiae* genome harbors seven ADH-encoding genes, of which Adh1-5 are NAD^+^-dependent, while Adh6 and Adh7 are NADP^+^-dependent enzymes with very low activities towards ethanol (43–45). *ADH2* is the highest expressed ADH gene in ethanol-grown cultures of *S. cerevisiae* (41) and encodes a cytosolic ADH. Adh3 is located in the mitochondrial matrix, where it plays a role in anaerobic balancing of the NAD^+^/NADH ratio (46). Adh4, which is not homologous to the other ADH genes of *S. cerevisiae*, was also reported to be mitochondrial (45, 47). The *S. cerevisiae* ALD isoenzymes Ald2 and Ald3 are cytosolic and NAD^+^-dependent, while the cytosolic Ald6 is NADP^+^-dependent (48). Of the mitochondrially located Ald4 and Ald5, Ald4 uses both NAD^+^ and NADP^+^ as cofactor, while Ald5 is NADP^+^-dependent (48).

To investigate the localization of ADHs in *O. parapolymorpha* and *C. jadinii*, published genome sequences of sufficient genome assembly quality to quantify the number of orthologous genes (40, 49) were searched for orthologs of *S. cerevisiae ADH2*, *ADH3*, and *ADH6*. The presence of an N-terminal mitochondrial targeting sequence (MTS) was assessed with the algorithms TargetP (50) and DeepMito (51). These algorithms provide a statistical likelihood that a protein is targeted to the mitochondria *in vivo*, based on enrichment of positively charged amino acid residues and a propensity to form an N-terminal amphiphilic helical structure (50, 52, 53). Of six putative ADH genes identified in *O. parapolymorpha*, only one was predicted to encode a mitochondrial ADH (Table A3). In contrast, of seven predicted ADH orthologs in *C. jadinii* (Table A4), four were predicted to be mitochondrial. A search for orthologs of *S. cerevisiae ALD3*, *ALD4*, and *ALD6*, which represent the three types of acetaldehyde dehydrogenase genes in *S. cerevisiae* (48), yielded eight hits in *O. parapolymorpha*, of which two were predicted to be mitochondrial (Table A5). Of 10 hits found in *C. jadinii*, three were predicted to be mitochondrial (Table A6).

To investigate expression levels of the putative cytosolic and mitochondrial ADH and ALD isoenzymes in ethanol-limited cultures of *O. parapolymorpha* and *C. jadinii*, a mass-spectrometry-based proteome analysis was performed on duplicate steady-state aerobic, ethanol-limited chemostat cultures (*D* = 0.10 h^−1^) of each yeast. Using a label-free method based on the top three peptides measured for each protein, similar to the procedure used in (12), 1,731 proteins were detected and quantified for *O. parapolymorpha* and 1,633 for *C. jadinii* (Supplemental Material 5).

**Fig 4:**
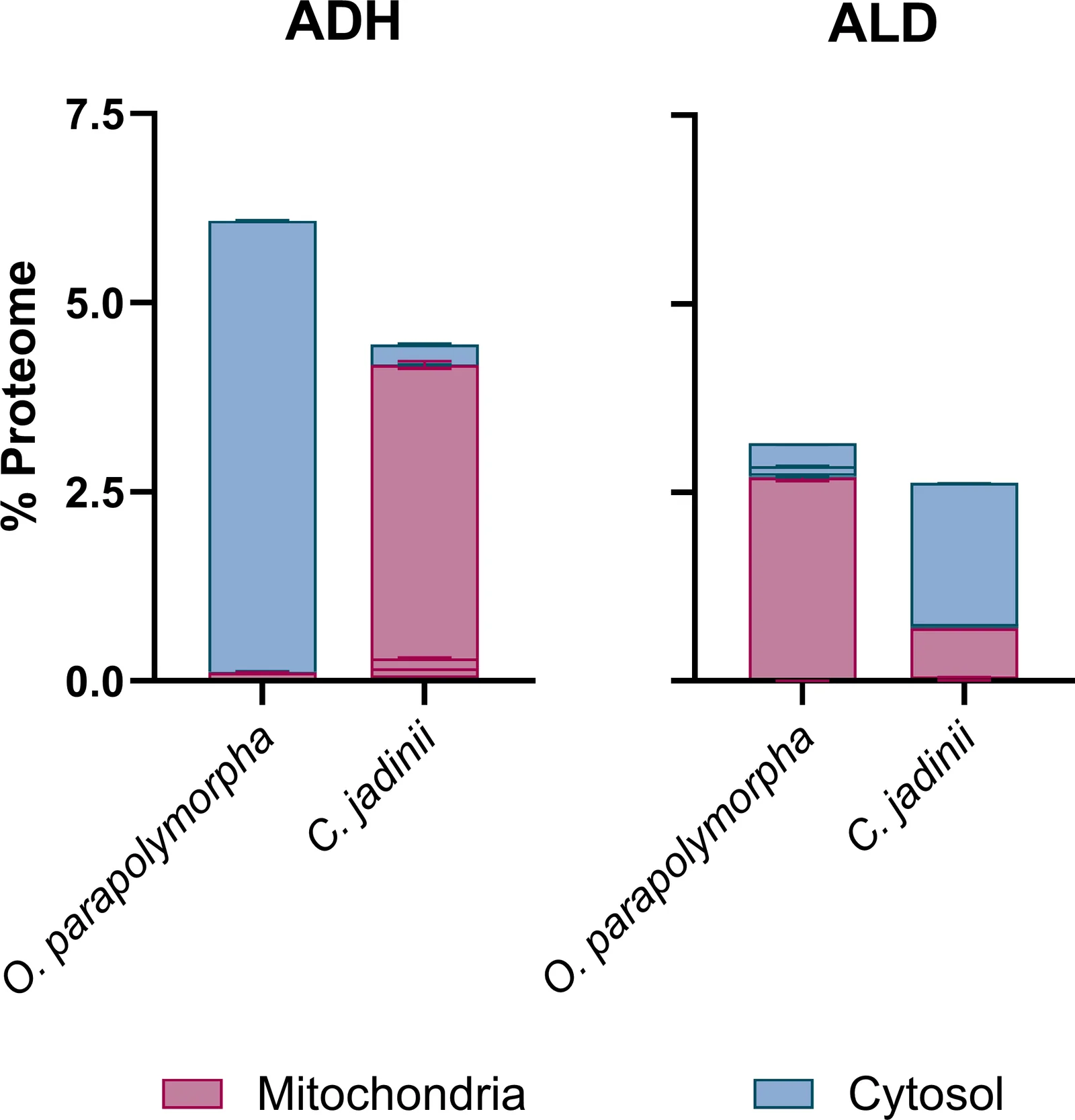
Abundance and predicted cellular localization of putative alcohol dehydrogenase (ADH) and acetaldehyde dehydrogenase (ALD) proteins detected in *O. parapolymorpha* and *C. jadinii* grown in aerobic, ethanol-limited chemostats (*D* = 0.10 h^−1^). Stacked bars represent the average and standard deviation of the abundance of each ADH (left) and ALD (right) protein in the detected proteome of duplicate steady-state, ethanol-limited bioreactors (Tables A7–A10, Supplemental Material 5). Cytosolic or mitochondrial localization of proteins was derived as a consensus from predictions by the MitoProt (54), TargetP (50), and DeepMito (51) algorithms.

In *O. parapolymorpha* as well as in *C. jadinii*, ADH isoenzymes accounted for a considerable fraction of the detected proteome, which is in agreement with a previous proteome analysis on ethanol-limited chemostat cultures of *C. jadinii* (12). However, a striking difference between the two yeasts was observed concerning the occurrence of a predicted MTS in the detected proteins (Fig. 4). Of 3 ADH proteins detected in ethanol-limited cultures of *O. parapolymorpha* (Table A7, Supplemental Material 5), the single protein that was predicted to be mitochondrial accounted for only 1.8 ± 0.1% of the total ADH protein (Fig. 4; Table A7). In contrast, of seven ADH isozymes detected in the proteome of the *C. jadinii* chemostat cultures, five were predicted to contain an MTS. Together, these putative mitochondrial ADHs accounted for 94.0 ± 0.2 % of the ADH protein in these cultures (Fig. 4; Table A8). One of the predicted mitochondrial ADHs in *C. jadinii* (UniProt ID A0A0H5C8Q7) represented 3.87 ± 0.05 % of the entire proteome. Conversely, the most abundant *O. parapolymorpha* ADH (UniProt ID W1QFP1), which accounted for 5.97 ± 0.01 % of the proteome, was predicted to be cytosolic.

In the proteome data of both *O. parapolymorpha* and *C. jadinii*, seven putative ALD proteins were detected, of which two and three, respectively, had a high likelihood of being mitochondrially localized (Fig. 4). The most abundant ALD in *O. parapolymorpha* had a predicted MTS (Table A9), while the most abundant ALD in *C. jadinii* did not (Table A10). The cofactor preference of ALD enzymes could not be determined based on amino acid sequence data.

At least 20 different Complex I subunits were detected in the proteome of *O. parapolymorpha* (Table A11) and *C. jadinii* (Table A12), which confirmed the presence of a proton-pumping NADH:ubiquinone oxidoreductase complex in ethanol-grown cultures of both yeasts. Additionally, peptides representing three alternative, non-proton-pumping NADH dehydrogenases were detected in the proteomes of both yeasts (Table A11 and A12, Supplemental Material 5). Orientation of these alternative NADH dehydrogenases in the mitochondrial inner membrane cannot be established based on amino acid sequence data alone. Therefore, we cannot unequivocally assign an orientation of *C. jadinii* Ndi/Nde orthologs. Orientation of these proteins in *O. parapolymorpha* CBS 11895 was recently elucidated (55). By combining those results with our proteome data set, one internal and two external alternative NADH dehydrogenases were detected in ethanol-grown *O. parapolymorpha* (Supplemental Material 5).

### Subcellular fractionation studies for investigating cellular location of ADH and ALD in *C. jadinii* and *O. parapolymorpha*

**Fig 5:**
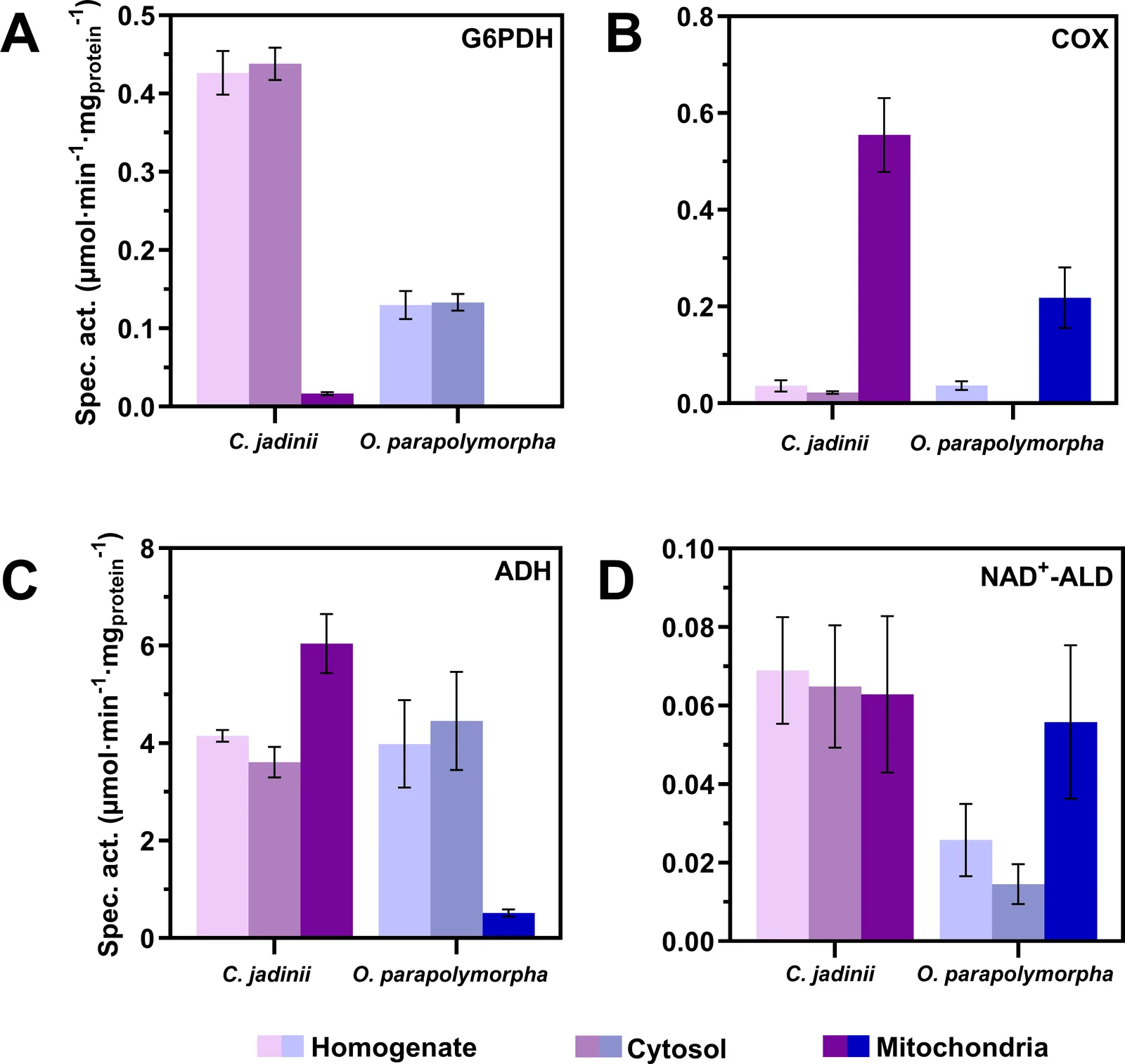
Specific activities of selected enzymes in homogenate, soluble, and particulate fractions of *C. jadinii* and *O. parapolymorpha* cells grown in aerobic, ethanol-limited chemostat cultures (*D* = 0.10 h^−1^). Specific activities are shown as the mean of duplicate assays with error bars representing standard deviation. (**A**) Glucose-6-phosphate dehydrogenase (G6PDH). (**B**) Cytochrome *c* oxidase (COX). (**C**) NAD^+^-dependent alcohol dehydrogenase (ADH). (**D**) NAD^+^-dependent aldehyde dehydrogenase (NAD^+^-ALD).

To further investigate the localization of ADH and ALD, a subcellular fractionation method developed for *C. jadinii* (56) and *S. cerevisiae* (22) that separates mitochondria-enriched particulate and soluble (i.e., cytosolic) fractions of cell homogenates was applied to duplicate ethanol-limited chemostat cultures (*D* = 0.10 h^−1^) of *C. jadinii* and *O. parapolymorpha*. For both yeasts, the specific activity of the cytosolic marker enzyme glucose-6-phosphate dehydrogenase was over 10-fold lower in the particulate fraction than in the homogenate and soluble fractions (Fig. 5A). In addition, the mitochondrial membrane-associated enzyme cytochrome *c* oxidase was strongly enriched in the particulate fraction (Fig. 5B) of both yeasts, indicating mitochondria enrichment.

An approximately 10-fold lower specific activity of ADH was found in the mitochondria-enriched fraction of *O. parapolymorpha* than in the complete homogenate and soluble fraction (Fig. 5C). This result was consistent with the predominantly cytosolic localization predicted by the proteome analyses. In contrast, the specific activity of ADH in the particulate fraction of *C. jadinii* homogenates was approximately 50% higher than in the homogenate and soluble fraction (Fig. 5C). However, only 15 ± 2% of the total activity of ADH present in the homogenate was recovered in the particulate fraction (Table A13) compared to 1.3 ± 0.7% in *O. parapolymorpha* (Table A14). In fractionation experiments with *O. parapolymorpha*, the specific activity of NAD^+^-dependent ALD was 3- to 4-fold higher in the particulate fraction than in homogenate and soluble fractions (Fig. 5D). In *C. jadinii*, specific activities of ALD in these three fractions were not significantly different. Recoveries of NAD^+^-dependent ALD in the mitochondrial fractions of *C. jadinii* and *O. parapolymorpha* were 16 ± 4% and 20 ± 9%, respectively.

Activities of ADH, NAD^+^- and NADP^+^-dependent ALD, and glucose-6-phosphate dehydrogenase were measured in cell extracts prepared by sonication of chemostat-grown cells. Activities measured in these cell extracts corresponded well with those in homogenates prepared by enzymic and mechanical disruption (Fig. A1 and Table A15). ADH activities in cell extracts of ethanol-limited chemostat cultures of *C. jadinii* and *O. parapolymorpha* were 10-fold higher than in cell extracts of fast-growing batch cultures. This result agreed with a previous report that showed a high abundance of ADH proteins in ethanol-limited cultures (Fig. 4) (12). In chemostat cultures of *C. jadinii* and *O. parapolymorpha*, activities of ALD measured with NADP^+^ as a cofactor were 5-fold and 8-fold higher, respectively, than ALD activities measured with NAD^+^ (Table A15).

**Fig 6:**
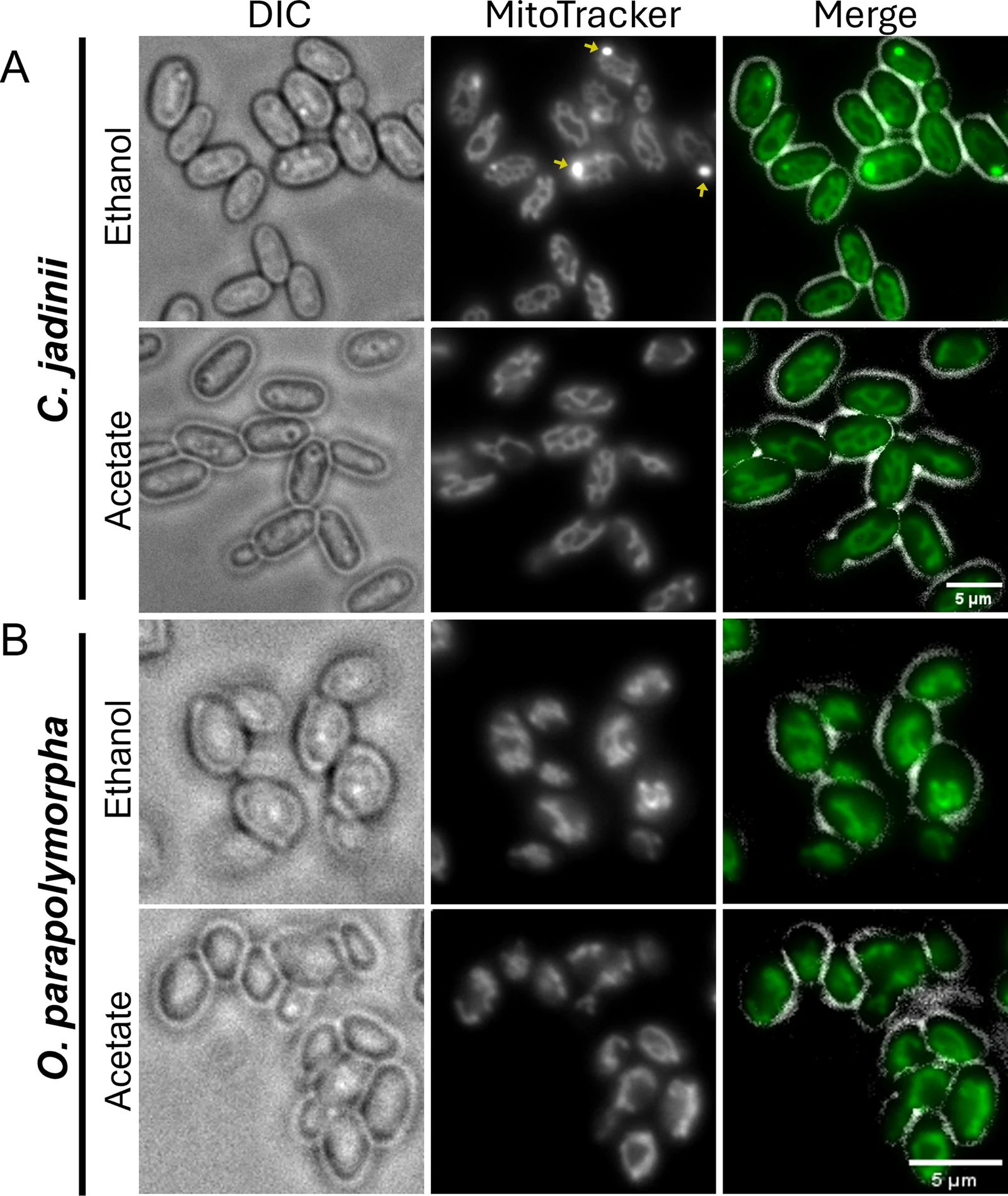
Mitochondrial morphology in ethanol- and acetate-grown *C. jadinii* and *O. parapolymorpha*. (**A**) Fluorescence microscopy images of *C. jadinii* cells grown in ethanol- and acetate-limited chemostat cultures (*D* = 0.10 h^−1^). Yellow arrows indicate fluorescent spots. (**B**) Fluorescence microscopy images of *O. parapolymorpha* cells grown in ethanol- and acetate-limited chemostat cultures (*D* = 0.10 h^−1^). DIC, differential interference contrast. The displayed images are representative samples of duplicate reactors. Mitochondria were visualized with MitoTracker and without as control (Fig. A2). Scale bars indicate 5 µm.

Subcellular fractionation using a combination of enzymatic degradation of the yeast cell wall, reduction of the osmotic pressure, and mechanical cell disruption is known to lead to the release of mitochondrial matrix proteins into the soluble fraction (56, 57). The extent of lysis is often assessed by measuring activities of marker enzymes for the mitochondrial matrix. However, we were unable to identify such proteins for the two non-conventional yeasts used in this study. Since breakage of mitochondria may be related to their shape and size, mitochondrial morphology was studied in ethanol-limited chemostat cultures of the two yeasts (Fig. 6; Fig. A2). Under these growth conditions, *C. jadinii* cells contained reticulate mitochondrial structures (Fig. 6A). This mitochondrial morphology makes it unlikely that leakage of matrix enzymes during cell fractionation can be prevented. The fluorescent dye used for visualization of mitochondria revealed a single bright spot in approximately half of the cells grown in ethanol-limited *C. jadinii* cultures (Fig. 6A). *C. jadinii* cells that, as a reference, were grown in acetate-limited chemostat cultures showed a similar mitochondrial morphology as the ethanol-grown cultures but did not exhibit the bright spots (Fig. 6A). Cells and, consequently, mitochondria of *O. parapolymorpha* grown in ethanol- and acetate-limited cultures were much smaller than those of *C. jadinii* and did not exhibit bright spots (Fig. 6B).

## DISCUSSION

The biomass yield on ethanol of *C. jadinii* exceeds that of other Complex I positive yeasts (13). This study shows that the exceptionally high biomass yield on ethanol of *C. jadinii* correlates with localization of a substantial fraction of its alcohol dehydrogenase (ADH) content in the mitochondria. Such a predominantly mitochondrial localization of ADH has, to our knowledge, not been described for other yeast species. For example, in the model yeast *S. cerevisiae*, cytosolic Adh2 is the major ethanol-oxidizing ADH isozyme (45). Similarly, in our localization experiments on ethanol-grown *O. parapolymorpha*, only 1.3 ± 0.7% of *in vitro* ADH activity was recovered in the mitochondria enriched fraction of cell homogenates (Table A14).

In the absence of efficient shuttle mechanisms to transfer NADH equivalents across the mitochondrial inner membrane, a solely cytosolic localization of ADH only allows for coupling with non-proton-pumping mitochondrial systems, such as external NADH dehydrogenases and the glycerol-3-phosphate shuttle, for NADH reoxidation (Fig. 1) (20). In contrast, a mitochondrial localization of ADH provides the proton-pumping Complex I with direct access to NADH generated in the first step of ethanol metabolism. An *in vitro* study with *C. jadinii* mitochondria, which showed a two-fold higher ADP/O ratio with ethanol as the electron donor than with externally added NADH (26), is consistent with such strict compartmentation of NADH metabolism.

Cell extracts prepared from ethanol-limited cultures of *C. jadinii* and *O. parapolymorpha* showed similar ADH activities (Table A15). However, while 94% of the detected ADH protein in *C. jadinii* cultures was predicted to have a mitochondrial targeting sequence, this held for less than 2% of the ADH protein in *O. parapolymorpha* cultures grown under the same conditions. Attempts to further substantiate a near-absolute subcellular compartmentation of ADH in the two yeasts by cellular fractionation yielded less conclusive results. Fractionation experiments with *O. parapolymorpha* were consistent with a predominantly cytosolic localization of ADH and mitochondria localization of NAD^+^-dependent ALD. In experiments with *C. jadinii*, ADH did show much higher specific activity in the particulate, mitochondria-enriched fraction than the cytosolic marker enzyme glucose-6-phosphate dehydrogenase, but a large fraction of its total activity was recovered in the soluble, cytosolic fraction. Cellular fractionation by combined enzymic and mechanical cell disruption may damage organelles, resulting in “leakage” of organellar proteins into the soluble (“cytosolic”) fraction of cell homogenates (57). The reticulate mitochondrial structures observed in ethanol-grown *C. jadinii* (Fig. 6) may have been particularly vulnerable to such damage, leading to the release of mitochondrial ADH into the soluble fractions of hydrolysates.

Even if a significant fraction of the ADH in cells is cytosolic, a high intramitochondrial ADH level may enable *C. jadinii* mitochondria to effectively compete for ethanol, especially if the affinities (*V*_max_/*K*_*m*_) of the mitochondrial ADH for ethanol and/or NAD^+^ are higher than those of the cytosolic isozymes. This holds in particular when, as shown in human embryonic kidney cells, the free NAD^+^ concentration in the mitochondrial matrix exceeds that in the cytosol (58). Furthermore, an *in vitro* study on isolated mitochondria indicated that, especially at low mitochondrial NADH concentrations, the respiratory chain of *C. jadinii* prioritizes electrons derived from NADH oxidation by Complex I over those derived from cytosolic NADH (26). A definitive analysis of the localization of the multiple ADH proteins and electron distribution over the electron transfer systems of *C. jadinii* and *O. parapolymorpha* will require molecular studies. These further molecular studies may involve removal or introduction of mitochondrial targeting sequences, fusion of ADH isozymes fluorescent marker proteins, kinetic studies with purified proteins, and engineering of their respiratory chains.

Cell extracts of ethanol-limited chemostat cultures of *C. jadinii* at (*D* = 0.10 h^−1^) showed an *in vitro* ADH activity of 9 µmol·(mg protein)^−1^·min^−1^ (Table A15). Assuming a soluble protein content of yeast biomass of 33% (59), this activity corresponded to an estimated whole-cell ADH capacity of 180 mmol·(g biomass^−1^)·h^−1^. This value is 60-fold higher than the *in vivo* ADH activity in the chemostat cultures, which is equal to the biomass-specific ethanol uptake rate *q*_ethanol_ (2.90 ± 0.03 mmol·g_x_^−1^·h^−1^, Table 1, 46 g∙mol_ethanol_^−1^). In microbial growth kinetics, affinity for a growth-limiting nutrient is defined as *q*_*s*,max_/*K*_*s*_, in which *q*_*s*,max_ indicates its maximum biomass-specific substrate consumption rate and *K*_*s*_ its substrate-saturation (Monod) constant (60). The high ADH capacity in ethanol-limited *C. jadinii* cultures was recently identified as a “*q*_*s*,max_ strategy” (12). This implies that, at low concentrations, affinity for the growth-limiting nutrient is improved by increasing the capacity of the enzyme(s) or transporter(s) that exert control over the biomass-specific rate of substrate consumption (61). A high control of the initial enzyme reaction(s) in ethanol metabolism over its consumption rate is logical for a substrate whose uptake does not involve membrane transport proteins (15). A similar adaptation to nutrient-limited growth on a substrate whose uptake occurs via passive diffusion is provided by the high-level expression of alcohol oxidase in methanol-limited cultures of the methylotrophic yeasts *O. polymorpha* and *Candida boidinii* (62, 63).

Analyzing the impact of subcellular localization of acetaldehyde dehydrogenase (ALD) isozymes on the biomass yield was more complicated than that of ADH due to the ability of different yeast ALD isozymes to use either NAD^+^, NADP^+^, or both as cofactors (48). In both *C. jadinii* and *O. parapolymorpha*, acetaldehyde dehydrogenase activity in cell extracts of ethanol-limited chemostat cultures was predominantly NADP^+^-dependent. This observation agreed with the assumption in the stoichiometric metabolic network model that ALD is the predominant source of NADP^+^.

Upon visualization of mitochondria with a fluorescent dye, intensely fluorescent spots were found in *C. jadinii* cells grown in ethanol-limited chemostat cultures. However, no such bright spots were observed in cells from ethanol-limited *O. parapolymorpha* cultures or from acetate-limited cultures of either yeast (Fig. 6). These spots may indicate the presence of mitochondrial-derived compartments (MDC) which, in *S. cerevisiae*, have been implicated in selective removal and subsequent autophagy of mitochondrial membrane proteins in cells that are aging or subjected to stress (64–66). The specific occurrence of these bright fluorescent spots in ethanol-limited cultures of *C. jadinii* may reflect a higher turnover rate of mitochondrial proteins that might be linked to intramitochondrial ethanol oxidation, for example, through a higher rate of formation of reactive oxygen species (67), acetaldehyde toxicity (68), or a high intramitochondrial ADH protein content. Further research should investigate whether mitochondrial stress and/or protein turnover contribute to the previously reported high maintenance-energy requirements of ethanol-grown *C. jadinii* cultures (12).

In large-scale processes in microbial biotechnology, product yield on the carbon substrate can have a significant impact on overall production costs (69). For anaerobic processes such as yeast-based bioethanol production, in which the cost of the feedstock can account for over half of these costs (70), strategies to maximize product yield on the carbohydrate feedstock have been intensively investigated (71). Understanding the diversity, regulation, and physiology of respiratory energy coupling in yeasts, and improving it by metabolic engineering, is of similar relevance for high-volume manufacturing of products such as yeast biomass as “single-cell protein” for human and animal nutrition. This study identifies the cellular localization of key NAD^+^-dependent dehydrogenases in Complex I-containing yeast species as a relevant topic for future research in this area. From a fundamental science perspective, it raises questions about the evolutionary benefits and trade-offs associated with the different cellular localization patterns of highly expressed enzyme classes such as ADH in different yeast species.

## MATERIALS AND METHODS

### Strains, maintenance, and growth media

Yeast strains used in this study are listed in Table 2. For maintenance, strains were grown in shake flasks on YPD medium. YPD was prepared by autoclaving a solution of 10 g∙L^−1^ yeast extract and 20 g·L peptone^−1^ (Thermo Fisher Scientific, Landsmeer, the Netherlands) in demineralized water at 121°C for 20 min. A sterile 50% (wt/vol) glucose solution, autoclaved separately at 110°C for 20 min, was then aseptically added to achieve a glucose concentration of 20 g∙L^−1^. Sterile glycerol was added to stationary-phase cultures at a final concentration of 30% (vol/vol), and aliquots were stored at −80°C. Synthetic medium (SM) for shake-flask and chemostat cultivation was prepared as described previously (72). For shake-flask cultivation, the pH was adjusted to 6.0 with 2 M KOH prior to autoclaving. For chemostat cultivation, 0.2 mL∙L^−1^ Pluronic 6100 PE antifoam (BASF, Ludwigshafen, Germany) was added prior to autoclaving. Unless otherwise indicated, ethanol was added to a final concentration of 7.5 g∙L^−1^ for shake-flask cultivation and 3.75 g∙L^−1^ for chemostat cultivation, acetic acid was added to a final concentration of 7.5 g∙L^−1^ for chemostat cultivation, and methanol was added to a final concentration of 7.5 g∙L^−1^ for shake-flask cultivation.

**TABLE 2:** Yeast strains used in this study

Species	Strain	Relevant genotype	Origin
*Cyberlindnera jadinii*	CBS 621	Wild type	Westerdijk Institute
*Ogataea parapolymorpha*	CBS 11895	Wild type	Westerdijk Institute
*Ogataea parapolymorpha*	IMD010	*nubm*_G445GC_	(30)
*Ogataea parapolymorpha*	IMD035	*aox*_T35TT_	This study
*Saccharomyces cerevisiae*	CEN.PK113-7D	Prototrophic laboratory reference strain	(73)

Sterile glycerol was added to exponential-phase yeast cultures or overnight *E. coli* cultures at a final concentration of 30% (vol/vol), and aliquots were stored at −80°C.

### Molecular biology techniques and strain construction

XL1‐Blue competent *Escherichia coli* cells (Agilent Technologies, Middelburg, the Netherlands) were transformed according to the manufacturer’s instructions and used for plasmid transformation, amplification, and storage. *E. coli* cells were grown on LB medium (5 g∙L^−1^ Bacto yeast extract, 10 g∙L^−1^ Bacto tryptone, and 10 g∙L^−1^ NaCl) supplemented with 100 mg∙mL^−1^ ampicillin. Plasmids were isolated from *E. coli* using a GenElute Plasmid Miniprep kit (Sigma-Aldrich, Zwijndrecht, the Netherlands). Genomic DNA of yeast colonies used as the template for PCR was isolated using the lithium acetate (LiAc)-sodium dodecyl sulfate method (74). Diagnostic PCR was performed using DreamTaq polymerase (Thermo Fisher Scientific) and 0.25 µM desalted primers (Sigma-Aldrich), forward (20320) 5′-AAAGCGGTATGTCCTTCC-3′ and reverse (20321) 5′-TGCCCTACTTGATCACAG-3′. DNA fragments for sequencing purposes were amplified using Phusion High‐fidelity DNA Polymerase using 0.2 μM primer concentrations, forward (20493) 5′-CCCTCCTGACACAGGTACTC-3′ and reverse (20494) 5′-AGAACAGCAGAGGGCCGATG-3′. DNA fragments obtained by PCR were separated by gel electrophoresis, and PCR purification was performed using a GenElute PCR Clean-Up kit (Sigma-Aldrich).

The Cas9-expressing plasmid carrying a tandem array of gRNAs targeting the alcohol oxidase (*AOX*) gene in *O. parapolymorpha* (pUDP335) was obtained via BsaI-mediated assembly of pUDP002 and pUD1318 as described previously (39) and was confirmed via PCR using forward primer (5721) 5′-CTGCTGTAACCCGTACATGC-3′ and reverse primer (19151) CGGTATCATTGCAGCACTGG. pUD1318 was *de novo* synthesized by GeneArt (Thermo Fisher Scientific) and designed with ribozymes and a linker sequence with the following gRNAs targeting *AOX*: TTCGATATCATTGTTGTTGGTGG and TACTCTACTGCATTGACCATCGG (PAM underlined). *O. parapolymorpha* was transformed with pUDP335 via electroporation and prolonged liquid incubation as described by (39). Hygromycin B was used to select *O. parapolymorpha* transformants in a final concentration of 300 μg∙mL*^−^*^1^. Sanger sequencing (Macrogen Europe, Amsterdam, the Netherlands) of three random colonies no longer able to grow on SM supplemented with methanol revealed an insertion of an A/T base pair at position 35, which resulted in a frame shift and a stop codon at the 42nd codon of *AOX*. One colony was restreaked on YPD agar to lose plasmid pUDP335 and renamed strain IMD035.

### Cultivation techniques

Shake-flask cultures were grown at 30°C in round-bottom flasks with a working volume of 20% of their nominal capacity and shaken at 200 rpm in an Innova incubator (Eppendorf Nederland BV, Nijmegen, the Netherlands).

Carbon-limited, aerobic chemostat cultures were grown at 30°C and at a dilution rate of 0.10 h^−1^ or 0.050 h^−1^ in 1.5 L bioreactors (Getinge-Applikon, Delft, the Netherlands) with a 1 L working volume. The culture pH was maintained at 5.0 by automatic addition of 2 M KOH, controlled by an ez-Control module (Getinge-Applikon). The working volume was kept at 1.0 L by an electrical level sensor that controlled the effluent pump. The precise working volume of chemostat cultures was measured at the end of each experiment by weighing the broth. Cultures were stirred at 800 rpm and sparged with dried, compressed air (0.5 L∙min^−1^). Off-gas flow rates from bioreactors were measured with a Mass-Stream thermal mass flow meter (Bronkhorst, Vlaardingen, the Netherlands). Off gas was cooled to 4°C in a condenser to minimize evaporation and dried with a Perma Pure dryer (Inacom Instruments, Veenendaal, the Netherlands). Concentrations of CO_2_ and O_2_ in the inlet and dried exhaust gas were measured with an analyzer (Servomex MultiExact 4100, Crowborough, United Kingdom). Cultures were assumed to have reached steady state when, after a minimum of 5 volume changes, CO_2_ production rate, biomass concentration, and high-performance liquid chromatography (HPLC) measurements changed by less than 5% over two consecutive volume changes.

### Analytical methods

For biomass dry weight determination, duplicate culture samples of exactly 10.0 mL were filtered over pre-dried and pre-weighed membrane filters (0.45 μm pore size; Pall Corporation, Ann Arbor, MI, USA). Filters were then washed with demineralized water, dried in a microwave oven at 320 W for 20 min, and immediately reweighed (59).

Metabolite concentrations in culture supernatants and media were measured with an Agilent 1260 Infinity HPLC system (Agilent Technologies) equipped with a Bio-Rad Aminex HPX-87H ion exchange column (Bio-Rad, Hercules, CA, USA), operated at 60°C with 5 mM H_2_SO_4_ as mobile phase at a flow rate of 0.600 mL∙min^−1^. Culture supernatant was obtained by centrifuging 1 mL aliquots at 13,000 rpm for 5 min with a Biofuge Pico centrifuge (Heraeus Instruments, Hanau, Germany). Metabolite concentrations in steady-state chemostat cultures were analyzed after rapid quenching of culture samples with cold steel beads (75).

### Fluorescence microscopy

For fluorescence microscopy (FM) studies, all cells were diluted to an OD_660_ of 2 and incubated with MitoTracker for 40 min at 30°C with 200 rpm shaking. The cells were subsequently concentrated at 3,000 × *g* for 1 min, resuspended in SM medium, and imaged. *C. jadinii* cells were incubated with MitoTracker DeepRed FM (invitrogen #M22426) to a final concentration of 500 nM. For *O. parapolymorpha*, cells were incubated with MitoTracker Orange CMTMRos (invitrogen #M7510) to a final concentration of 100 nM. All cells were spotted and imaged under a 1% agar pad containing SM medium with ethanol or acetate as a carbon source. Cells were imaged at room temperature.

For FM imaging, a Zeiss Axio Imager Z1 (Carl Zeiss AG, Oberkochen, Germany) was equipped with a HAL 100 halogen illuminator, HBO 100 illuminating system, and AxioCam HRm Rev3 detector (60N-C 1″ 1.0×) (Carl Zeiss AG). The 100×/1.3 oil immersion objective was used with Immersol 518F type F immersion oil (Carl Zeiss AG). Filter set 14 was used to detect fluorescence from MitoTracker Orange CMTMRos with an excitation of 551 nm and emission at 575 nm. Filter set 50 was used to detect fluorescence from MitoTracker DeepRed FM with an excitation of 641 nm and emission at 662 nm. Filter set 09 was used to detect fluorescence in the green spectrum with an excitation of 493 nm and emission at 517 nm. Filter 49 was used to detect fluorescence in the blue spectrum with an excitation of 392 nm and emission at 455 nm. Results were analyzed using the Fiji v1.54F package for ImageJ.

### Preparation of cell-free extracts

Cells harvested from shake-flask or steady-state chemostat cultures were harvested by low-speed centrifugation (3,900 rpm (3,180 × *g*), 10 min, 4°C) in an Eppendorf 5810 R centrifuge (Eppendorf), washed once with 20 mL ice-cold freeze buffer (10 mM potassium-phosphate buffer [KPB], pH 7.5 + 2 mM ethylenediaminetetraacetic acid [EDTA]), centrifuged again (3,900 rpm (3,180 × *g*), 5 min, 4°C), and resuspended in 4 mL ice-cold freeze buffer before storing as cell suspensions at −20°C.

On the day of analysis, cells were thawed at room temperature (RT) and harvested by low-speed centrifugation (3,900 rpm (3,180 × *g*), 5 min, 4°C), washed once with 20 mL ice-cold sonication buffer (100 mM KPB, pH 7.5 + 2 mM MgCl_2_), centrifuged again (3,900 rpm (3,180 × *g*), 5 min, 4°C), and resuspended in 4 mL ice-cold sonication buffer + 1 mM 1,4-dithiothreitol (DTT). For alcohol oxidase assays, no DTT was added, but protease inhibitors (Complete Protease inhibitor cocktail, Merck group, Darmstadt, Germany) were used following the manufacturer’s instructions.

Cells were disrupted by sonication at 4°C with an MSE Soniprep 150 sonicator (output, 7–8 μm peak-to-peak amplitude) with a pre-cooled probe at 30-second cycles (sonication – cooling) (59). *S. cerevisiae*, *C. jadinii*, and *O. parapolymorpha* cells were sonicated using 425–600 μm diameter glass beads (approximately 3.2 g) at 0°C for 4, 6, and 8 cycles, respectively. For alcohol oxidase assays, cells from *O. parapolymorpha* were disrupted with a Fast Prep FP120 (MP Biomedicals, Fisher Scientific, Hampton, NH, USA) for six bursts of 20 s, at a speed setting of 6 m·s^−1^. Samples were cooled on ice in between cycles.

Unbroken cells and debris were removed by centrifugation at 4°C (20,000 rpm (7,800 × *g*), 20 min) using a JA25.50 rotor in a Beckman Coulter Avanti J-E series high-speed centrifuge (Beckman Coulter Life Sciences, Woerden, the Netherlands). The clear supernatant, which usually contained 0.4–3.0 mg_protein_·mL^−1^, was kept on ice and was used as cell-free extract directly after preparation.

### Preparation of subcellular fractions

Mitochondria-enriched fractions were prepared from ethanol-limited, aerobic chemostat cultures by a combination of protocols with a few modifications (22, 56, 76).

Stock solutions were used to prepare working solutions and were sterilized prior to use at 120°C for 20 min unless stated otherwise. The following stock solutions were prepared and stored in aliquots: Tris buffer (Tris, 100 mM), 0.5 M potassium phosphate buffer (KPB) (MgCl_2_, 20 mM; EDTA, 20 mM; pH 7.5), and a sorbitol stock solution (3 M, autoclaved at 110°C for 20 minutes). Working solutions were prepared freshly prior to use. The following working solutions were prepared: Tris-DTT solution (Tris buffer; DTT, 10 mM, pH 9.3), buffer A (KPB, 25 mM; MgCl_2_, 1 mM; EDTA, 1 mM; sorbitol, 2 M; pH 7.5), buffer B (KPB, 25 mM; MgCl_2_, 1 mM; EDTA, 1 mM; sorbitol, 0.2 M; pH 7.5), and buffer C (KPB, 25 mM; MgCl_2_, 1 mM; EDTA, 1 mM; sorbitol, 1 M; pH 7.5).

Biomass (approximately 1.5 g dry weight) of *C. jadinii* and *O. parapolymorpha* was harvested directly from steady-state cultures (dilution rate 0.10 h^−1^) by low-speed centrifugation (3,900 rpm (3,180 × *g*), 5 min, 4°C) in conical Corning 500 mL centrifuge tubes in an Eppendorf 5810 R centrifuge. Pellet was then resuspended in 30 mL of Tris-DTT solution by gentle vortexing and incubated at 30°C for 10 min. After centrifugation (3,900 rpm/3,162 × *g*, 5 min, 4°C), the pellet was washed with 35 mL buffer A, centrifuged again (3,900 rpm (3,180 × *g*), 10 min, 4°C), and resuspended gently in 40 mL buffer A using 5 mL pipette tips with cut tip.

Spheroplasts were obtained by adding 5 mg Zymolyase 20T (AMSbio Europe, Alkmaar, the Netherlands) per 1.5 g pellet wet weight. Spheroplasting was monitored via the decrease in OD_660_ at 10 min intervals by taking 20 μL of sample and diluting this in 4 mL of demineralized water using a Libra S12 Spectrophotometer (Biochrom, Cambridge, United Kingdom). Spheroplasting was halted after an OD_660_ drop of 75–80% (*C. jadinii* ca. 60 min, *O. parapolymorpha* ca. 40 min), after which the spheroplasts were kept on ice.

Spheroplasts were harvested by centrifugation at 4°C (6,000 rpm (2,800 × *g*), 7 min) using a JA25.50 rotor in a Beckman Coulter Avanti J-E series high-speed centrifuge, washed with 30 mL buffer A, centrifuged again (6,000 rpm (2,800 × *g*), 7 min), and resuspended in buffer A to a final volume of 10–15 mL. The volume was determined using a serological pipette to calculate the appropriate amount of buffer B to dropwise add to the suspension while slowly stirred with a magnetic stirrer bar at 4°C for the suspension to reach 1 M sorbitol. The spheroplast suspension was homogenized at 4°C using a pre-cooled Potter-Elvehjem PTFE pestle by subjecting it to 10 strokes (100 rpm, clearance 38 μm) in a glass tube with a working volume of 30 mL. The homogenate was cleared of unbroken cells and cellular debris by centrifugation (5,000 rpm (2,000 × *g*), 10 min). The homogenate was removed using a Pasteur pipette, transferred to a clean tube, and centrifuged again (20,000 rpm (7,800 × *g*), 10 min). The supernatant containing the cytosolic fraction was collected in a separate tube. The resulting pellet, containing the mitochondria-enriched fraction, was resuspended in 4 mL of buffer C. Homogenate, cytosolic fraction, and mitochondrial fractions were processed immediately for enzymatic assays, and volumes of the suspensions were used for further calculations on fractionation.

### Enzymatic activity assays

Spectrophotometric assays were carried out with freshly prepared cell-free extracts, homogenate, or enriched subcellular fractions, using a Hitachi model U-3010 spectrophotometer (Sysmex Europe GmbH, Norderstedt, Germany). All enzyme assays were carried out at 30°C in a Quartz cuvette with pathlength of 1 cm, except the alcohol oxidase assays which were performed using disposable 1.5 mL cuvettes. In all assays, reaction velocity was verified to be linearly proportional to the amount of cell-free extract or enriched fraction present. Activity was measured after running the reaction for an initial 2 minutes to determine endogenous activity in the reaction mixture. Assay mixtures for individual enzymes are described below; concentrations depicted are final concentrations in 1 mL in the cuvette.

#### Alcohol dehydrogenase (EC 1.1.1.1)

As described in reference 59, the reaction mixture contained glycine-KOH (pH 9.0, 50 mM), NAD^+^ (1 mM), and cell-free extract, enriched subcellular fraction, or homogenate. The reaction was initiated by the addition of ethanol (100 mM).

#### Alcohol oxidase (EC 1.1.3.13)

Based on reference 77, the reaction mixture contained solution A and cell-free extract. The reaction was started by the addition of methanol (125 mM) or ethanol (125 mM). Solution A was prepared fresh on the day of use and contained potassium phosphate buffer (pH 7.2, 500 mM), 2,2'-azino-bis (3-ethylbenzothiazoline-6-sulfonic acid) (ABTS; 1 tablet per 100 mL buffer to prevent solubility issues), and horseradish peroxidase (250 units·mg^−1^, 10 mg·mL^−1^, 80 μL per 100 mL buffer). Solution A was centrifuged (3,900 rpm (3,180 × *g*), 3 min, 4 °C) to get rid of ABTS-containing tablet debris.

#### Acetaldehyde dehydrogenase (NAD(P)^+^-dependent) (EC 1.2.1.3/1.2.1.4)

The reaction mixture contained potassium phosphate buffer (pH 8.0, 100 mM), KCl (10 mM), pyrazole (15 mM), dithiothreitol (DTT; 0.4 mM), NAD(P)^+^ (0.4 mM), and cell-free extract, enriched subcellular fraction, or homogenate. The procedure was as previously described (59), except the acetaldehyde concentration was increased to 7 mM and was prepared fresh on the day of analysis from a stock solution.

#### Cytochrome c oxidase (EC 1.9.3.1)

Based on reference 56, the reaction mixture contained 0.8 mg∙mL^−1^ reduced cytochrome c from horse heart. The reaction was started by the addition of cell-free extract, enriched subcellular fraction, or homogenate. Cytochrome c was dissolved in 50 mM potassium phosphate buffer (pH 7.0) to a concentration of 10 mg∙mL^−1^, reduced by the addition of sodium dithionite, and subsequently purified using a PD10 column (Sigma Aldrich).

#### Glucose-6-phosphate dehydrogenase (EC 1.1.1.49)

As described in reference 59, the reaction mixture contained: Tris-HCl buffer (pH 8.0, 50 mM), MgCl_2_ (5 mM), NADP^+^ (0.4 mM), and cell-free extract, enriched subcellular fraction, or homogenate. The reaction was started by the addition of glucose 6-phosphate (5 mM).

Reduction rate of NAD(P)^+^ was measured at 340 nm, using an extinction coefficient of 6.3 mM^−1^ for specific activity determination. Oxidation rate of cytochrome c was measured at 550 nm, using an extinction coefficient of 21.1 mM^−1^. Oxidation rate of ABTS_red_ was measured at 420 nm, using an extinction coefficient of 43.2 mM^−1^. Specific activities of the various enzymes are expressed as μmol∙min^−1^∙mg_protein_^−1^ and are the average of at least two determinations.

Protein concentrations in cell-free extracts, enriched subcellular fractions, or homogenate were determined by the Lowry method (78), using bovine serum albumin (BSA, Sigma Aldrich) 1 mg∙mL^−1^ as a standard, after freezing samples at −20°C for at least one night.

### Genomic analysis of mitochondrial targeting sequences

Publicly available genomes of *C. jadinii* (GCA_001245095.1 on NCBI) (49) and *O. parapolymorpha* (GCF_000187245.1 on NCBI) (40) were selected for their high assembly level for the detection of alcohol and aldehyde dehydrogenase genes. Protein sequences retrieved from the Saccharomyces genome database (SGD, URL https://www.yeastgenome.org/) were used as queries in a tBLASTn search on the NCBI website (URL https://www.ncbi.nlm.nih.gov/) using the default settings. The presence of an MTS was assessed for each gene product annotated in NCBI at the identified regions homologous to the *S. cerevisiae* protein sequences, unless otherwise indicated, using the algorithms TargetP (URL https://services.healthtech.dtu.dk/services/TargetP-2.0/) (50) and DeepMito (URL https://busca.biocomp.unibo.it/deepmito/) (51).

### Proteome analysis

Biomass samples from steady-state, aerobic, ethanol-limited chemostats operated at a dilution rate of 0.10 h^−1^ (25 mL, containing 2.5–2.7 g_biomass_·L^−1^ for both yeasts) were centrifuged and washed once with demineralized water. Pellets were resuspended in cold methanol (−20°C) and lysed with a Precellys homogenizer (Bertin technologies SAS, Montigny-le-Bretonneux, France) at 6,800 rpm for 2 bursts of 20 s separated by a 30 s interval. Samples were normalized based on their total protein concentration, measured by Qubit protein assay. Proteins were processed further by reduction, alkylation, and digestion using trypsin (79).

Samples were analyzed in technical triplicates by liquid chromatography coupled to tandem mass spectrometry (LC–MS/MS) using a Vanquish UHPLC coupled to an Orbitrap Exploris 480 mass spectrometer (Thermo Fisher Scientific, Waltham, USA). Peptides were separated using reverse-phase chromatography with a gradient of water with 0.1% formic acid (solvent A) and 20% water and 0.1% formic acid in acetonitrile (solvent B) from 5% B to 40% B in 20 min. Data-independent acquisition (DIA) was performed with a resolution setting of 60,000 within the 350 to 1,200 m/z range with a normalized AGC target of 300% and a maximum injection time set to automatic, followed by high-energy collision-induced dissociation activated (HCD) MS/MS scans performed with a resolution setting of 15,000 and injection time set to auto. Twelve DIA MS/MS fragmentation scans were performed after each full scan, and the mass range was divided into 17 m/z windows covering 400 to 1000 m/z range.

Raw files were analyzed with the Pulsar search engine and Spectronaut, version 19.7, against the annotated proteins of *C. jadinii* (UniProtKB, strain ATCC 18201/CBS 1600, URL https://www.uniprot.org) and *O. parapolymorpha* (UniProtKB, strain DL-1). Label-free quantification was performed using the top three peptides measured for each protein. Search settings were trypsin-specific with a minimal peptide length of six and a maximum length of 30 amino acids with two missed cleavages allowed. Variable modifications of protein N-terminal acetylation, carbamidomethylation of cysteines, deamidation of asparagine and glutamine, and oxidation of methionine were included. Direct DIA label-free quantification was performed using the top three peptides measured for each protein. Results were filtered at a false discovery rate of 1%. Retention time realignment was based on local (non-linear) regression.

The obtained signal per protein (raw signal [*i*], proportional to protein copy number) was multiplied by the respective molecular weight (obtained from UniProt), yielding a signal (*i*). Subsequently, the signal (*i*) obtained per protein was divided by the sum of all signals (*i*) obtained in the sample and multiplied by 100. This resulted in the approximate percentage (in weight/weight) by which each protein contributed to the total proteome mass. Approximate percentages per protein were averaged from three technical replicates. Proteins with a raw signal of zero for all conditions were removed from the data set.

The presence of an MTS was assessed for each ADH and ALD identified using the algorithms MitoProt (URL https://ihg.helmholtz-munich.de/ihg/mitoprot.html) (54), TargetP (URL https://services.healthtech.dtu.dk/services/TargetP-2.0/) (50), and DeepMito (URL https://busca.biocomp.unibo.it/deepmito/) (51).

The data is made available as Supplemental Material 5.

### Stoichiometric analysis with a core metabolic network model

A core yeast metabolic stoichiometric model consisting of 169 reactions × 165 compounds divided over three compartments was adapted from reference 13 (Supplemental Material 1 through 4, https://github.com/mwarmerdam/localization-code). The matrix was solved with different sets of constraints to simulate the effect on biomass yield of different subcellular localization scenarios using Python 3.12.11 (code provided as Supplemental Material 3). The model with constraints that represent *S. cerevisiae* (cytosolic ADH and ALD) has been expanded to include glucose metabolism and converted into a .json file compatible with cobra and is made available as Supplemental Material 4.

### Data analysis

Calculations involved in establishing bioreactor carbon balances, individual protein contributions to the total proteome mass, specific enzymatic activities, and expressing model solutions as yields in gram dry weight were performed in Microsoft Excel. Data visualization and statistical analysis were performed with GraphPad Prism version 10.6.1. Raw enzymatic activity was determined with UV solutions version 2.0.

## SUPPLEMENTAL MATERIAL

Supplemental Material 1 aem.00362-26-s0001.docx Tables A1-A15 and Fig. A1-A2.

Supplemental Material 2: Model aem.00362-26-s0002.xlsx Stoichiometric matrix (tab "S"), boundary conditions (tabs "Bounds_A-N"), underlying calculations (tabs "Cmol" and "val"), model solutions ("Results"), and summary of main results simulations (tab "SUMMARY").

Supplemental Material 3: Model (Python) aem.00362-26-s0003.pdf Core yeast metabolic stoichiometric model – code compatible with Python.

Supplemental Material 4: Model (Cobra) aem.00362-26-s0004.pdf Core yeast metabolic stoichiometric model – code compatible with Cobra.

Supplemental Material 5: Proteome data aem.00362-26-s0005.xlsx Raw signals obtained from proteome analysis and subsequent analysis.

ASM does not own the copyrights to Supplemental Material that may be linked to, or accessed through, an article. The authors have granted ASM a non-exclusive, world-wide license to publish the Supplemental Material files. Please contact the corresponding author directly for reuse.
