# Supplementary material for: Mitochondrial localization of key NAD+-dependent dehydrogenases explains the exceptionally high biomass yield on ethanol of the yeast Cyberlindnera jadinii
Source: Appl Environ Microbiol. 2026 Jul 10;92(8):e00362-26. doi: 10.1128/aem.00362-26 (PMC13488300; doi:10.1128/aem.00362-26)
Supplement: Supplementary file 1 [file aem.00362-26-s0003.pdf]

Supplemental material 3 - Warmerdam et al. 2026
Text should be saved as .ipynb or .py

```
# First import all packages required
import numpy as np
import numpy.linalg as linalg
from scipy.optimize import linprog
from scipy.linalg import null_space
import matplotlib.pyplot as plt
import pandas as pd


'''Set path to open Excel file'''
path = 'Model (Supplemental information 2).xlsx'

''' Retreive irreversibilities and constraints from file defined in 'bounds' '''
data = pd.read_excel(path, "Bounds_A", header=0, index_col=0)
bounds_data  = np.array(data)       # Bounds matrix
bounds_data  = bounds_data[:169, :2]
# Bounds matrix this needs to match the number of reactions

''' Extract Stoichiometric matrix from Excel file '''
df = pd.read_excel(path, "S", header=0, index_col=0)
S  = np.array(df)       # S matrix
v_lab = list(df)        # List of reactions
m_lab = list(df.index)  # List of metabolites

# Number of reactions and compounds
n_reactions = S.shape[1]
n_compounds = S.shape[0]

# Rank of the stoichiometric matrix
rank_S = np.linalg.matrix_rank(S)

# Calculate degrees of freedom
degrees_of_freedom = n_reactions - rank_S

# Display the result
print(f"Number of reactions: {n_reactions}")
print(f"Number of compounds: {n_compounds}")
print(f"Rank of the stoichiometric matrix: {rank_S}")
print(f"Degrees of freedom: {degrees_of_freedom}")

A_eq = S
#q = np.array([1])                                # q rates
b_eq = np.zeros(S.shape[0])
```

```python
#b_eq = np.concatenate( ( zeros , q ) )

''' Optimization target'''
# Define a 'c' vector with target of optimization
target = v_lab.index('ETOH:ext')
c = np.zeros( n_reactions )
c[target] = -1                                    # Optimize for target flux (min(-c.v))

# Perform optimization with linprog
opt_res = linprog( c, A_eq=A_eq, b_eq=b_eq, bounds = bounds_data)
#print(opt_res)

# opt.res contains all relevant information as a structure
v_opt = opt_res.x

[print( '%s = %.2f ' % (v_lab[i], v_opt[i]) ) for i in range(n_reactions) ];

# Round the flux values down to two decimals
v_opt_rounded = np.round(v_opt, 2)

# Generate a DataFrame with flux names and rounded flux values
df = pd.DataFrame({
    'Fluxnaam': v_lab,
    'Fluxrate': v_opt_rounded
})

# Save the DataFrame as an Excel file
df.to_excel("FILENAME.xlsx", index=False)
print("Results saved")
```
