# Supplementary material for: Mitochondrial localization of key NAD+-dependent dehydrogenases explains the exceptionally high biomass yield on ethanol of the yeast Cyberlindnera jadinii
Source: Appl Environ Microbiol. 2026 Jul 10;92(8):e00362-26. doi: 10.1128/aem.00362-26 (PMC13488300; doi:10.1128/aem.00362-26)
Supplement: Supplementary file 2 [file aem.00362-26-s0004.pdf]

Text should be saved as .json file

{
"metabolites":[
{
"id":"ADP_c",
"name":"",
"compartment":"c"
},
{
"id":"ADP_m",
"name":"",
"compartment":"c"
},
{
"id":"ATP_c",
"name":"",
"compartment":"c"
},
{
"id":"ATP_m",
"name":"",
"compartment":"c"
},
{
"id":"biom_e",
"name":"",
"compartment":"c"
},
{
"id":"CARBHYD_c",
"name":"",
"compartment":"c"
},
{
"id":"DNA_c",
"name":"",
"compartment":"c"
},
{
"id":"LIPID_c",
"name":"",
"compartment":"c"
},
{
"id":"PROT_c",
"name":"",
"compartment":"c"
},
{
"id":"RNA_c",
"name":"",
"compartment":"c"
},
{
"id":"13PG_c",

"name":"",
"compartment":"c"
},
{
"id":"3PG_c",
"name":"",
"compartment":"c"
},
{
"id":"2PG_c",
"name":"",
"compartment":"c"
},
{
"id":"A_c",
"name":"",
"compartment":"c"
},
{
"id":"AMP_c",
"name":"",
"compartment":"c"
},
{
"id":"H_c",
"name":"",
"compartment":"c"
},
{
"id":"ACCoA_c",
"name":"",
"compartment":"c"
},
{
"id":"CO2_c",
"name":"",
"compartment":"c"
},
{
"id":"H2O_c",
"name":"",
"compartment":"c"
},
{
"id":"MACoA_c",
"name":"",
"compartment":"c"
},
{
"id":"Pi_c",
"name":"",
"compartment":"c"
},
{

"id":"ACT_c",
"name":"",
"compartment":"c"
},
{
"id":"CoA_c",
"name":"",
"compartment":"c"
},
{
"id":"ACTAL_c",
"name":"",
"compartment":"c"
},
{
"id":"NAD_c",
"name":"",
"compartment":"c"
},
{
"id":"NADH_c",
"name":"",
"compartment":"c"
},
{
"id":"NADP_c",
"name":"",
"compartment":"c"
},
{
"id":"NADPH_c",
"name":"",
"compartment":"c"
},
{
"id":"ACT_m",
"name":"",
"compartment":"c"
},
{
"id":"H_m",
"name":"",
"compartment":"c"
},
{
"id":"ACTAL_m",
"name":"",
"compartment":"c"
},
{
"id":"AICAR_c",
"name":"",
"compartment":"c"
},

{
"id":"ASP_c",
"name":"",
"compartment":"c"
},
{
"id":"FTHF_c",
"name":"",
"compartment":"c"
},
{
"id":"FUM_c",
"name":"",
"compartment":"c"
},
{
"id":"GLM_c",
"name":"",
"compartment":"c"
},
{
"id":"GLN_c",
"name":"",
"compartment":"c"
},
{
"id":"GLY_c",
"name":"",
"compartment":"c"
},
{
"id":"PPi_c",
"name":"",
"compartment":"c"
},
{
"id":"PRPP_c",
"name":"",
"compartment":"c"
},
{
"id":"THF_c",
"name":"",
"compartment":"c"
},
{
"id":"ALA_c",
"name":"",
"compartment":"c"
},
{
"id":"OGL_c",
"name":"",
"compartment":"c"

},
{
"id":"PYR_c",
"name":"",
"compartment":"c"
},
{
"id":"IMP_c",
"name":"",
"compartment":"c"
},
{
"id":"ARG_c",
"name":"",
"compartment":"c"
},
{
"id":"CARP_c",
"name":"",
"compartment":"c"
},
{
"id":"ORN_c",
"name":"",
"compartment":"c"
},
{
"id":"ASN_c",
"name":"",
"compartment":"c"
},
{
"id":"HSER_c",
"name":"",
"compartment":"c"
},
{
"id":"OXACT_c",
"name":"",
"compartment":"c"
},
{
"id":"avAA_c",
"name":"",
"compartment":"c"
},
{
"id":"CYS_c",
"name":"",
"compartment":"c"
},
{
"id":"HIS_c",
"name":"",

"compartment":"c"
},
{
"id":"ILE_c",
"name":"",
"compartment":"c"
},
{
"id":"LEU_c",
"name":"",
"compartment":"c"
},
{
"id":"LYS_c",
"name":"",
"compartment":"c"
},
{
"id":"MET_c",
"name":"",
"compartment":"c"
},
{
"id":"PHE_c",
"name":"",
"compartment":"c"
},
{
"id":"PRO_c",
"name":"",
"compartment":"c"
},
{
"id":"SER_c",
"name":"",
"compartment":"c"
},
{
"id":"THR_c",
"name":"",
"compartment":"c"
},
{
"id":"TRP_c",
"name":"",
"compartment":"c"
},
{
"id":"TYR_c",
"name":"",
"compartment":"c"
},
{
"id":"VAL_c",

"name":"",
"compartment":"c"
},
{
"id":"avFA-CoA_c",
"name":"",
"compartment":"c"
},
{
"id":"OLE-CoA_c",
"name":"",
"compartment":"c"
},
{
"id":"PLLM-CoA_c",
"name":"",
"compartment":"c"
},
{
"id":"PLM-CoA_c",
"name":"",
"compartment":"c"
},
{
"id":"STE-CoA_c",
"name":"",
"compartment":"c"
},
{
"id":"avPL_c",
"name":"",
"compartment":"c"
},
{
"id":"PHD-CHO_c",
"name":"",
"compartment":"c"
},
{
"id":"PHD-ETA_c",
"name":"",
"compartment":"c"
},
{
"id":"PHD-SER_c",
"name":"",
"compartment":"c"
},
{
"id":"G6P_c",
"name":"",
"compartment":"c"
},
{

"id":"CDP_c",
"name":"",
"compartment":"c"
},
{
"id":"CTP_c",
"name":"",
"compartment":"c"
},
{
"id":"CIT_c",
"name":"",
"compartment":"c"
},
{
"id":"CMP_c",
"name":"",
"compartment":"c"
},
{
"id":"CO2_m",
"name":"",
"compartment":"c"
},
{
"id":"UTP_c",
"name":"",
"compartment":"c"
},
{
"id":"HCYS_c",
"name":"",
"compartment":"c"
},
{
"id":"NH4_c",
"name":"",
"compartment":"c"
},
{
"id":"OBU_c",
"name":"",
"compartment":"c"
},
{
"id":"DHF_c",
"name":"",
"compartment":"c"
},
{
"id":"GTP_c",
"name":"",
"compartment":"c"
},

{
"id":"METHF_c",
"name":"",
"compartment":"c"
},
{
"id":"PEP_c",
"name":"",
"compartment":"c"
},
{
"id":"H2O2_c",
"name":"",
"compartment":"c"
},
{
"id":"H2O2_e",
"name":"",
"compartment":"c"
},
{
"id":"ETOH_c",
"name":"",
"compartment":"c"
},
{
"id":"O2_c",
"name":"",
"compartment":"c"
},
{
"id":"ETOH_m",
"name":"",
"compartment":"c"
},
{
"id":"DHAP_c",
"name":"",
"compartment":"c"
},
{
"id":"F16P_c",
"name":"",
"compartment":"c"
},
{
"id":"GAP_c",
"name":"",
"compartment":"c"
},
{
"id":"F6P_c",
"name":"",
"compartment":"c"

},
{
"id":"FAT_c",
"name":"",
"compartment":"c"
},
{
"id":"PHD_c",
"name":"",
"compartment":"c"
},
{
"id":"MAL_c",
"name":"",
"compartment":"c"
},
{
"id":"RIBU5P_c",
"name":"",
"compartment":"c"
},
{
"id":"GDP_c",
"name":"",
"compartment":"c"
},
{
"id":"GMP_c",
"name":"",
"compartment":"c"
},
{
"id":"GOH3P_c",
"name":"",
"compartment":"c"
},
{
"id":"GOH_c",
"name":"",
"compartment":"c"
},
{
"id":"H2O_m",
"name":"",
"compartment":"c"
},
{
"id":"HCIT_c",
"name":"",
"compartment":"c"
},
{
"id":"H2S_c",
"name":"",

"compartment":"c"
},
{
"id":"GLUC_c",
"name":"",
"compartment":"c"
},
{
"id":"GLUC_e",
"name":"",
"compartment":"c"
},
{
"id":"ICIT_c",
"name":"",
"compartment":"c"
},
{
"id":"GLYO_c",
"name":"",
"compartment":"c"
},
{
"id":"SUC_c",
"name":"",
"compartment":"c"
},
{
"id":"IPM_c",
"name":"",
"compartment":"c"
},
{
"id":"OIV_c",
"name":"",
"compartment":"c"
},
{
"id":"OAD_c",
"name":"",
"compartment":"c"
},
{
"id":"SAM_c",
"name":"",
"compartment":"c"
},
{
"id":"MYTHF_c",
"name":"",
"compartment":"c"
},
{
"id":"O2_m",

"name":"",
"compartment":"c"
},
{
"id":"CMP-DGOH_c",
"name":"",
"compartment":"c"
},
{
"id":"SAH_c",
"name":"",
"compartment":"c"
},
{
"id":"CHO_c",
"name":"",
"compartment":"c"
},
{
"id":"PLM_c",
"name":"",
"compartment":"c"
},
{
"id":"RIB5P_c",
"name":"",
"compartment":"c"
},
{
"id":"XYL5P_c",
"name":"",
"compartment":"c"
},
{
"id":"E4P_c",
"name":"",
"compartment":"c"
},
{
"id":"SO4_c",
"name":"",
"compartment":"c"
},
{
"id":"STE_c",
"name":"",
"compartment":"c"
},
{
"id":"SED7P_c",
"name":"",
"compartment":"c"
},
{

"id":"CIT_m",
"name":"",
"compartment":"c"
},
{
"id":"OXACT_m",
"name":"",
"compartment":"c"
},
{
"id":"ICIT_m",
"name":"",
"compartment":"c"
},
{
"id":"UDP_c",
"name":"",
"compartment":"c"
},
{
"id":"UMP_c",
"name":"",
"compartment":"c"
},
{
"id":"ACT_e",
"name":"",
"compartment":"c"
},
{
"id":"H_e",
"name":"",
"compartment":"c"
},
{
"id":"CO2_e",
"name":"",
"compartment":"c"
},
{
"id":"ETOH_e",
"name":"",
"compartment":"c"
},
{
"id":"GOH_e",
"name":"",
"compartment":"c"
},
{
"id":"H2O_e",
"name":"",
"compartment":"c"
},

{
"id":"NH4_e",
"name":"",
"compartment":"c"
},
{
"id":"O2_e",
"name":"",
"compartment":"c"
},
{
"id":"Pi_e",
"name":"",
"compartment":"c"
},
{
"id":"SO4_e",
"name":"",
"compartment":"c"
},
{
"id":"HCIT_m",
"name":"",
"compartment":"c"
},
{
"id":"ILE_m",
"name":"",
"compartment":"c"
},
{
"id":"ACO_m",
"name":"",
"compartment":"c"
},
{
"id":"NAD_m",
"name":"",
"compartment":"c"
},
{
"id":"NADH_m",
"name":"",
"compartment":"c"
},
{
"id":"NADP_m",
"name":"",
"compartment":"c"
},
{
"id":"NADPH_m",
"name":"",
"compartment":"c"

},
{
"id":"Pi_m",
"name":"",
"compartment":"c"
},
{
"id":"ACCoA_m",
"name":"",
"compartment":"c"
},
{
"id":"CoA_m",
"name":"",
"compartment":"c"
},
{
"id":"FUM_m",
"name":"",
"compartment":"c"
},
{
"id":"MAL_m",
"name":"",
"compartment":"c"
},
{
"id":"OGL_m",
"name":"",
"compartment":"c"
},
{
"id":"GLM_m",
"name":"",
"compartment":"c"
},
{
"id":"OBU_m",
"name":"",
"compartment":"c"
},
{
"id":"PYR_m",
"name":"",
"compartment":"c"
},
{
"id":"OAD_m",
"name":"",
"compartment":"c"
},
{
"id":"SUCCOA_m",
"name":"",

"compartment":"c"
},
{
"id":"OIV_m",
"name":"",
"compartment":"c"
},
{
"id":"ORN_m",
"name":"",
"compartment":"c"
},
{
"id":"Q_m",
"name":"",
"compartment":"c"
},
{
"id":"QH2_m",
"name":"",
"compartment":"c"
},
{
"id":"SUC_m",
"name":"",
"compartment":"c"
},
{
"id":"NH4_m",
"name":"",
"compartment":"c"
},
{
"id":"THR_m",
"name":"",
"compartment":"c"
},
{
"id":"VAL_m",
"name":"",
"compartment":"c"
}
],
"reactions":[
{
"id":"ADPc_t_ATPm",
"name":"",
"metabolites":{
"ADP_c":-1.0,
"ADP_m":1.0,
"ATP_c":1.0,
"ATP_m":-1.0
},
"lower_bound":-1000.0,

"upper_bound":1000.0,
"gene_reaction_rule":""
},
{
"id":"biom-Cj",
"name":"",
"metabolites":{
"CARBHYD_c":-0.196135720815087,
"DNA_c":-0.00376996435388721,
"LIPID_c":-0.0978631912214467,
"PROT_c":-0.61912820143726,
"RNA_c":-0.0742847403541373,
"biom_e":1.0
},
"lower_bound":0.0,
"upper_bound":0.0,
"gene_reaction_rule":""
},
{
"id":"biom-Op",
"name":"",
"metabolites":{
"CARBHYD_c":-0.307869563321834,
"DNA_c":-0.0038220895955011,
"LIPID_c":-0.102466908906771,
"PROT_c":-0.549072235813134,
"RNA_c":-0.0670093090771256,
"biom_e":1.0
},
"lower_bound":0.0,
"upper_bound":0.0,
"gene_reaction_rule":""
},
{
"id":"biom-Sc",
"name":"",
"metabolites":{
"CARBHYD_c":-0.257581114809301,
"DNA_c":-0.00375400198135734,
"LIPID_c":-0.129988688082063,
"PROT_c":-0.445540836546251,
"RNA_c":-0.048101129350554,
"biom_e":1.0
},
"lower_bound":-1000.0,
"upper_bound":1000.0,
"gene_reaction_rule":""
},
{
"id":"c13PG_kin",
"name":"",
"metabolites":{
"13PG_c":-1.0,
"3PG_c":1.0,

"ADP_c":-1.0,
"ATP_c":1.0
},
"lower_bound":-1000.0,
"upper_bound":1000.0,
"gene_reaction_rule":""
},
{
"id":"c3PG_mut",
"name":"",
"metabolites":{
"2PG_c":1.0,
"3PG_c":-1.0
},
"lower_bound":-1000.0,
"upper_bound":1000.0,
"gene_reaction_rule":""
},
{
"id":"cA_kin",
"name":"",
"metabolites":{
"ADP_c":1.0,
"AMP_c":1.0,
"ATP_c":-1.0,
"A_c":-1.0,
"H_c":1.0
},
"lower_bound":-1000.0,
"upper_bound":1000.0,
"gene_reaction_rule":""
},
{
"id":"cACCoA_carb",
"name":"",
"metabolites":{
"ACCoA_c":-1.0,
"ADP_c":1.0,
"ATP_c":-1.0,
"CO2_c":-1.0,
"H2O_c":-1.0,
"H_c":2.0,
"MACoA_c":1.0,
"Pi_c":1.0
},
"lower_bound":-1000.0,
"upper_bound":1000.0,
"gene_reaction_rule":""
},
{
"id":"cACCoA_syn",
"name":"",
"metabolites":{
"ACCoA_c":1.0,

```
"ACT_c":-1.0,
"AMP_c":1.0,
"ATP_c":-1.0,
"CoA_c":-1.0,
"H2O_c":-1.0,
"H_c":1.0,
"Pi_c":2.0
},
"lower_bound":-1000.0,
"upper_bound":1000.0,
"gene_reaction_rule":""
},
{
"id":"cACTAL_deh_(NAD)",
"name":"",
"metabolites":{
"ACTAL_c":-1.0,
"ACT_c":1.0,
"H2O_c":-1.0,
"H_c":2.0,
"NADH_c":1.0,
"NAD_c":-1.0
},
"lower_bound":0.0,
"upper_bound":1000.0,
"gene_reaction_rule":""
},
{
"id":"cACTAL_deh_(NADP)",
"name":"",
"metabolites":{
"ACTAL_c":-1.0,
"ACT_c":1.0,
"H2O_c":-1.0,
"H_c":2.0,
"NADPH_c":1.0,
"NADP_c":-1.0
},
"lower_bound":0.0,
"upper_bound":1000.0,
"gene_reaction_rule":""
},
{
"id":"cACT_t_mACT",
"name":"",
"metabolites":{
"ACT_c":-1.0,
"ACT_m":1.0,
"H_c":-1.0,
"H_m":1.0
},
"lower_bound":-1000.0,
"upper_bound":1000.0,
"gene_reaction_rule":""
```

},
{
"id":"cACTAL_t_mACTAL",
"name":"",
"metabolites":{
"ACTAL_c":-1.0,
"ACTAL_m":1.0
},
"lower_bound":-1000.0,
"upper_bound":1000.0,
"gene_reaction_rule":""
},
{
"id":"cAICAR_syn",
"name":"",
"metabolites":{
"ADP_c":4.0,
"AICAR_c":1.0,
"ASP_c":-1.0,
"ATP_c":-4.0,
"CO2_c":-1.0,
"FTHF_c":-1.0,
"FUM_c":1.0,
"GLM_c":2.0,
"GLN_c":-2.0,
"GLY_c":-1.0,
"H2O_c":-2.0,
"H_c":8.0,
"PPi_c":1.0,
"PRPP_c":-1.0,
"Pi_c":4.0,
"THF_c":1.0
},
"lower_bound":-1000.0,
"upper_bound":1000.0,
"gene_reaction_rule":""
},
{
"id":"cALA_N-trans",
"name":"",
"metabolites":{
"ALA_c":1.0,
"GLM_c":-1.0,
"OGL_c":1.0,
"PYR_c":-1.0
},
"lower_bound":-1000.0,
"upper_bound":1000.0,
"gene_reaction_rule":""
},
{
"id":"cAMP_kin",
"name":"",
"metabolites":{

"ADP_c":2.0,
"AMP_c":-1.0,
"ATP_c":-1.0
},
"lower_bound":-1000.0,
"upper_bound":1000.0,
"gene_reaction_rule":""
},
{
"id":"cAMP_syn",
"name":"",
"metabolites":{
"ADP_c":1.0,
"AMP_c":1.0,
"ASP_c":-1.0,
"ATP_c":-1.0,
"FUM_c":1.0,
"H_c":2.0,
"IMP_c":-1.0,
"Pi_c":1.0
},
"lower_bound":-1000.0,
"upper_bound":1000.0,
"gene_reaction_rule":""
},
{
"id":"cARG_syn",
"name":"",
"metabolites":{
"AMP_c":1.0,
"ARG_c":1.0,
"ASP_c":-1.0,
"ATP_c":-1.0,
"CARP_c":-1.0,
"FUM_c":1.0,
"H_c":3.0,
"ORN_c":-1.0,
"PPi_c":1.0,
"Pi_c":1.0
},
"lower_bound":-1000.0,
"upper_bound":1000.0,
"gene_reaction_rule":""
},
{
"id":"cASN_syn",
"name":"",
"metabolites":{
"ADP_c":1.0,
"ASN_c":1.0,
"ASP_c":-1.0,
"ATP_c":-1.0,
"GLM_c":1.0,
"GLN_c":-1.0,

"H2O_c":-1.0,
"H_c":1.0,
"Pi_c":1.0
},
"lower_bound":-1000.0,
"upper_bound":1000.0,
"gene_reaction_rule":""
},
{
"id":"cASP_kin",
"name":"",
"metabolites":{
"ADP_c":1.0,
"ASP_c":-1.0,
"ATP_c":-1.0,
"HSER_c":1.0,
"H_c":-2.0,
"NADPH_c":-2.0,
"NADP_c":2.0,
"Pi_c":1.0
},
"lower_bound":-1000.0,
"upper_bound":1000.0,
"gene_reaction_rule":""
},
{
"id":"cASP_N-trans",
"name":"",
"metabolites":{
"ASP_c":1.0,
"GLM_c":-1.0,
"OGL_c":1.0,
"OXACT_c":-1.0
},
"lower_bound":-1000.0,
"upper_bound":1000.0,
"gene_reaction_rule":""
},
{
"id":"cAvAA_form",
"name":"",
"metabolites":{
"ALA_c":-0.9769,
"ARG_c":-0.3856,
"ASN_c":-0.4085,
"ASP_c":-0.5199,
"CYS_c":-0.01387,
"GLM_c":-1.021,
"GLN_c":-0.5262,
"GLY_c":-0.8892,
"HIS_c":-0.1926,
"ILE_c":-0.5894,
"LEU_c":-0.8008,
"LYS_c":-0.657,

"MET_c":-0.1138,
"ORN_c":-0.02382,
"PHE_c":-0.3758,
"PRO_c":-0.4224,
"SER_c":-0.5328,
"THR_c":-0.5566,
"TRP_c":-0.06492,
"TYR_c":-0.196,
"VAL_c":-0.7326,
"avAA_c":10.0
},
"lower_bound":-1000.0,
"upper_bound":1000.0,
"gene_reaction_rule":""
},
{
"id":"cAvFA_form",
"name":"",
"metabolites":{
"OLE-CoA_c":-1.7,
"PLLM-CoA_c":-4.4,
"PLM-CoA_c":-1.4,
"STE-CoA_c":-1.0,
"avFA-CoA_c":8.5
},
"lower_bound":-1000.0,
"upper_bound":1000.0,
"gene_reaction_rule":""
},
{
"id":"cAvPL_form",
"name":"",
"metabolites":{
"PHD-CHO_c":-11.0,
"PHD-ETA_c":-4.0,
"PHD-SER_c":-3.0,
"avPL_c":18.0
},
"lower_bound":-1000.0,
"upper_bound":1000.0,
"gene_reaction_rule":""
},
{
"id":"cCARBHYD_syn",
"name":"",
"metabolites":{
"ADP_c":1.0,
"ATP_c":-1.0,
"CARBHYD_c":6.0,
"G6P_c":-1.0,
"H2O_c":-1.0,
"H_c":1.0,
"Pi_c":2.0
},

"lower_bound":-1000.0,
"upper_bound":1000.0,
"gene_reaction_rule":""
},
{
"id":"cCARP_syn",
"name":"",
"metabolites":{
"ADP_c":2.0,
"ATP_c":-2.0,
"CARP_c":1.0,
"CO2_c":-1.0,
"GLM_c":1.0,
"GLN_c":-1.0,
"H2O_c":-2.0,
"H_c":3.0,
"Pi_c":1.0
},
"lower_bound":-1000.0,
"upper_bound":1000.0,
"gene_reaction_rule":""
},
{
"id":"cCDP_kin",
"name":"",
"metabolites":{
"ADP_c":1.0,
"ATP_c":-1.0,
"CDP_c":-1.0,
"CTP_c":1.0
},
"lower_bound":-1000.0,
"upper_bound":1000.0,
"gene_reaction_rule":""
},
{
"id":"cCIT_syn",
"name":"",
"metabolites":{
"ACCoA_c":-1.0,
"CIT_c":1.0,
"CoA_c":1.0,
"H2O_c":-1.0,
"H_c":1.0,
"OXACT_c":-1.0
},
"lower_bound":0.0,
"upper_bound":1000.0,
"gene_reaction_rule":""
},
{
"id":"cCMP_kin",
"name":"",
"metabolites":{

"ADP_c":1.0,
"ATP_c":-1.0,
"CDP_c":1.0,
"CMP_c":-1.0
},
"lower_bound":-1000.0,
"upper_bound":1000.0,
"gene_reaction_rule":""
},
{
"id":"cCO2_t_mCO2",
"name":"",
"metabolites":{
"CO2_c":1.0,
"CO2_m":-1.0
},
"lower_bound":-1000.0,
"upper_bound":1000.0,
"gene_reaction_rule":""
},
{
"id":"cCTP_syn",
"name":"",
"metabolites":{
"ADP_c":1.0,
"ATP_c":-1.0,
"CTP_c":1.0,
"GLM_c":1.0,
"GLN_c":-1.0,
"H2O_c":-1.0,
"H_c":2.0,
"Pi_c":1.0,
"UTP_c":-1.0
},
"lower_bound":-1000.0,
"upper_bound":1000.0,
"gene_reaction_rule":""
},
{
"id":"cCYS_syn",
"name":"",
"metabolites":{
"CYS_c":1.0,
"HCYS_c":-1.0,
"H_c":-2.0,
"NH4_c":1.0,
"OBU_c":1.0,
"SER_c":-1.0
},
"lower_bound":-1000.0,
"upper_bound":1000.0,
"gene_reaction_rule":""
},
{

"id":"cDHF_red",
"name":"",
"metabolites":{
"DHF_c":-1.0,
"H_c":-1.0,
"NADPH_c":-1.0,
"NADP_c":1.0,
"THF_c":1.0
},
"lower_bound":-1000.0,
"upper_bound":1000.0,
"gene_reaction_rule":""
},
{
"id":"cDNA_poly",
"name":"",
"metabolites":{
"ATP_c":-0.3,
"CTP_c":-0.2,
"DHF_c":0.3,
"DNA_c":9.8,
"GTP_c":-0.2,
"H2O_c":1.0,
"H_c":-1.0,
"METHF_c":-0.3,
"NADPH_c":-1.0,
"NADP_c":1.0,
"PPi_c":1.0,
"UTP_c":-0.3
},
"lower_bound":-1000.0,
"upper_bound":1000.0,
"gene_reaction_rule":""
},
{
"id":"cEnol",
"name":"",
"metabolites":{
"2PG_c":-1.0,
"H2O_c":1.0,
"PEP_c":1.0
},
"lower_bound":-1000.0,
"upper_bound":1000.0,
"gene_reaction_rule":""
},
{
"id":"cH2O2_t_eH2O2",
"name":"",
"metabolites":{
"H2O2_c":-1.0,
"H2O2_e":1.0
},
"lower_bound":-1000.0,

"upper_bound":1000.0,
"gene_reaction_rule":""
},
{
"id":"cAOX",
"name":"",
"metabolites":{
"ACTAL_c":1.0,
"ETOH_c":-1.0,
"H2O2_c":1.0,
"O2_c":-1.0
},
"lower_bound":0.0,
"upper_bound":0.0,
"gene_reaction_rule":""
},
{
"id":"cETOH_deh",
"name":"",
"metabolites":{
"ACTAL_c":-1.0,
"ETOH_c":1.0,
"H_c":-1.0,
"NADH_c":-1.0,
"NAD_c":1.0
},
"lower_bound":-1000.0,
"upper_bound":1000.0,
"gene_reaction_rule":""
},
{
"id":"cETOH_t_mETOH",
"name":"",
"metabolites":{
"ETOH_c":-1.0,
"ETOH_m":1.0
},
"lower_bound":-1000.0,
"upper_bound":1000.0,
"gene_reaction_rule":""
},
{
"id":"cF16P_ald",
"name":"",
"metabolites":{
"DHAP_c":1.0,
"F16P_c":-1.0,
"GAP_c":1.0
},
"lower_bound":-1000.0,
"upper_bound":1000.0,
"gene_reaction_rule":""
},
{

"id":"cF16P_phos",
"name":"",
"metabolites":{
"F16P_c":-1.0,
"F6P_c":1.0,
"H2O_c":-1.0,
"Pi_c":1.0
},
"lower_bound":0.0,
"upper_bound":1000.0,
"gene_reaction_rule":""
},
{
"id":"cFAT_form",
"name":"",
"metabolites":{
"CoA_c":1.0,
"FAT_c":1.0,
"H2O_c":-1.0,
"PHD_c":-1.0,
"Pi_c":1.0,
"avFA-CoA_c":-1.0
},
"lower_bound":-1000.0,
"upper_bound":1000.0,
"gene_reaction_rule":""
},
{
"id":"cFUM_hy",
"name":"",
"metabolites":{
"FUM_c":-1.0,
"H2O_c":-1.0,
"MAL_c":1.0
},
"lower_bound":-1000.0,
"upper_bound":1000.0,
"gene_reaction_rule":""
},
{
"id":"cG6P_deh",
"name":"",
"metabolites":{
"CO2_c":1.0,
"G6P_c":-1.0,
"H2O_c":-1.0,
"H_c":2.0,
"NADPH_c":2.0,
"NADP_c":-2.0,
"RIBU5P_c":1.0
},
"lower_bound":0.0,
"upper_bound":1000.0,
"gene_reaction_rule":""

},
{
"id":"cG6P_iso",
"name":"",
"metabolites":{
"F6P_c":1.0,
"G6P_c":-1.0
},
"lower_bound":-1000.0,
"upper_bound":1000.0,
"gene_reaction_rule":""
},
{
"id":"cGAP_deh",
"name":"",
"metabolites":{
"13PG_c":1.0,
"GAP_c":-1.0,
"H_c":1.0,
"NADH_c":1.0,
"NAD_c":-1.0,
"Pi_c":-1.0
},
"lower_bound":-1000.0,
"upper_bound":1000.0,
"gene_reaction_rule":""
},
{
"id":"cGDP_kin",
"name":"",
"metabolites":{
"ADP_c":1.0,
"ATP_c":-1.0,
"GDP_c":-1.0,
"GTP_c":1.0
},
"lower_bound":-1000.0,
"upper_bound":1000.0,
"gene_reaction_rule":""
},
{
"id":"cGLM_deh",
"name":"",
"metabolites":{
"GLM_c":1.0,
"H2O_c":1.0,
"H_c":-1.0,
"NADPH_c":-1.0,
"NADP_c":1.0,
"NH4_c":-1.0,
"OGL_c":-1.0
},
"lower_bound":-1000.0,
"upper_bound":1000.0,

"gene_reaction_rule":""
},
{
"id":"cGLM_N-lig",
"name":"",
"metabolites":{
"ADP_c":1.0,
"ATP_c":-1.0,
"GLM_c":-1.0,
"GLN_c":1.0,
"H_c":1.0,
"NH4_c":-1.0,
"Pi_c":1.0
},
"lower_bound":-1000.0,
"upper_bound":1000.0,
"gene_reaction_rule":""
},
{
"id":"cGLY_transf",
"name":"",
"metabolites":{
"GLY_c":1.0,
"H2O_c":1.0,
"METHF_c":1.0,
"SER_c":-1.0,
"THF_c":-1.0
},
"lower_bound":-1000.0,
"upper_bound":1000.0,
"gene_reaction_rule":""
},
{
"id":"cGMP_kin",
"name":"",
"metabolites":{
"ADP_c":1.0,
"ATP_c":-1.0,
"GDP_c":1.0,
"GMP_c":-1.0
},
"lower_bound":-1000.0,
"upper_bound":1000.0,
"gene_reaction_rule":""
},
{
"id":"cGMP_syn",
"name":"",
"metabolites":{
"AMP_c":1.0,
"ATP_c":-1.0,
"GLM_c":1.0,
"GLN_c":-1.0,
"GMP_c":1.0,

"H2O_c":-2.0,
"H_c":4.0,
"IMP_c":-1.0,
"NADH_c":1.0,
"NAD_c":-1.0,
"PPi_c":1.0
},
"lower_bound":-1000.0,
"upper_bound":1000.0,
"gene_reaction_rule":""
},
{
"id":"cGOH3P_deh",
"name":"",
"metabolites":{
"DHAP_c":-1.0,
"GOH3P_c":1.0,
"H_c":-1.0,
"NADH_c":-1.0,
"NAD_c":1.0
},
"lower_bound":-1000.0,
"upper_bound":1000.0,
"gene_reaction_rule":""
},
{
"id":"cGOH3P_pho",
"name":"",
"metabolites":{
"GOH3P_c":-1.0,
"GOH_c":1.0,
"H2O_c":-1.0,
"Pi_c":1.0
},
"lower_bound":-1000.0,
"upper_bound":1000.0,
"gene_reaction_rule":""
},
{
"id":"cGOH3P_trans",
"name":"",
"metabolites":{
"CoA_c":2.0,
"GOH3P_c":-1.0,
"PHD_c":1.0,
"avFA-CoA_c":-2.0
},
"lower_bound":-1000.0,
"upper_bound":1000.0,
"gene_reaction_rule":""
},
{
"id":"cH2O_t_mH2O",
"name":"",

"metabolites":{
"H2O_c":1.0,
"H2O_m":-1.0
},
"lower_bound":-1000.0,
"upper_bound":1000.0,
"gene_reaction_rule":""
},
{
"id":"cHCIT_syn",
"name":"",
"metabolites":{
"ACCoA_c":-1.0,
"CoA_c":1.0,
"H2O_c":-1.0,
"HCIT_c":1.0,
"H_c":1.0,
"OGL_c":-1.0
},
"lower_bound":-1000.0,
"upper_bound":1000.0,
"gene_reaction_rule":""
},
{
"id":"cHCYS_syn",
"name":"",
"metabolites":{
"ACCoA_c":-1.0,
"ACT_c":1.0,
"CoA_c":1.0,
"H2S_c":-1.0,
"HCYS_c":1.0,
"HSER_c":-1.0,
"H_c":2.0
},
"lower_bound":-1000.0,
"upper_bound":1000.0,
"gene_reaction_rule":""
},
{
"id":"cHIS_syn",
"name":"",
"metabolites":{
"AICAR_c":1.0,
"ATP_c":-1.0,
"GLN_c":-1.0,
"H2O_c":-3.0,
"HIS_c":1.0,
"H_c":6.0,
"NADH_c":2.0,
"NAD_c":-2.0,
"OGL_c":1.0,
"PPi_c":2.0,
"PRPP_c":-1.0,

"Pi_c":1.0
},
"lower_bound":-1000.0,
"upper_bound":1000.0,
"gene_reaction_rule":""
},
{
"id":"cHX_trans",
"name":"",
"metabolites":{
"GLUC_c":1.0,
"GLUC_e":-1.0
},
"lower_bound":0.0,
"upper_bound":1000.0,
"gene_reaction_rule":""
},
{
"id":"cHX_kin",
"name":"",
"metabolites":{
"ADP_c":1.0,
"ATP_c":-1.0,
"G6P_c":1.0,
"GLUC_c":-1.0,
"H_c":1.0
},
"lower_bound":0.0,
"upper_bound":1000.0,
"gene_reaction_rule":""
},
{
"id":"cICIT_deh_NADP",
"name":"",
"metabolites":{
"CO2_c":1.0,
"ICIT_c":-1.0,
"NADPH_c":1.0,
"NADP_c":-1.0,
"OGL_c":1.0
},
"lower_bound":0.0,
"upper_bound":0.0,
"gene_reaction_rule":""
},
{
"id":"cICIT_lya",
"name":"",
"metabolites":{
"GLYO_c":1.0,
"ICIT_c":-1.0,
"SUC_c":1.0
},
"lower_bound":-1000.0,

"upper_bound":1000.0,
"gene_reaction_rule":""
},
{
"id":"cIMP_syn",
"name":"",
"metabolites":{
"AICAR_c":-1.0,
"FTHF_c":-1.0,
"H2O_c":1.0,
"IMP_c":1.0,
"THF_c":1.0
},
"lower_bound":-1000.0,
"upper_bound":1000.0,
"gene_reaction_rule":""
},
{
"id":"cIPM_syn",
"name":"",
"metabolites":{
"ACCoA_c":-1.0,
"CoA_c":1.0,
"H2O_c":-1.0,
"H_c":1.0,
"IPM_c":1.0,
"OIV_c":-1.0
},
"lower_bound":-1000.0,
"upper_bound":1000.0,
"gene_reaction_rule":""
},
{
"id":"cLEU_N-trans",
"name":"",
"metabolites":{
"CO2_c":1.0,
"GLM_c":-1.0,
"IPM_c":-1.0,
"LEU_c":1.0,
"NADH_c":1.0,
"NAD_c":-1.0,
"OGL_c":1.0
},
"lower_bound":-1000.0,
"upper_bound":1000.0,
"gene_reaction_rule":""
},
{
"id":"cLipid_form",
"name":"",
"metabolites":{
"FAT_c":-0.55,
"LIPID_c":47.22,

"avPL_c":-0.45
},
"lower_bound":-1000.0,
"upper_bound":1000.0,
"gene_reaction_rule":""
},
{
"id":"cLYS_syn",
"name":"",
"metabolites":{
"AMP_c":1.0,
"ATP_c":-1.0,
"GLM_c":-2.0,
"LYS_c":1.0,
"NADH_c":1.0,
"NADPH_c":-2.0,
"NADP_c":2.0,
"NAD_c":-1.0,
"OAD_c":-1.0,
"OGL_c":2.0,
"PPi_c":1.0
},
"lower_bound":-1000.0,
"upper_bound":1000.0,
"gene_reaction_rule":""
},
{
"id":"cMAL_deh",
"name":"",
"metabolites":{
"H_c":1.0,
"MAL_c":-1.0,
"NADH_c":1.0,
"NAD_c":-1.0,
"OXACT_c":1.0
},
"lower_bound":0.0,
"upper_bound":1000.0,
"gene_reaction_rule":""
},
{
"id":"cMAL_syn",
"name":"",
"metabolites":{
"ACCoA_c":-1.0,
"CoA_c":1.0,
"GLYO_c":-1.0,
"H2O_c":-1.0,
"H_c":1.0,
"MAL_c":1.0
},
"lower_bound":-1000.0,
"upper_bound":1000.0,
"gene_reaction_rule":""

},
{
"id":"cMET_Atrans",
"name":"",
"metabolites":{
"ATP_c":-1.0,
"H2O_c":-2.0,
"H_c":1.0,
"MET_c":-1.0,
"Pi_c":3.0,
"SAM_c":1.0
},
"lower_bound":-1000.0,
"upper_bound":1000.0,
"gene_reaction_rule":""
},
{
"id":"cMET_syn",
"name":"",
"metabolites":{
"HCYS_c":-1.0,
"H_c":-1.0,
"MET_c":1.0,
"MYTHF_c":-1.0,
"THF_c":1.0
},
"lower_bound":-1000.0,
"upper_bound":1000.0,
"gene_reaction_rule":""
},
{
"id":"cMETHF_deh",
"name":"",
"metabolites":{
"FTHF_c":-1.0,
"H2O_c":1.0,
"H_c":-1.0,
"METHF_c":1.0,
"NADPH_c":-1.0,
"NADP_c":1.0
},
"lower_bound":-1000.0,
"upper_bound":1000.0,
"gene_reaction_rule":""
},
{
"id":"cMETHF_red",
"name":"",
"metabolites":{
"H_c":-1.0,
"METHF_c":-1.0,
"MYTHF_c":1.0,
"NADPH_c":-1.0,
"NADP_c":1.0

},
"lower_bound":-1000.0,
"upper_bound":1000.0,
"gene_reaction_rule":""
},
{
"id":"cNADH_deh",
"name":"",
"metabolites":{
"H2O_m":1.0,
"H_c":5.0,
"H_m":-6.0,
"NADH_c":-1.0,
"NAD_c":1.0,
"O2_m":-0.5
},
"lower_bound":0.0,
"upper_bound":1000.0,
"gene_reaction_rule":""
},
{
"id":"cO2_t_mO2",
"name":"",
"metabolites":{
"O2_c":-1.0,
"O2_m":1.0
},
"lower_bound":-1000.0,
"upper_bound":1000.0,
"gene_reaction_rule":""
},
{
"id":"cPEP_ck",
"name":"",
"metabolites":{
"ADP_c":1.0,
"ATP_c":-1.0,
"CO2_c":1.0,
"OXACT_c":-1.0,
"PEP_c":1.0
},
"lower_bound":0.0,
"upper_bound":1000.0,
"gene_reaction_rule":""
},
{
"id":"cPF_kin",
"name":"",
"metabolites":{
"ADP_c":1.0,
"ATP_c":-1.0,
"F16P_c":1.0,
"F6P_c":-1.0,
"H_c":1.0

},
"lower_bound":0.0,
"upper_bound":1000.0,
"gene_reaction_rule":""
},
{
"id":"cPHD-C_trans",
"name":"",
"metabolites":{
"CMP-DGOH_c":1.0,
"CTP_c":-1.0,
"H2O_c":-1.0,
"PHD_c":-1.0,
"Pi_c":2.0
},
"lower_bound":-1000.0,
"upper_bound":1000.0,
"gene_reaction_rule":""
},
{
"id":"cPHD-EA_mtrans",
"name":"",
"metabolites":{
"H_c":3.0,
"PHD-CHO_c":1.0,
"PHD-ETA_c":-1.0,
"SAH_c":3.0,
"SAM_c":-3.0
},
"lower_bound":-1000.0,
"upper_bound":1000.0,
"gene_reaction_rule":""
},
{
"id":"cPHD-SER_dcarb",
"name":"",
"metabolites":{
"CO2_c":1.0,
"PHD-ETA_c":1.0,
"PHD-SER_c":-1.0
},
"lower_bound":-1000.0,
"upper_bound":1000.0,
"gene_reaction_rule":""
},
{
"id":"cPHD-SER_syn",
"name":"",
"metabolites":{
"CMP-DGOH_c":-1.0,
"CMP_c":1.0,
"PHD-SER_c":1.0,
"SER_c":-1.0
},

"lower_bound":-1000.0,
"upper_bound":1000.0,
"gene_reaction_rule":""
},
{
"id":"cPHE_syn",
"name":"",
"metabolites":{
"CHO_c":-1.0,
"CO2_c":1.0,
"GLM_c":-1.0,
"H2O_c":1.0,
"H_c":-1.0,
"OGL_c":1.0,
"PHE_c":1.0
},
"lower_bound":-1000.0,
"upper_bound":1000.0,
"gene_reaction_rule":""
},
{
"id":"cPLM_desat",
"name":"",
"metabolites":{
"H2O_c":2.0,
"H_c":-1.0,
"NADPH_c":-1.0,
"NADP_c":1.0,
"O2_c":-1.0,
"PLLM-CoA_c":1.0,
"PLM-CoA_c":-1.0
},
"lower_bound":-1000.0,
"upper_bound":1000.0,
"gene_reaction_rule":""
},
{
"id":"cPLM_lig",
"name":"",
"metabolites":{
"AMP_c":1.0,
"ATP_c":-1.0,
"CoA_c":-1.0,
"H2O_c":-1.0,
"H_c":1.0,
"PLM-CoA_c":1.0,
"PLM_c":-1.0,
"Pi_c":2.0
},
"lower_bound":-1000.0,
"upper_bound":1000.0,
"gene_reaction_rule":""
},
{

"id":"cPLM_syn",
"name":"",
"metabolites":{
"ACCoA_c":-1.0,
"CO2_c":7.0,
"CoA_c":8.0,
"H2O_c":6.0,
"H_c":-20.0,
"MACoA_c":-7.0,
"NADPH_c":-14.0,
"NADP_c":14.0,
"PLM_c":1.0
},
"lower_bound":-1000.0,
"upper_bound":1000.0,
"gene_reaction_rule":""
},
{
"id":"cPPi_ase",
"name":"",
"metabolites":{
"H2O_c":-1.0,
"PPi_c":-1.0,
"Pi_c":2.0
},
"lower_bound":-1000.0,
"upper_bound":1000.0,
"gene_reaction_rule":""
},
{
"id":"cPRO_deh",
"name":"",
"metabolites":{
"ADP_c":1.0,
"ATP_c":-1.0,
"GLM_c":-1.0,
"H2O_c":1.0,
"H_c":-2.0,
"NADPH_c":-2.0,
"NADP_c":2.0,
"PRO_c":1.0,
"Pi_c":1.0
},
"lower_bound":-1000.0,
"upper_bound":1000.0,
"gene_reaction_rule":""
},
{
"id":"cPROT_poly",
"name":"",
"metabolites":{
"ADP_c":2.0,
"AMP_c":1.0,
"ATP_c":-3.0,

"H2O_c":-2.0,
"H_c":4.0,
"PPi_c":1.0,
"PROT_c":4.81,
"Pi_c":2.0,
"avAA_c":-1.0
},
"lower_bound":-1000.0,
"upper_bound":1000.0,
"gene_reaction_rule":""
},
{
"id":"cPRPP_syn",
"name":"",
"metabolites":{
"AMP_c":1.0,
"ATP_c":-1.0,
"H_c":1.0,
"PRPP_c":1.0,
"RIBU5P_c":-1.0
},
"lower_bound":-1000.0,
"upper_bound":1000.0,
"gene_reaction_rule":""
},
{
"id":"cPYR_carb",
"name":"",
"metabolites":{
"ADP_c":1.0,
"ATP_c":-1.0,
"CO2_c":-1.0,
"H2O_c":-1.0,
"H_c":2.0,
"OXACT_c":1.0,
"PYR_c":-1.0,
"Pi_c":1.0
},
"lower_bound":0.0,
"upper_bound":1000.0,
"gene_reaction_rule":""
},
{
"id":"cPYR_dec",
"name":"",
"metabolites":{
"ACTAL_c":1.0,
"CO2_c":1.0,
"H_c":-1.0,
"PYR_c":-1.0
},
"lower_bound":0.0,
"upper_bound":1000.0,
"gene_reaction_rule":""

},
{
"id":"cPYR_kin",
"name":"",
"metabolites":{
"ADP_c":-1.0,
"ATP_c":1.0,
"H_c":-1.0,
"PEP_c":-1.0,
"PYR_c":1.0
},
"lower_bound":0.0,
"upper_bound":1000.0,
"gene_reaction_rule":""
},
{
"id":"cRIBU_iso",
"name":"",
"metabolites":{
"RIB5P_c":1.0,
"RIBU5P_c":-1.0
},
"lower_bound":-1000.0,
"upper_bound":1000.0,
"gene_reaction_rule":""
},
{
"id":"cRIBUP_epi",
"name":"",
"metabolites":{
"RIBU5P_c":-1.0,
"XYL5P_c":1.0
},
"lower_bound":-1000.0,
"upper_bound":1000.0,
"gene_reaction_rule":""
},
{
"id":"cRNA_syn",
"name":"",
"metabolites":{
"ATP_c":-0.3,
"CTP_c":-0.2,
"GTP_c":-0.2,
"PPi_c":1.0,
"RNA_c":9.5,
"UTP_c":-0.3
},
"lower_bound":-1000.0,
"upper_bound":1000.0,
"gene_reaction_rule":""
},
{
"id":"cSAH_hyd",

"name":"",
"metabolites":{
"A_c":1.0,
"H2O_c":-1.0,
"HCYS_c":1.0,
"H_c":1.0,
"SAH_c":-1.0
},
"lower_bound":-1000.0,
"upper_bound":1000.0,
"gene_reaction_rule":""
},
{
"id":"cSER_syn",
"name":"",
"metabolites":{
"3PG_c":-1.0,
"GLM_c":-1.0,
"H2O_c":-1.0,
"H_c":1.0,
"NADH_c":1.0,
"NAD_c":-1.0,
"OGL_c":1.0,
"Pi_c":1.0,
"SER_c":1.0
},
"lower_bound":-1000.0,
"upper_bound":1000.0,
"gene_reaction_rule":""
},
{
"id":"cSHI_path",
"name":"",
"metabolites":{
"ADP_c":1.0,
"ATP_c":-1.0,
"CHO_c":1.0,
"E4P_c":-1.0,
"NADPH_c":-1.0,
"NADP_c":1.0,
"PEP_c":-2.0,
"Pi_c":4.0
},
"lower_bound":-1000.0,
"upper_bound":1000.0,
"gene_reaction_rule":""
},
{
"id":"cSO4_ass",
"name":"",
"metabolites":{
"ADP_c":1.0,
"AMP_c":1.0,
"ATP_c":-2.0,

"H2O_c":2.0,
"H2S_c":1.0,
"H_c":-3.0,
"NADPH_c":-4.0,
"NADP_c":4.0,
"PPi_c":1.0,
"Pi_c":1.0,
"SO4_c":-1.0
},
"lower_bound":-1000.0,
"upper_bound":1000.0,
"gene_reaction_rule":""
},
{
"id":"cSTE_desat",
"name":"",
"metabolites":{
"H2O_c":2.0,
"H_c":-1.0,
"NADPH_c":-1.0,
"NADP_c":1.0,
"O2_c":-1.0,
"OLE-CoA_c":1.0,
"STE-CoA_c":-1.0
},
"lower_bound":-1000.0,
"upper_bound":1000.0,
"gene_reaction_rule":""
},
{
"id":"cSTE_ligase",
"name":"",
"metabolites":{
"AMP_c":1.0,
"ATP_c":-1.0,
"CoA_c":-1.0,
"H2O_c":-1.0,
"H_c":1.0,
"Pi_c":2.0,
"STE-CoA_c":1.0,
"STE_c":-1.0
},
"lower_bound":-1000.0,
"upper_bound":1000.0,
"gene_reaction_rule":""
},
{
"id":"cSTE_syn",
"name":"",
"metabolites":{
"ACCoA_c":-1.0,
"CO2_c":8.0,
"CoA_c":9.0,
"H2O_c":7.0,

"H_c":-23.0,
"MACoA_c":-8.0,
"NADPH_c":-16.0,
"NADP_c":16.0,
"STE_c":1.0
},
"lower_bound":-1000.0,
"upper_bound":1000.0,
"gene_reaction_rule":""
},
{
"id":"cTA1",
"name":"",
"metabolites":{
"E4P_c":1.0,
"F6P_c":1.0,
"GAP_c":-1.0,
"SED7P_c":-1.0
},
"lower_bound":-1000.0,
"upper_bound":1000.0,
"gene_reaction_rule":""
},
{
"id":"cTHR_ald",
"name":"",
"metabolites":{
"ACTAL_c":1.0,
"GLY_c":1.0,
"THR_c":-1.0
},
"lower_bound":-1000.0,
"upper_bound":1000.0,
"gene_reaction_rule":""
},
{
"id":"cTHR_syn",
"name":"",
"metabolites":{
"ADP_c":1.0,
"ATP_c":-1.0,
"H2O_c":-1.0,
"HSER_c":-1.0,
"H_c":1.0,
"Pi_c":1.0,
"THR_c":1.0
},
"lower_bound":-1000.0,
"upper_bound":1000.0,
"gene_reaction_rule":""
},
{
"id":"cTK1",
"name":"",

"metabolites":{
"GAP_c":1.0,
"RIB5P_c":-1.0,
"SED7P_c":1.0,
"XYL5P_c":-1.0
},
"lower_bound":-1000.0,
"upper_bound":1000.0,
"gene_reaction_rule":""
},
{
"id":"cTK2",
"name":"",
"metabolites":{
"E4P_c":-1.0,
"F6P_c":1.0,
"GAP_c":1.0,
"XYL5P_c":-1.0
},
"lower_bound":-1000.0,
"upper_bound":1000.0,
"gene_reaction_rule":""
},
{
"id":"cTP_iso",
"name":"",
"metabolites":{
"DHAP_c":-1.0,
"GAP_c":1.0
},
"lower_bound":-1000.0,
"upper_bound":1000.0,
"gene_reaction_rule":""
},
{
"id":"CTP1m_car",
"name":"",
"metabolites":{
"CIT_c":-1.0,
"CIT_m":1.0,
"H_c":-1.0,
"H_m":1.0,
"OXACT_c":1.0,
"OXACT_m":-1.0
},
"lower_bound":-1000.0,
"upper_bound":1000.0,
"gene_reaction_rule":""
},
{
"id":"CTP2m_car",
"name":"",
"metabolites":{
"H_c":1.0,

"H_m":-1.0,
"ICIT_c":1.0,
"ICIT_m":-1.0,
"OXACT_c":-1.0,
"OXACT_m":1.0
},
"lower_bound":-1000.0,
"upper_bound":1000.0,
"gene_reaction_rule":""
},
{
"id":"cTRP_syn",
"name":"",
"metabolites":{
"CHO_c":-1.0,
"CO2_c":1.0,
"GAP_c":1.0,
"GLM_c":1.0,
"GLN_c":-1.0,
"H2O_c":1.0,
"H_c":1.0,
"PRPP_c":-1.0,
"PYR_c":1.0,
"Pi_c":2.0,
"SER_c":-1.0,
"TRP_c":1.0
},
"lower_bound":-1000.0,
"upper_bound":1000.0,
"gene_reaction_rule":""
},
{
"id":"cTYR_syn",
"name":"",
"metabolites":{
"CHO_c":-1.0,
"CO2_c":1.0,
"GLM_c":-1.0,
"NADPH_c":1.0,
"NADP_c":-1.0,
"OGL_c":1.0,
"TYR_c":1.0
},
"lower_bound":-1000.0,
"upper_bound":1000.0,
"gene_reaction_rule":""
},
{
"id":"cUDP_kin",
"name":"",
"metabolites":{
"ADP_c":1.0,
"ATP_c":-1.0,
"UDP_c":-1.0,

"UTP_c":1.0
},
"lower_bound":-1000.0,
"upper_bound":1000.0,
"gene_reaction_rule":""
},
{
"id":"cUMP_kin",
"name":"",
"metabolites":{
"ADP_c":1.0,
"ATP_c":-1.0,
"UDP_c":1.0,
"UMP_c":-1.0
},
"lower_bound":-1000.0,
"upper_bound":1000.0,
"gene_reaction_rule":""
},
{
"id":"cUMP_syn",
"name":"",
"metabolites":{
"ASP_c":-1.0,
"CARP_c":-1.0,
"CO2_c":1.0,
"H2O_c":2.0,
"O2_c":-0.5,
"PPi_c":1.0,
"PRPP_c":-1.0,
"Pi_c":1.0,
"UMP_c":1.0
},
"lower_bound":-1000.0,
"upper_bound":1000.0,
"gene_reaction_rule":""
},
{
"id":"cVAL_N-trans",
"name":"",
"metabolites":{
"GLM_c":-1.0,
"OGL_c":1.0,
"OIV_c":-1.0,
"VAL_c":1.0
},
"lower_bound":-1000.0,
"upper_bound":1000.0,
"gene_reaction_rule":""
},
{
"id":"eACT_trans",
"name":"",
"metabolites":{

"ACT_c":1.0,
"ACT_e":-1.0,
"H_c":1.0,
"H_e":-1.0
},
"lower_bound":-1000.0,
"upper_bound":1000.0,
"gene_reaction_rule":""
},
{
"id":"eATPase",
"name":"",
"metabolites":{
"ADP_c":1.0,
"ATP_c":-1.0,
"H2O_c":-1.0,
"H_e":1.0,
"Pi_c":1.0
},
"lower_bound":-1000.0,
"upper_bound":1000.0,
"gene_reaction_rule":""
},
{
"id":"eCO2_t_cCO2",
"name":"",
"metabolites":{
"CO2_c":-1.0,
"CO2_e":1.0
},
"lower_bound":-1000.0,
"upper_bound":1000.0,
"gene_reaction_rule":""
},
{
"id":"eETOH_trans",
"name":"",
"metabolites":{
"ETOH_c":-1.0,
"ETOH_e":1.0
},
"lower_bound":-1000.0,
"upper_bound":1000.0,
"gene_reaction_rule":""
},
{
"id":"eGOH_trans",
"name":"",
"metabolites":{
"GOH_c":-1.0,
"GOH_e":1.0
},
"lower_bound":-1000.0,
"upper_bound":1000.0,

"gene_reaction_rule":""
},
{
"id":"eH2O_t_cH2O",
"name":"",
"metabolites":{
"H2O_c":-1.0,
"H2O_e":1.0
},
"lower_bound":-1000.0,
"upper_bound":1000.0,
"gene_reaction_rule":""
},
{
"id":"eNH4_trans",
"name":"",
"metabolites":{
"NH4_c":1.0,
"NH4_e":-1.0
},
"lower_bound":-1000.0,
"upper_bound":1000.0,
"gene_reaction_rule":""
},
{
"id":"eO2_t_cO2",
"name":"",
"metabolites":{
"O2_c":1.0,
"O2_e":-1.0
},
"lower_bound":-1000.0,
"upper_bound":1000.0,
"gene_reaction_rule":""
},
{
"id":"ePi_trans",
"name":"",
"metabolites":{
"H_c":2.0,
"H_e":-2.0,
"Pi_c":1.0,
"Pi_e":-1.0
},
"lower_bound":-1000.0,
"upper_bound":1000.0,
"gene_reaction_rule":""
},
{
"id":"eSO4_trans",
"name":"",
"metabolites":{
"H_c":3.0,
"H_e":-3.0,

"SO4_c":1.0,
"SO4_e":-1.0
},
"lower_bound":-1000.0,
"upper_bound":1000.0,
"gene_reaction_rule":""
},
{
"id":"HCITm_car",
"name":"",
"metabolites":{
"HCIT_c":-1.0,
"HCIT_m":1.0,
"H_c":-4.0,
"H_m":4.0
},
"lower_bound":-1000.0,
"upper_bound":1000.0,
"gene_reaction_rule":""
},
{
"id":"ILEm_car",
"name":"",
"metabolites":{
"H_c":-1.0,
"H_m":1.0,
"ILE_c":1.0,
"ILE_m":-1.0
},
"lower_bound":-1000.0,
"upper_bound":1000.0,
"gene_reaction_rule":""
},
{
"id":"mACON_1",
"name":"",
"metabolites":{
"ACO_m":1.0,
"CIT_m":-1.0,
"H2O_m":1.0
},
"lower_bound":-1000.0,
"upper_bound":1000.0,
"gene_reaction_rule":""
},
{
"id":"mACON_2",
"name":"",
"metabolites":{
"ACO_m":-1.0,
"H2O_m":-1.0,
"ICIT_m":1.0
},
"lower_bound":-1000.0,

"upper_bound":1000.0,
"gene_reaction_rule":""
},
{
"id":"mACTAL_deh_(NAD)",
"name":"",
"metabolites":{
"ACTAL_m":-1.0,
"ACT_m":1.0,
"H2O_m":-1.0,
"H_m":2.0,
"NADH_m":1.0,
"NAD_m":-1.0
},
"lower_bound":0.0,
"upper_bound":0.0,
"gene_reaction_rule":""
},
{
"id":"mACTAL_deh_(NADP)",
"name":"",
"metabolites":{
"ACTAL_m":-1.0,
"ACT_m":1.0,
"H2O_m":-1.0,
"H_m":2.0,
"NADPH_m":1.0,
"NADP_m":-1.0
},
"lower_bound":0.0,
"upper_bound":0.0,
"gene_reaction_rule":""
},
{
"id":"mATPase",
"name":"",
"metabolites":{
"ADP_m":-3.0,
"ATP_m":3.0,
"H2O_m":3.0,
"H_c":-10.0,
"H_m":7.0,
"Pi_m":-3.0
},
"lower_bound":-1000.0,
"upper_bound":1000.0,
"gene_reaction_rule":""
},
{
"id":"mCIT_syn",
"name":"",
"metabolites":{
"ACCoA_m":-1.0,
"CIT_m":1.0,

"CoA_m":1.0,
"H2O_m":-1.0,
"H_m":1.0,
"OXACT_m":-1.0
},
"lower_bound":0.0,
"upper_bound":1000.0,
"gene_reaction_rule":""
},
{
"id":"mETOH_deh",
"name":"",
"metabolites":{
"ACTAL_m":-1.0,
"ETOH_m":1.0,
"H_m":-1.0,
"NADH_m":-1.0,
"NAD_m":1.0
},
"lower_bound":0.0,
"upper_bound":0.0,
"gene_reaction_rule":""
},
{
"id":"mFUM_hy",
"name":"",
"metabolites":{
"FUM_m":-1.0,
"H2O_m":-1.0,
"MAL_m":1.0
},
"lower_bound":-1000.0,
"upper_bound":1000.0,
"gene_reaction_rule":""
},
{
"id":"mICIT_deh_NAD",
"name":"",
"metabolites":{
"CO2_m":1.0,
"ICIT_m":-1.0,
"NADH_m":1.0,
"NAD_m":-1.0,
"OGL_m":1.0
},
"lower_bound":0.0,
"upper_bound":1000.0,
"gene_reaction_rule":""
},
{
"id":"mICIT_deh_NADP",
"name":"",
"metabolites":{
"CO2_m":1.0,

"ICIT_m":-1.0,
"NADPH_m":1.0,
"NADP_m":-1.0,
"OGL_m":1.0
},
"lower_bound":0.0,
"upper_bound":1000.0,
"gene_reaction_rule":""
},
{
"id":"mILE_N-trans",
"name":"",
"metabolites":{
"CO2_m":1.0,
"GLM_m":-1.0,
"H2O_m":1.0,
"H_m":-1.0,
"ILE_m":1.0,
"NADPH_m":-1.0,
"NADP_m":1.0,
"OBU_m":-1.0,
"OGL_m":1.0,
"PYR_m":-1.0
},
"lower_bound":-1000.0,
"upper_bound":1000.0,
"gene_reaction_rule":""
},
{
"id":"mMAL_deh",
"name":"",
"metabolites":{
"H_m":1.0,
"MAL_m":-1.0,
"NADH_m":1.0,
"NAD_m":-1.0,
"OXACT_m":1.0
},
"lower_bound":-1000.0,
"upper_bound":1000.0,
"gene_reaction_rule":""
},
{
"id":"mNADH_deh_CI",
"name":"",
"metabolites":{
"H2O_m":1.0,
"H_c":10.0,
"H_m":-11.0,
"NADH_m":-1.0,
"NAD_m":1.0,
"O2_m":-0.5
},
"lower_bound":0.0,

"upper_bound":0.0,
"gene_reaction_rule":""
},
{
"id":"mNADH_deh_ND",
"name":"",
"metabolites":{
"H2O_m":1.0,
"H_c":6.0,
"H_m":-7.0,
"NADH_m":-1.0,
"NAD_m":1.0,
"O2_m":-0.5
},
"lower_bound":0.0,
"upper_bound":1000.0,
"gene_reaction_rule":""
},
{
"id":"mOAD_syn",
"name":"",
"metabolites":{
"CO2_m":1.0,
"HCIT_m":-1.0,
"NADH_m":1.0,
"NAD_m":-1.0,
"OAD_m":1.0
},
"lower_bound":-1000.0,
"upper_bound":1000.0,
"gene_reaction_rule":""
},
{
"id":"mOGL_deh",
"name":"",
"metabolites":{
"CO2_m":1.0,
"CoA_m":-1.0,
"NADH_m":1.0,
"NAD_m":-1.0,
"OGL_m":-1.0,
"SUCCOA_m":1.0
},
"lower_bound":-1000.0,
"upper_bound":1000.0,
"gene_reaction_rule":""
},
{
"id":"mOIV_syn",
"name":"",
"metabolites":{
"CO2_m":1.0,
"H2O_m":1.0,
"H_m":-2.0,

"NADPH_m":-1.0,
"NADP_m":1.0,
"OIV_m":1.0,
"PYR_m":-2.0
},
"lower_bound":-1000.0,
"upper_bound":1000.0,
"gene_reaction_rule":""
},
{
"id":"mORN_syn",
"name":"",
"metabolites":{
"ADP_m":1.0,
"ATP_m":-1.0,
"GLM_m":-2.0,
"H_m":-1.0,
"NADPH_m":-1.0,
"NADP_m":1.0,
"OGL_m":1.0,
"ORN_m":1.0,
"Pi_m":1.0
},
"lower_bound":-1000.0,
"upper_bound":1000.0,
"gene_reaction_rule":""
},
{
"id":"mPYR_deh",
"name":"",
"metabolites":{
"ACCoA_m":1.0,
"CO2_m":1.0,
"CoA_m":-1.0,
"NADH_m":1.0,
"NAD_m":-1.0,
"PYR_m":-1.0
},
"lower_bound":0.0,
"upper_bound":1000.0,
"gene_reaction_rule":""
},
{
"id":"mQH2_deh",
"name":"",
"metabolites":{
"H2O_m":1.0,
"H_c":6.0,
"H_m":-6.0,
"O2_m":-0.5,
"QH2_m":-1.0,
"Q_m":1.0
},
"lower_bound":0.0,

"upper_bound":1000.0,
"gene_reaction_rule":""
},
{
"id":"mSUC_deh",
"name":"",
"metabolites":{
"FUM_m":1.0,
"QH2_m":1.0,
"Q_m":-1.0,
"SUC_m":-1.0
},
"lower_bound":-1000.0,
"upper_bound":1000.0,
"gene_reaction_rule":""
},
{
"id":"mSUCCoA_syn",
"name":"",
"metabolites":{
"ADP_m":-1.0,
"ATP_m":1.0,
"CoA_m":1.0,
"Pi_m":-1.0,
"SUCCOA_m":-1.0,
"SUC_m":1.0
},
"lower_bound":-1000.0,
"upper_bound":1000.0,
"gene_reaction_rule":""
},
{
"id":"mTHR_deh",
"name":"",
"metabolites":{
"H_m":-1.0,
"NH4_m":1.0,
"OBU_m":1.0,
"THR_m":-1.0
},
"lower_bound":-1000.0,
"upper_bound":1000.0,
"gene_reaction_rule":""
},
{
"id":"mVAL_N-trans",
"name":"",
"metabolites":{
"GLM_m":-1.0,
"OGL_m":1.0,
"OIV_m":-1.0,
"VAL_m":1.0
},
"lower_bound":-1000.0,

"upper_bound":1000.0,
"gene_reaction_rule":""
},
{
"id":"NH4m_car",
"name":"",
"metabolites":{
"H_c":-1.0,
"H_m":1.0,
"NH4_c":1.0,
"NH4_m":-1.0
},
"lower_bound":-1000.0,
"upper_bound":1000.0,
"gene_reaction_rule":""
},
{
"id":"OACm_car",
"name":"",
"metabolites":{
"MAL_c":-1.0,
"MAL_m":1.0,
"OXACT_c":1.0,
"OXACT_m":-1.0
},
"lower_bound":-1000.0,
"upper_bound":1000.0,
"gene_reaction_rule":""
},
{
"id":"OBUm_car",
"name":"",
"metabolites":{
"H_c":-1.0,
"H_m":1.0,
"OBU_c":-1.0,
"OBU_m":1.0
},
"lower_bound":-1000.0,
"upper_bound":1000.0,
"gene_reaction_rule":""
},
{
"id":"ODC1m_car",
"name":"",
"metabolites":{
"MAL_c":-1.0,
"MAL_m":1.0,
"OGL_c":1.0,
"OGL_m":-1.0
},
"lower_bound":-1000.0,
"upper_bound":1000.0,
"gene_reaction_rule":""

},
{
"id":"ODC2m_car",
"name":"",
"metabolites":{
"OAD_c":1.0,
"OAD_m":-1.0,
"OGL_c":-1.0,
"OGL_m":1.0
},
"lower_bound":-1000.0,
"upper_bound":1000.0,
"gene_reaction_rule":""
},
{
"id":"OIVm_car",
"name":"",
"metabolites":{
"OIV_c":1.0,
"OIV_m":-1.0
},
"lower_bound":-1000.0,
"upper_bound":1000.0,
"gene_reaction_rule":""
},
{
"id":"ORNm_car",
"name":"",
"metabolites":{
"H_c":-1.0,
"H_m":1.0,
"ORN_c":1.0,
"ORN_m":-1.0
},
"lower_bound":-1000.0,
"upper_bound":1000.0,
"gene_reaction_rule":""
},
{
"id":"OXACTm_ex",
"name":"",
"metabolites":{
"H_c":1.0,
"H_m":-1.0,
"OXACT_c":1.0,
"OXACT_m":-1.0
},
"lower_bound":-1000.0,
"upper_bound":1000.0,
"gene_reaction_rule":""
},
{
"id":"Pi_m_car",
"name":"",

"metabolites":{
"H_c":-2.0,
"H_m":2.0,
"Pi_c":-1.0,
"Pi_m":1.0
},
"lower_bound":-1000.0,
"upper_bound":1000.0,
"gene_reaction_rule":""
},
{
"id":"PYRm_car",
"name":"",
"metabolites":{
"H_c":-2.0,
"H_m":2.0,
"PYR_c":-1.0,
"PYR_m":1.0
},
"lower_bound":-1000.0,
"upper_bound":1000.0,
"gene_reaction_rule":""
},
{
"id":"SFC1m_car",
"name":"",
"metabolites":{
"FUM_c":1.0,
"FUM_m":-1.0,
"SUC_c":-1.0,
"SUC_m":1.0
},
"lower_bound":-1000.0,
"upper_bound":1000.0,
"gene_reaction_rule":""
},
{
"id":"THRm_car",
"name":"",
"metabolites":{
"H_c":-1.0,
"H_m":1.0,
"THR_c":-1.0,
"THR_m":1.0
},
"lower_bound":-1000.0,
"upper_bound":1000.0,
"gene_reaction_rule":""
},
{
"id":"VALm_imp",
"name":"",
"metabolites":{
"H_c":-1.0,

"H_m":1.0,
"VAL_c":-1.0,
"VAL_m":1.0
},
"lower_bound":-1000.0,
"upper_bound":1000.0,
"gene_reaction_rule":""
},
{
"id":"EX_H",
"name":"",
"metabolites":{
"H_e":-1.0
},
"lower_bound":-1000.0,
"upper_bound":1000.0,
"gene_reaction_rule":""
},
{
"id":"EX_H2O",
"name":"",
"metabolites":{
"H2O_e":-1.0
},
"lower_bound":-1000.0,
"upper_bound":1000.0,
"gene_reaction_rule":""
},
{
"id":"EX_NH4",
"name":"",
"metabolites":{
"NH4_e":-1.0
},
"lower_bound":-1000.0,
"upper_bound":1000.0,
"gene_reaction_rule":""
},
{
"id":"EX_Pi",
"name":"",
"metabolites":{
"Pi_e":-1.0
},
"lower_bound":-1000.0,
"upper_bound":1000.0,
"gene_reaction_rule":""
},
{
"id":"EX_SO4",
"name":"",
"metabolites":{
"SO4_e":-1.0
},

"lower_bound":-1000.0,
"upper_bound":1000.0,
"gene_reaction_rule":""
},
{
"id":"EX_H2O2",
"name":"",
"metabolites":{
"H2O2_e":-1.0
},
"lower_bound":-1000.0,
"upper_bound":1000.0,
"gene_reaction_rule":""
},
{
"id":"EX_O2",
"name":"",
"metabolites":{
"O2_e":-1.0
},
"lower_bound":-1000.0,
"upper_bound":1000.0,
"gene_reaction_rule":""
},
{
"id":"EX_CO2",
"name":"",
"metabolites":{
"CO2_e":-1.0
},
"lower_bound":-1000.0,
"upper_bound":1000.0,
"gene_reaction_rule":""
},
{
"id":"EX_GLUC",
"name":"",
"metabolites":{
"GLUC_e":-1.0
},
"lower_bound":-1.0,
"upper_bound":0.0,
"gene_reaction_rule":""
},
{
"id":"EX_GOH",
"name":"",
"metabolites":{
"GOH_e":-1.0
},
"lower_bound":0.0,
"upper_bound":0.0,
"gene_reaction_rule":""
},

```
{
"id":"EX_ACT",
"name":"",
"metabolites":{
"ACT_e":-1.0
},
"lower_bound":0.0,
"upper_bound":0.0,
"gene_reaction_rule":""
},
{
"id":"EX_ETOH",
"name":"",
"metabolites":{
"ETOH_e":-1.0
},
"lower_bound":0.0,
"upper_bound":1000.0,
"gene_reaction_rule":""
},
{
"id":"EX_biom",
"name":"",
"metabolites":{
"biom_e":-1.0
},
"lower_bound":0.0,
"upper_bound":1000.0,
"gene_reaction_rule":"",
"objective_coefficient":1.0
},
{
"id":"cMaintenance",
"name":"",
"metabolites":{
"ADP_c":1.0,
"ATP_c":-1.0,
"H2O_c":-1.0,
"H_c":1.0,
"Pi_c":1.0
},
"lower_bound":194.96583861363,
"upper_bound":194.96583861363,
"gene_reaction_rule":""
}
],
"genes":[],
"id":"S_cerevisiae",
"compartments":{
"c":""
},
"version":"1"
}
```
