# Supplementary material for: Mitochondrial localization of key NAD+-dependent dehydrogenases explains the exceptionally high biomass yield on ethanol of the yeast Cyberlindnera jadinii
Source: Appl Environ Microbiol. 2026 Jul 10;92(8):e00362-26. doi: 10.1128/aem.00362-26 (PMC13488300; doi:10.1128/aem.00362-26)
Supplement: Supplementary file 3 [file aem.00362-26-s0001.docx]

**SUPPLEMENTAL MATERIAL 1**

accompanying

**An exceptionally high biomass yield on ethanol of the yeast *Cyberlindnera jadinii* correlates with a mitochondrial localization of key dehydrogenases**

Marieke Warmerdam^1^, Yared O. Pijman^1^, Marc P. Pedersen^1^, Koen Wattel^1^, Marcel A. Vieira-Lara^1^, Jack T. Pronk^1*^

^1^Delft University of Technology, Department of Biotechnology, Delft, the Netherlands

Running title: Mitochondrial localization of dehydrogenases in *C. jadinii*

Address correspondence to Marcel Vieira-Lara ([m.a.vieiralara@tudelft.nl](mailto:m.a.vieiralara@tudelft.nl)) and Jack Pronk ([j.t.pronk@tudelft.nl](mailto:j.t.pronk@tudelft.nl))

Manuscript for publication in Applied and Environmental Microbiology

**Contents:**

Tables A1-A15

Figures A1-A2

**Supporting information to the use of the yeast core stoichiometric metabolic model**

Simulations of biomass formation were performed with a yeast core stoichiometric metabolic model (modified from Warmerdam et al. (2024), which was based on the model initially reported by Daran-Lapujade et al. (2004)). Previous versions of the model had two degrees of freedom (df). In this study, by adding parallel reactions in different subcellular compartments and corresponding metabolites and transport reactions between the compartments (Table A1), the number of degrees of freedom was increased (169 reactions x 165 compounds, 13 df). The matrix could consequently no longer be solved as a reduced row echelon form (rref function in MATLAB 2021 used in Warmerdam et al. (2024)). Instead, the linprog function in Python (script provided as ipynb file, Supplemental Material 3, <https://github.com/mwarmerdam/localization-code>) was used to solve the constrained set of linear equations and optimize for ethanol usage, with a fixed (i.e., constrained) amount of 100 mCmol biomass formed. The stoichiometric matrix and the sets of constraints used are given in Supplemental Material 2. A few remarks about the sets of constraints:

- Only a single biomass forming reaction was unbounded (-1000,1000), the ones corresponding to the other two species not under investigation that simulation were fully constrained (0,0) (Table A2).
- All acetaldehyde dehydrogenase reactions were constrained to only be able to run in the NAD(P)H-forming direction (0,1000).
- All three NADH dehydrogenase (i.e. types Nde, Ndi, Complex I) and quinol dehydrogenase were constrained to only run in the oxidative direction.
- Cytosolic malate dehydrogenase (cMAL deh) was constrained to only run in the NADH-forming reaction (0,1000).
- Alcohol dehydrogenase was fully constrained (0,0) to assess the theoretical maximum impact on biomass yield of fully diverting the ethanol oxidation flux through alcohol oxidase (AOX, Bounds M in Supplemental information 2).
- Maintenance was set to 195 mmol_ATP_∙Cmol_x_∙h^-1^ to match the experimental yield of 0.6 g_biomass_∙g_ethanol_^-1^ for *S. cerevisiae* (Verduyn et al., 1991, Table 1). This step was used to fit the model to empirical data and kept the same in all scenarios (all species).

The model with constraints corresponding to ‘Bounds A’ in Supplemental Material 2 (i.e.
*S. cerevisiae* with cytosolic ADH and ALD) has been expanded to include reactions involved in glucose metabolism and has been converted into a .json file compatible with cobra and is available as Supplemental Material 4 (<https://github.com/mwarmerdam/localization-code>).

**Table A1.** Compartmentalised reactions added to the yeast core stoichiometric model described in Warmerdam et al. (2024).

| Full name | Short name | Reaction |
| --- | --- | --- |
| Cytosolic alcohol oxidase | cAOX | 1 ETOH:cyt + 1 O2:cyt ⬄ 1 ACTAL:cyt + 1 H2O2:cyt |
| Mitochondrial alcohol dehydrogenase | mETOH deh | 1 ACTAL:mit + 1 H:mit + NADH:mit ⬄ 1 ETOH:mit + 1 NAD:mit |
| Mitochondrial NADH dehydrogenase, type Complex I | mNADH deh CI | 11 H:mit + 1 NADH:mit + 0,5 O2:mit => 10 H:cyt + 1 H2O:mit + 1 NAD:mit |
| Mitochondrial NADH dehydrogenase, type Ndi | mNADH deh ND | 7 H:mit + 1 NADH:mit + 0,5 O2:mit => 6 H:cyt + 1 H2O:mit + 1 NAD:mit |
| Mitochondrial NAD^+^-dependent aldehyde dehydrogenase | mACTAL deh (NAD) | 1 ACTAL:mit + H2O:mit + 1 NAD:mit ⬄ 1 ACT:mit + 2 H:mit + 1 NADH:mit |
| Mitochondrial NADP^+^-dependent aldehyde dehydrogenase | mACTAL deh (NADP) | 1 ACTAL:mit + H2O:mit + 1 NADP:mit ⬄ 1 ACT:mit + 2 H:mit + 1 NADPH:mit |
| Transport hydrogen peroxide cytosol ⬄ extracellular | cH2O2 -> eH2O2 | H2O2:cyt ⬄ H2O2:ext |
| Extracellular hydrogen peroxide reservoir | H2O2:ext | -1 H2O2:ext |
| Intracellular ethanol diffusion | cETOH -> mETOH | 1 EtOH:cyt ⬄ 1 EtOH:mit |
| Intracellular acetaldehyde diffusion | cACTAL -> mACTAL | 1 ACTAL:cyt ⬄ 1 ACTAL:mit |
| Mit. acetate carrier | cACT <-> mACT | 1 ACT:cyt + 1 H:cyt ⬄1 ACT:mit + 1 H:mit |
| Mit. oxaloacetate/malate carrier | OACm carrier | 1 MAL:cyt + 1 OXACT:mit ⬄ 1 MAL:mit + 1 OXACT:cyt |

**Table A2.** Biomass formation reactions coefficients, accounting for the difference in macromolecular composition as established in Warmerdam et al. (2024). The coefficients are to be substituted in the general biomass formation reaction: a CARBHYD:cyt + b DNA:cyt + c LIPID:cyt + d PROT:cyt + e RNA:cyt => 1 biom:ext. Note that the coefficients are slightly different than in the cited paper, as there the coefficients were derived by dividing the weight of a macromolecular constituent by the summed weight of the macromolecules, and here by dividing the macromolecule weight by the total dry weight.

|  | a | b | c | D | e |
| --- | --- | --- | --- | --- | --- |
| *Cyberlindnera jadinii* | 0.196 | 0.00377 | 0.0979 | 0.619 | 0.0743 |
| *Ogataea parapolymorpha* | 0.308 | 0.00382 | 0.102 | 0.549 | 0.0670 |
| *Saccharomyces cerevisiae* | 0.258 | 0.00375 | 0.130 | 0.446 | 0.0481 |

**Table A3.** Genome analysis of *Ogataea parapolymorpha* (GCF_000187245.1) for identifying alcohol dehydrogenase (ADH) genes. E values, percent positives obtained and aligned query range from significant alignments with >40% of the used query are presented. The tBLASTn algorithm with default settings from NCBI (<https://www.ncbi.nlm.nih.gov/>) was used for the following queries: ADH2 (349 aa, SGDID:S000004918), ADH3 (376 aa, SGDID:S000004688), and ADH6 (361 aa, SGDID:S000004937). Protein query sequences of *S. cerevisiae* strain CEN.PK were obtained from the Saccharomyces Genome Database (<https://www.yeastgenome.org/>). The position on the chromosome is the whole ORF, as annotated for the corresponding NCBI ID. If an NCBI identity indicated that a gene encoded a non-ADH protein, it is indicated in grey and not used in further analyses. The UniProt ID is given if it was detected in the proteome analysis of this study (Table A7, Supplemental information 5). Probability of mitochondrial localisation of the amino acid sequence corresponding with the NCBI ID was analysed using TargetP (Armenteros et al., 2019) and DeepMito (Savojardo et al., 2020). If mitochondrial localisation was deemed probable, the hit no. (first column) of the gene is displayed in bold.

|  |  | | Homology with | | |  |  |  |  |  |
| --- | --- | --- | --- | --- | --- | --- | --- | --- | --- | --- |
| Hit no. | Chromosome | Position | ADH2 | ADH3 (m) | ADH6 | NCBI ID | Annotated name | UniProt ID | TargetP | DeepMito |
| 1 | I  NC_027866.1 | 261584..262561 | 1e-06  42%, 3-315 | 1e-12  47%, 30-201 | - | XP_013937055.1 | Putative sorbitol dehydrogenase |  | 0.0001 | No |
| 2 | II  NC_027865.1 | 39080..40162 | 5e-09  52%, 19-171 | 2e-16  53%, 46-198 | 1e-05  45%, 35-221 | XP_013936353.1 | L-arabinitol 4-dehydrogenase |  | 0.0001 | No |
| 3 | III  NC_027864.1 | 1228828..1229919 | 4e-07  49%, 19-172 | 1e-09  49%, 46-198 | - | XP_013936305.1 | L-arabinitol 4-dehydrogenase |  | 0 | No |
| 4 |  | 865887..867008 | - | - | 3e-104  63%, 1-358 | XP_013936091.1 | NADP-dependent alcohol dehydrogenase 6 | W1QGQ7 | 0 | No |
| 5 | IV  NC_027863.1 | 1125146..1126195 | 2e-157  84%, 2-349 | 9e-161  85%, 17-376 | 2e-14  42%, 27-351 | XP_013935468.1 | Alcohol dehydrogenase 2 | W1QFP1 | 0.0001 | No |
| **6** | VII NC_027860.1 | 244350..245498 | 2e-153  83%, 2-349 | 2e-160  82%, 8-376 | 2e-17  43%, 25-351 | XP_013932311.1 | Alcohol dehydrogenase 2 | H9ZGN0 | 0.9989 | Yes |
| 7 |  | 6767..7840 | 4e-17  43%, 28-344 | - | 2e-110  69%, 3-356 | XP_013932176.1 | NADP-dependent alcohol dehydrogenase 6 |  | 0.0001 | No |
| 8 |  | 24361..25443 | 6e-13  41%, 28-344 | 3e-13  43%, 55-352 | 9e-101  66%, 3-358 | XP_013932184.1 | NADP-dependent alcohol dehydrogenase 6 |  | 0.0001 | No |
| 9 |  | 31034..32122 | - | - | 9e-83  62%, 12-358 | XP_013932188.1 | NADP-dependent alcohol dehydrogenase 7 |  | 0.0003 | No |
| 10 |  | 287298..288515 | - | - | 2e-07  38%, 30-351 | XP_013932338.1 | Formaldehyde dehydrogenase |  | 0.0373 | Yes |

**Table A4.** Genome analysis of *Cyberlindnera jadinii* (GCA_001245095.1) for identifying alcohol dehydrogenase (ADH) genes. E values, percent positives obtained and aligned query range from significant alignments with >40% of the used query are presented. The tBLASTn algorithm with default settings from NCBI (<https://www.ncbi.nlm.nih.gov/>) was used for the following queries: ADH2 (349 aa, SGDID:S000004918), ADH3 (376 aa, SGDID:S000004688), and ADH6 (361 aa, SGDID:S000004937). Protein query sequences of *S. cerevisiae* strain CEN.PK were obtained from the Saccharomyces Genome Database (<https://www.yeastgenome.org/>). The position on the chromosome is the whole ORF, as annotated for the corresponding NCBI ID. If an NCBI identity indicated that a gene encoded a non-ADH protein, it is indicated in grey and not used in further analyses. The UniProt ID is given if it was detected in the proteome analysis of this study (Table A8, Supplemental information 5). Probability of mitochondrial localisation of the amino acid sequence corresponding with the NCBI ID was analysed using TargetP (Armenteros et al., 2019) and DeepMito (Savojardo et al., 2020). If mitochondrial localisation was deemed probable, the hit no. (first column) of the gene is displayed in bold.

|  |  | | Homology with | | |  |  |  |  |  |
| --- | --- | --- | --- | --- | --- | --- | --- | --- | --- | --- |
| Hit no. | Chromosome | Position | ADH2 | ADH3 (m) | ADH6 | NCBI ID | Annotated name | UniProt ID | TargetP | DeepMito |
| 1 | I  CDQK01000001.1 | 194140..195186 | 9e-159  85%, 1-349 | 1e-173  88%, 26-376 | 1e-12  43%, 28-351 | CEP20295.1 | ADH3 | A0A1E4S0Q2 | 0 | No |
| **2** |  | 1780205..1781362 | 5e-140  80%, 1-349 | 3e-150  88%, 20-376 | 4e-16  50%, 28-172 | CEP20939.1 | ADH3 | A0A0H5CAQ2 | 0.9883 | Yes |
| 3 | II  CDQK01000002.1 | 961993..963147 | 9e-13  40%, 82-308 | 5e-10  41%, 60-193 | 7e-104  62%, 1-358 | CEP21606.1 | unnamed protein product |  | 0 | No |
| 4 |  | 1970927..1971943 | 8e-08  42%, 1-310 | 1e-11  57%, 19-114 | - | CEP21999.1 | ZTA1 |  | 0.0005 | No |
| **5** | III  CDQK01000003.1 | 1611377..1612486 | 8e-161  88%, 3-342 | 8e-174  85%, 1-369 | 7e-15  44%, 27-349 | CEP22729.1 | ADH3 | A0A0H5CDU4 | 0.9925 | Yes |
| **6** | IV  CDQK01000004.1 | 1022365..1023471 | 3e-151  82%, 1-349 | 5e-166  82%, 1-376 | 1e-12  43%, 1-172 | CEP23286.1 | ADH3 | A0A0H5C5M0 | 0.93 | Yes |
| 7 | V  CDQK01000005.1 | 812506..813713 | - | - | 7e-06  38%, 30-344 | BN1211_4754^a^ | FDH1 |  | 0 | No |
| **8** | VI  CDQK01000006.1 | 1079540..1080649 | 9e-169  89%, 2-349 | 4e-176  85%, 1-376 | 2e-15  43%, 27-351 | CEP24725.1 | ADH3 | A0A0H5C8Q7 | 0.988 | Yes |
| 9 |  | 1075995..1077044 | 1e-162  86%, 3-349 | 7e-176  86%, 16-376 | 2e-13  43%, 27-351 | CEP24723.1 | ADH3 | A0A0H5C909 | 0 | No |
| 10 |  | 1077364..1078407 | 3e-118  73%, 1-349 | 2e-124  74%, 29-376 | 7e-20  43%, 28-348 | CEP24724.1 | ADH3 |  | 0.001 | No |
| 11 |  | 178379..179524 | 9e-06  43%, 6-172 | 1e-14  45%, 9-198 | 2e-05  44%, 28-224 | BN1211_5170^b^ | BDH1 |  | 0.0002 | No |
| 12 |  | 914757..915944 | - | 1e-11  44%, 12-198 | - | CEP24661.1 | BDH1 |  | 0 | No |
| 13 | VII CDQK01000007.1 | 589702..590895 | - | 2e-11  45%, 33-198 | - | -^c^ |  |  | 0 | No |

^a^ An mRNA at this locus was annotated with a ‘clusters of eukaryotic orthologous groups’ (KOG) functional annotation ‘Alcohol dehydrogenase, class III’. For mitochondrial localisation predictions, the mRNA sequence was translated.

^b^ An mRNA at this locus was annotated with a ‘clusters of eukaryotic orthologous groups’ (KOG) functional annotation ‘Sorbitol dehydrogenase’. For mitochondrial localisation predictions, the mRNA sequence was translated.

^c^ This region was not annotated in genome GCA_001245095.1. For mitochondrial localisation predictions, the translated sequence was used.

**Table A5.** Genome analysis of *Ogataea parapolymorpha* (GCF_000187245.1) for identifying aldehyde dehydrogenase (ALD) genes. E values, percent positives obtained and aligned query range from significant alignments with >70% of the used query are presented. The tBLASTn algorithm with default settings from NCBI (<https://www.ncbi.nlm.nih.gov/>) was used for the following queries: ALD3 (507 aa, SGDID: S000004779), ALD4 (520 aa, SGDID: S000005901), and ALD6 (501 aa, SGDID:S000005982). Protein query sequences of *S. cerevisiae* strain S288C were obtained from the Saccharomyces Genome Database (<https://www.yeastgenome.org/>). The position on the chromosome is the whole ORF, as annotated for the corresponding NCBI ID. If an NCBI identity indicated that a gene encoded a non-ALD protein, it is indicated in grey and not used in further analyses. The UniProt ID is given if it was detected in the proteome analysis of this study (Table A9, Supplemental information 5). Probability of mitochondrial localisation of the amino acid sequence corresponding with the NCBI ID was analysed using TargetP (Armenteros et al., 2019) and DeepMito (Savojardo et al., 2020). If mitochondrial localisation was deemed probable, the hit no. (first column) of the gene is displayed in bold.

|  |  | | Homology with | | |  |  |  |  |  |
| --- | --- | --- | --- | --- | --- | --- | --- | --- | --- | --- |
| Hit no. | Chromosome | Position | ALD3 | ALD4 (m) | ALD6 | NCBI ID | Annotated name | UniProt ID | TargetP | DeepMito |
| 1 | II NC_027865.1 | 504520..506442 | 1e-40  46%, 82-501 | 9e-44  47%, 75-513 | 9e-45  47%, 43-496 | XP_013936636.1 | Putative aldehyde dehydrogenase-like protein |  | 0.0003 | No |
| **2** | III NC_027864.1 | 233715..235271 | 1e-119  60%, 4-502 | 0.0  82%, 9-519 | 0.0  74%, 4-500 | XP_013935713.1 | Potassium-activated aldehyde dehydrogenase, mitochondrial | W1QIV4 | 0.9995 | Yes |
| 3 |  | 1159573..1161069 | 2e-108  59%, 8-502 | 0.0  75%, 41-517 | 2e-171  71%, 3-500 | XP_013936265.1 | Potassium-activated aldehyde dehydrogenase, mitochondrial | W1QIH1 | 0.0003 | No |
| **4** |  | 723986..725668 | 3e-35  45%, 75-489 | 4e-32  45%, 65-519 | 3e-38  46%, 40-483 | XP_013936006.1 | Delta-1-pyrroline-5-carboxylate dehydrogenase |  | 0.6927 | Yes |
| 5 | IV NC_027863.1 | 806411..807898 | 2e-117  64%, 22-503 | 2e-124  61%, 29-517 | 2e-118  61%, 14-500 | XP_013935276.1 | putative aldehyde dehydrogenase-like protein | W1QDR7 | 0 | No |
| 6 |  | 453935..455434 | 4e-71  52%, 75-498 | 1e-79  56%, 45-513 | 4e-79  55%, 26-498 | XP_013935060.1 | Succinate-semialdehyde dehydrogenase [NADP(+)] |  | 0 | No |
| **7** |  | 252130..253617 | 2e-64  51%, 83-502 | 6e-78  54%, 43-513 | 2e-77  55%, 24-501 | XP_013934953.1 | putative succinate-semialdehyde dehydrogenase |  | 0.1459 | Yes |
| **8** | V NC_027862.1 | 1083226..1084710 | 7e-32  46%, 75-492 | 1e-33  47%, 174-497 | - | XP_013934654.1 | aldehyde dehydrogenase (NAD+) | W1QCJ4 | 0.6484 | Yes |
| 9 | VI NC_027861.1 | 675617..677113 | 1e-31  48%, 87-498 | 1e-29  51%, 178-496 | - | XP_013933481.1 | putative fatty aldehyde dehydrogenase | W1Q9A9 | 0.0039 | Yes |
| 10 | VII NC_027860.1 | 669768..671249 | 2e-151  68%, 3-502 | 2e-143  66%, 26-513 | 1e-118  61%, 13-500 | XP_013932544.1 | putative aldehyde dehydrogenase-like protein | W1Q743 | 0.0001 | No |
| 11 |  | 113114..114613 | 3e-130  64%, 3-505 | 1e-144  65%, 19-517 | 2e-123  61%, 13-500 | XP_013932240.1 | putative aldehyde dehydrogenase-like protein | W1Q8Y5 | 0.0001 | No |

**Table A6.** Genome analysis of *Cyberlindnera jadinii* (GCA_001245095.1) for identifying aldehyde dehydrogenase (ALD) genes. E values, percent positives obtained and aligned query range from significant alignments with >70% of the used query are presented. The tBLASTn algorithm with default settings from NCBI (<https://www.ncbi.nlm.nih.gov/>) was used for the following queries: ALD3 (507 aa, SGDID: S000004779), ALD4 (520 aa, SGDID: S000005901), and ALD6 (501 aa, SGDID:S000005982). Protein query sequences of *S. cerevisiae* strain S288C were obtained from the Saccharomyces Genome Database (<https://www.yeastgenome.org/>). The position on the chromosome is the whole ORF, as annotated for the corresponding NCBI ID. If an NCBI identity indicated that a gene encoded a non-ALD protein, it is indicated in grey and not used in further analyses. The UniProt ID is given if it was detected in the proteome analysis of this study (Table A10, Supplemental information 5). Probability of mitochondrial localisation of the amino acid sequence corresponding with the NCBI ID was analysed using TargetP (Armenteros et al., 2019) and DeepMito (Savojardo et al., 2020). If mitochondrial localisation was deemed probable, the hit no. (first column) of the gene is displayed in bold.

|  |  | | Homology with | | |  |  |  |  |  |
| --- | --- | --- | --- | --- | --- | --- | --- | --- | --- | --- |
| Hit no. | Chromosome | Position | ALD3 | ALD4 (m) | ALD6 | NCBI Protein ID | Annotated name | UniProt ID | TargetP | DeepMito |
| 1 | I  CDQK01000001.1 | 1693159-1694652 | 4e-120  59%, 5-503 | 0.0  74%, 1-517 | 0.0  74%, 8-500 | CEP20906.1 | ALD5 | A0A0H5BZW3 | 0 | No |
| 2 |  | 2187543-2189108 | 3e-39  45%, 23-461 | 1e-57  48%, 24-476 | 4e-50  47%, 26-459 | CEP21105.1 | ALD6 |  | 0.0035 | No |
| 3 |  | 416846-418402 | 4e-28  45%, 85-485 | 2e-28  46%, 82-513 | 5e-25  46%, 162-496 | BN1211_0209^a^ |  | A0A1E4S0F3 | 0 | No |
| **4** | II  CDQK01000002.1 | 1763343..1765034 | 1e-116  60%, 22-499 | 2e-135  64%, 30-514 | 3e-128  62%, 8-497 | CEP21920.1 | CYB2 |  | 0.9414 | Yes |
| 5 |  | 203587..205479 | 1e-39  47%, 35-501 | 8e-48  48%, 67-513 | 6e-46  49%, 49-496 | CEP21299.1 | MSC7 |  | 0.0001 | No |
| 6 | III  CDQK01000003.1 | 578090..579598 | 5e-158  70%, 18-505 | 9e-129  63%, 27-518 | 3e-119  61%, 14-500 | BN1211_2639^a^ |  |  | 0 | No |
| **7** |  | 2012889-2014448 | 1e-125  62%, 4-502 | 0.0  83%, 4-517 | 0.0  75%, 13-501 | XP_020068709.1^b^ | aldehyde dehydrogenase family protein | A0A1E4RWS1 | 0.996 | Yes |
| 8 |  | 592466..593968 | 2e-120  62%, 2-502 | 0.0  74%, 12-519 | 0.0  79%, 1-500 | CEP22317.1 | ALD5 | A0A060N5K5 | 0 | No |
| **9** | IV  CDQK01000004.1 | 1591164..1592729 | 3e-125  61%, 15-502 | 0.0  82%, 1-517 | 0.0  74%, 2-500 | CEP23521.1 | ALD5 | A0A0H5C647 | 0.9899 | Yes |
| **10** | V  CDQK01000005.1 | 1199746..1201488 | 6e-33  45%, 99-498 | 2e-35  47%, 65-519 | 5e-33  45%, 54-497 | CEP24183.1 | PUT2 |  | 0.1093 | Yes |
| 11 | VI  CDQK01000006.1 | 1147862..1149364 | 5e-133  64%, 1-502 | 2e-131  64%, 16-517 | 9e-120  62%, 12-500 | BN1211_5654^a^ | ALD2 | A0A1E4RZ19 | 0 | No |
| 12 |  | 489243..490715 | 6e-61  50%, 22-500 | 3e-74  52%, 23-513 | 1e-76  52%, 7-498 | BN1211_5320^a^ |  |  | 0.0001 | No |
| **13** |  | 174692..176278 | 3e-31  47%, 91-461 | 2e-56  49%, 2-484 | 3e-53  49%, 26-459 | CEP24365.1 | ALD6 | A0A060N6W0 | 0.9887 | Yes |

^a^ An mRNA at this locus was annotated with a ‘clusters of eukaryotic orthologous groups’ (KOG) functional annotation ‘Aldehyde dehydrogenase’. For mitochondrial localisation predictions, the mRNA sequence was translated.

^b^ This region was not annotated in genome GCA_001245095.1. BLASTp revealed the translation to be 99% identical (Val392Ile and Glu442Asp) to this NCBI Refseq from another whole genome assembly that is also linked to the UniProt ID. The translation of nucleotides 2012889-2014448 of CDQK01000003.1 was used for mitochondrial localisation predictions.

**Table A7.** Alcohol dehydrogenase proteins detected in aerobic, ethanol-limited steady-state chemostat cultures of *O. parapolymorpha* grown at a dilution rate of 0.10 h^-1^. Cytosolic or mitochondrial localization of proteins was predicted as a consensus from the outcome of the MitoProt (Claros & Vincens, 1996), TargetP (Armenteros et al., 2019), and DeepMito (Savojardo et al., 2020) algorithms. Calculations of the proteome percentage are presented in Supplemental information 5. The genomic location was established by performing a tBLASTn search using the protein sequence from UniProt (<https://www.uniprot.org/>) against the *O. parapolymorpha* genome (GCF_000187245.1) on the NCBI website using default settings (<https://www.ncbi.nlm.nih.gov/>).

| UniProt ID | MitoProt | TargetP | DeepMito | Proteome (%) | Genomic location |
| --- | --- | --- | --- | --- | --- |
| W1QFP1 | 0.0200 | 0 | No | 5.967 ± 0.006 | Chr. IV (1125146-1126192) |
| H9ZGN0 | 0.9611 | 1 | Yes | 0.107 ± 0.006 | Chr. VII (244353-245495) |
| W1QGQ7 | 0.0155 | 0 | No | 0.012 ± 0.006 | Chr. III (865890-867008) |
| **Total** | | | | 6.086 ± 0.006 | **% ADH** |
| **Cytosolic** | | | | 5.98 ± 0.01 | 98.2 ± 0.1 |
| **Mitochondrial** | | | | 0.107 ± 0.006 | 1.8 ± 0.1 |

**Table A8.** Alcohol dehydrogenase proteins detected in aerobic, ethanol-limited steady-state chemostat cultures of *C. jadinii* grown at a dilution rate of 0.10 h^-1^. Cytosolic or mitochondrial localization of proteins was predicted as a consensus from the outcome of the MitoProt (Claros & Vincens, 1996), TargetP (Armenteros et al., 2019), and DeepMito (Savojardo et al., 2020) algorithms. Calculations of the proteome percentage are presented in Supplemental information 5. The genomic location was established by performing a tBLASTn search using the protein sequence from UniProt (<https://www.uniprot.org/>) against the *C. jadinii* genome (GCA_001245095.1) on the NCBI website using default settings (<https://www.ncbi.nlm.nih.gov/>).

| UniProt ID | MitoProt | TargetP | DeepMito | Proteome (%) | Genomic location |
| --- | --- | --- | --- | --- | --- |
| A0A0H5C8Q7 | 0.9957 | 0.99 | Yes | 3.87 ± 0.05 | Chr. VI (1079540-1080646) |
| A0A0H5C909 | 0.0104 | 0 | No | 0.26 ± 0.01 | Chr. VI (1075995-1077041) |
| A0A0H5C5M0 | 0.9739 | 0.93 | Yes | 0.139 ± 0.005 | Chr. IV (1022365-1023468) |
| A0A0H5CDU4 | 0.9957 | 0.99 | Yes | 0.094 ± 0.002 | Chr. III (1611377-1612483) |
| A0A1E4S8C7 | 0.9700 | 0.39 | Yes | 0.062 ± 0.002 | Chr. I (665462-666541) |
| A0A0H5CAQ2 | 0.9578 | 0.99 | Yes | 0.011 ± 0.001 | Chr. I (1780208-1781326) |
| A0A1E4S0Q2 | 0.0089 | 0 | No | 0.004 ± 0.001 | Chr. I (194143-195186) |
| **Total** | | | | 4.45 ± 0.06 | **% ADH** |
| **Cytosolic** | | | | 0.27 ± 0.01 | 6.0 ± 0.2 |
| **Mitochondrial** | | | | 4.18 ± 0.05 | 94.0 ± 0.2 |

**Table A9.** Aldehyde dehydrogenase proteins detected in aerobic, ethanol-limited steady-state chemostat cultures of *O. parapolymorpha* grown at a dilution rate of 0.10 h^-1^. Cytosolic or mitochondrial localization of proteins was predicted as a consensus from the outcome of the MitoProt (Claros & Vincens, 1996), TargetP (Armenteros et al., 2019), and DeepMito (Savojardo et al., 2020) algorithms. Calculations of the proteome percentage are presented in Supplemental information 5. The genomic location was established by performing a tBLASTn search using the protein sequence from UniProt (<https://www.uniprot.org/>) against the *O. parapolymorpha* genome (GCF_000187245.1) on the NCBI website using default settings (<https://www.ncbi.nlm.nih.gov/>).

| UniProt ID | MitoProt | TargetP | DeepMito | Proteome (%) | Genomic location |
| --- | --- | --- | --- | --- | --- |
| W1QIV4 | 0.9978 | 1 | Yes | 2.69 ± 0.05 | Chr. III (233715-235268) |
| W1QIH1 | 0.2519 | 0 | No | 0.30 ± 0.02 | Chr. III (1159576-1161069) |
| W1Q743 | 0.0078 | 0 | No | 0.096 ± 0.002 | Chr. VII (669768-671246) |
| W1Q8Y5 | 0.1743 | 0 | No | 0.046 ± 0.001 | Chr. VII (113117-114613) |
| W1QDR7 | 0.0043 | 0 | No | 0.008 ± 0.001 | Chr. IV (806411-807895) |
| W1QCJ4 | 0.9849 | 0.65 | Yes | 0.007 ± 0.001 | Chr. V (1083226-1084707) |
| W1Q9A9 | 0.0272 | 0 | Yes | 0.007 ± 0.002 | Chr. VI (675617-677110) |
| **Total** | | | | 3.15 ± 0.06 | **% ALD** |
| **Cytosolic** | | | | 0.45 ± 0.02 | 14.4 ± 0.2 |
| **Mitochondrial** | | | | 2.70 ± 0.05 | 85.6 ± 0.2 |

**Table A10.** Aldehyde dehydrogenase proteins detected in aerobic, ethanol-limited stead-state chemostat cultures of *C. jadinii* grown at a dilution rate of 0.10 h^-1^. Cytosolic or mitochondrial localization of proteins was predicted as a consensus from the outcome of the MitoProt (Claros & Vincens, 1996), TargetP (Armenteros et al., 2019), and DeepMito (Savojardo et al., 2020) algorithms. Calculations of the proteome percentage are presented in Supplemental information 5. The genomic location was established by performing a tBLASTn search using the protein sequence from UniProt (<https://www.uniprot.org/>) against the *C. jadinii* genome (GCA_001245095.1) on the NCBI website using default settings (<https://www.ncbi.nlm.nih.gov/>).

| UniProt ID | MitoProt | TargetP | DeepMito | Proteome (%) | Genomic location |
| --- | --- | --- | --- | --- | --- |
| A0A060N5K5 | 0.2752 | 0 | No | 1.87 ± 0.01 | Chr. III (592466-593965) |
| A0A0H5C647 | 0.9921 | 0.99 | Yes | 0.64 ± 0.02 | Chr. IV (1591167-1592729) |
| A0A1E4RWS1 | 0.9783 | 1 | Yes | 0.05 ± 0.01 | Chr. III (2012889-2014445) |
| A0A1E4RZ19 | 0.1836 | 0 | No | 0.021 ± 0.002 | Chr. VI (1147862-1149361) |
| A0A0H5BZW3 | 0.0431 | 0 | No | 0.017 ± 0.001 | Chr. I (1693162-1694652) |
| A0A1E4S0F3 | 0.2005 | 0 | No | 0.017 ± 0.001 | Chr. I (416849-418402) |
| A0A060N6W0 | 0.9953 | 0.99 | Yes | 0.014 ± 0.005 | Chr. VI (174692-176275) |
| **Total** | | | | 2.63 ± 0.02 | **% ALD** |
| **Cytosolic** | | | | 1.93 ± 0.01 | 73.2 ± 0.2 |
| **Mitochondrial** | | | | 0.70 ± 0.01 | 26.8 ± 0.2 |

**Table A11.** Proteins detected in aerobic, ethanol-limited steady-state chemostat cultures of
*O. parapolymorpha* grown at a dilution rate of 0.10 h^-1^ with annotations related to respiratory NADH oxidation in mitochondria, either as subunits of Complex I or as a type II-NADH dehydrogenase (Supplemental information 5).

| UniProt ID | Description |
| --- | --- |
| W1QHM3 | NADH-ubiquinone oxidoreductase subunit, mitochondrial |
| W1QA17 | NADH-ubiquinone oxidoreductase subunit, mitochondrial |
| W1QA07 | NADH-ubiquinone oxidoreductase subunit, mitochondrial |
| W1QBR2 | NADH-ubiquinone oxidoreductase |
| W1QJ28 | NADH dehydrogenase [ubiquinone] flavoprotein 1, mitochondrial |
| W1QKP9 | Uncharacterized protein |
| W1QDH0 | NADH-ubiquinone oxidoreductase subunit, mitochondrial |
| W1QJW3 | Uncharacterized protein |
| W1QIG6 | NADH dehydrogenase (Ubiquinone) 1 alpha subcomplex 5 |
| W1Q7G3 | NADH dehydrogenase [ubiquinone] iron-sulfur protein 4, mitochondrial |
| W1Q745 | NADH-ubiquinone oxidoreductase subunit, mitochondrial |
| W1QJL9 | Putative NADH-ubiquinone oxidoreductase subunit, mitochondrial |
| W1QI65 | NADH dehydrogenase [ubiquinone] 1 beta subcomplex subunit 8, mitochondrial |
| W1Q8D7 | NADH dehydrogenase [ubiquinone] 1 alpha subcomplex subunit |
| W1Q9Y0 | Uncharacterized protein |
| W1QJA2 | NADH-ubiquinone oxidoreductase subunit, mitochondrial |
| W1QDP0 | NADH dehydrogenase [ubiquinone] 1 beta subcomplex subunit 9 |
| W1Q9I8 | NADH dehydrogenase (Ubiquinone) Fe-S protein 6 |
| W1QG10 | NADH dehydrogenase [ubiquinone] 1 alpha subcomplex subunit 1 |
| W1QFI3 | NADH dehydrogenase [ubiquinone] 1 alpha subcomplex subunit 13 |
| W1QGT1 | NADH:ubiquinone reductase (non-electrogenic) |
| W1Q9U4 | NADH:ubiquinone reductase (non-electrogenic) |
| W1QKE3 | Mitochondrial external NADH dehydrogenase, a type II NAD(P)H:quinone oxidoreductase |

**Table A12.** Proteins detected in aerobic, ethanol-limited chemostat steady-state chemostat cultures of
*C. jadinii* grown at a dilution rate of 0.10 h^-1^ with annotations related to respiratory NADH oxidation in mitochondria, either as subunits of Complex I or as a type II-NADH dehydrogenase (Supplemental information 5).

| UniProt ID | Description |
| --- | --- |
| A0A0H5C6I7 | NUAM subunit of mitochondrial NADH:ubiquinone oxidoreductase |
| A0A0H5C3Q2 | NUC1 protein;NADH dehydrogenase I, D subunit |
| A0A0H5C1U7 | NADH dehydrogenase [ubiquinone] flavoprotein 1, mitochondrial;NADH dehydrogenase [ubiquinone] flavoprotein 1, mitochondrial |
| A0A1E4S0M8 | NADH-ubiquinone oxidoreductase 304 kDa subunit |
| A0A0H5C684 | NADH dehydrogenase [ubiquinone] iron-sulfur protein 4, mitochondrial;NADH dehydrogenase [ubiquinone] iron-sulfur protein 4, mitochondrial |
| A0A1E4S535 | 21 kDa subunit of NADH dehydrogenase |
| A0A0H5C6X5 | NADH-quinone oxidoreductase |
| A0A0H5C7D7 | NADH-ubiquinone oxidoreductase B14 subunit (CI-B14) |
| A0A0H5CHD4 | NADH-ubiquinone oxidoreductase 23 kDa subunit mitochondrial |
| S5TEQ4 | NADH-ubiquinone oxidoreductase chain 5 |
| A0A0H5C7Z8 | NADH dehydrogenase, alpha subcomplex, subunit 2 |
| A0A1E4S7X0 | NADH dehydrogenase [ubiquinone] 1 beta subcomplex subunit 9 |
| A0A0H5CA06 | NADH-ubiquinone oxidoreductase |
| A0A1E4RUM1 | NADH dehydrogenase [ubiquinone] 1 alpha subcomplex subunit 1 |
| A0A0H5C6A4 | NADH-ubiquinone oxidoreductase 12 kDa subunit |
| A0A1E4S3Y6 | NUHM subunit of mitochondrial NADH:ubiquinone oxidoreductase |
| A0A0H5C244 | PST2 protein;NADH-quinone oxidoreductase |
| A0A0H5C087 | NADH dehydrogenase [ubiquinone] 1 alpha subcomplex subunit |
| A0A0H5C9P4 | NADH dehydrogenase [ubiquinone] 1 alpha subcomplex subunit 13 |
| A0A0H5BZ97 | NADH dehydrogenase [ubiquinone] 1 beta subcomplex subunit 7 |
| A0A0H5BYI8 | NADH:ubiquinone oxidoreductase intermediate-associated protein 30 domain-containing protein |
| A0A0H5C1I9 | NADH:ubiquinone oxidoreductase 11.5kD subunit |
| A0A0H5CFA4 | NADH-ubiquinone oxidoreductase 9.5 kDa subunit.5kD |
| A0A1E4RZC2 | NADH-ubiquinone oxidoreductase B15 subunit |
| A0A1E4RWY6 | FAD/NAD(P)-binding domain-containing protein |
| A0A1E4RUB7 | NADH:ubiquinone reductase (non-electrogenic) |
| A0A0H5C2D7 | Uncharacterized protein;FAD/NAD(P)-binding domain-containing protein |

**Table A13.** Subcellular fractionation studies to investigate localization of enzymes in *C. jadinii* grown in ethanol-limited chemostat cultures at a dilution rate of 0.10 h^-1^. Specific activity is expressed in µmol·min^-1^·(mg protein)^-1^, total activity is expressed in µmol·min^-1^ (units, U). Recoveries in the soluble and particulate fractions of cell homogenates are expressed as a percentage of the total activity in the homogenate.

|  |  | G6PDH | ADH | NAD^+^-ALD | COX |
| --- | --- | --- | --- | --- | --- |
| Homogenate | Specific activity | 0.43 ± 0.03 | 4.2 ±0.1 | 0.07 ±0.01 | 0.04 ±0.01 |
|  | Total activity R3 | 18 ± 1 | 183 ±7 | 1.8 ±0.6 | 1.7 ± 0.6 |
|  | Total activity R4 | 21 ± 1 | 196 ± 6 | 1.9 ±0.9 | 1.6 ±0.7 |
| Soluble fraction | Specific activity | 0.44 ±0.02 | 3.6 ± 0.3 | 0.06 ± 0.02 | 0.01 ± 0.01 |
|  | Total activity R3 | 13.9 ± 0.9 | 120 ± 3 | 2.0 ± 0.4 | 0.4 ± 0.5 |
|  | Total activity R4 | 15.1 ± 0.4 | 118 ± 9 | 2.3 ± 0.8 | 0.2 ± 0.5 |
|  | Average percentage recovery | 75 ± 3 | 63 ± 4 | 118 ± 10 | 18 ± 7 |
| Particulate fraction | Specific activity | 0.018 ± 0.002 | 6.4 ± 0.8 | 0.07 ± 0.02 | 0.59 ± 0.09 |
|  | Total activity R3 | 0.078 ± 0.001 | 25 ± 3 | 0.35 ± 0.01 | 2.4 ± 0.3 |
|  | Total activity R4 | 0.08 ± 0.01 | 34 ± 2 | 0.25 ± 0.08 | 3.0 ± 0.5 |
|  | Average percentage recovery | 0.41 ± 0.02 | 15 ± 2 | 16 ± 4 | 164 ± 34 |
|  | Overall percentage recovery | 75 ± 3 | 79 ± 1 | 134 ± 5 | 182 ± 27 |

**Table A14.** Subcellular fractionation studies to investigate localization of enzymes in *O. parapolymorpha* grown in ethanol-limited chemostat cultures a dilution rate of 0.10 h^-1^. Specific activity is expressed in µmol·min^-1^·(mg protein)^-1^, total activity is expressed in µmol·min^-1^ (units, U). Recoveries in the soluble and particulate fractions of cell homogenates are expressed as a percentage of the total activity in the homogenate. n.d.: not detected.

|  |  | G6PDH | ADH | NAD^+^-ALD | COX |
| --- | --- | --- | --- | --- | --- |
| Homogenate | Specific activity | 0.13 ± 0.02 | 4.0 ± 0.9 | 0.026 ± 0.009 | 0.037 ± 0.009 |
|  | Total activity R3 | 4.9 ± 0.5 | 160 ± 14 | 0.8 ± 0.4 | 1.3 ± 0.3 |
|  | Total activity R4 | 3.62 ± 0.09 | 101 ± 6 | 0.8 ± 0.4 | 1.1 ± 0.4 |
| Soluble fraction | Specific activity | 0.13 ± 0.01 | 4 ± 1 | 0.015 ± 0.005 | n.d. |
|  | Total activity R3 | 3.69 ± 0.08 | 137 ± 15 | 0.3 ± 0.2 | n.d. |
|  | Total activity R4 | 3.0 ± 0.1 | 86.8 ± 0.1 | 0.4 ± 0.1 | n.d. |
|  | Average percentage recovery | 79 ± 4 | 85.7 ± 0.6 | 43 ± 5 | n.d. |
| Particulate fraction | Specific activity | n.d. | 0.52 ± 0.07 | 0.06 ± 0.02 | 0.22 ± 0.06 |
|  | Total activity R3 | n.d. | 1.28 ± 0.05 | 0.11 ± 0.02 | 0.7 ± 0.2 |
|  | Total activity R4 | n.d. | 1.80 ± 0.06 | 0.22 ± 0.03 | 0.54 ± 0.08 |
|  | Average percentage recovery | n.d. | 1.3 ± 0.7 | 20 ± 9 | 53 ± 8 |
|  | Overall percentage recovery | 79 ± 4 | 87 ± 1 | 63 ± 15 | 53 ± 8 |

**Figure A1.** Specific activities of key enzymes in central carbon metabolism, measured in cell extracts of ethanol-grown *S. cerevisiae* (blue, left), *C. jadinii* (green, middle) and *O. parapolymorpha* (red, right), grown on synthetic medium with ethanol in shake flasks (SF) or in ethanol-limited chemostats at a dilution rate (D) of 0.10 h^-1^. Note the difference in y axis distributions as specific activities varied considerably between enzymes. Specific activities are plotted as the mean of duplicate assays with error bars representing the standard deviation. Values are also displayed in Tabel A15. A) Alcohol dehydrogenase (ADH). B, C) NAD(P)^+^-dependent aldehyde dehydrogenase (NAD(P)^+^-ALD). ALD assays for *S. cerevisiae* were performed with a lower concentration of acetaldehyde (0.1 mM) than in assays for *C. jadinii* and *O. parapolymorpha* (7 mM), due to differences in affinity and sensitivity differences in ALD. D) Glucose-6-phosphate dehydrogenase (G6PDH). Maximum specific growth rates (µ_max_) in exponentially growing shake-flask batch cultures were obtained from Warmerdam et al., (2024). Other metabolites/enzymes: ACS: acetyl-CoA synthetase; LSC: succinyl-CoA ligase; P_i_: inorganic phosphate; F6P: fructose 6-phosphate; G6P: glucose 6-phosphate; 6PGL: 6-phosphogluconolactone. PPP: pentose phosphate pathway. Created in <https://biorender.com/>.

**Table A15.** Specific activities (µmol∙min^-1^∙(mg protein)^-1^) of key enzymes in central carbon metabolism, measured in cell extracts of *S. cerevisiae*, *C. jadinii* and *O. parapolymorpha*, grown on synthetic medium with ethanol in shake flasks (SF) or in ethanol-limited chemostats at a dilution rate of 0.10 h^-1^. Values the mean of duplicate assays (and duplicate reactors) with error bars representing the standard deviation. Values are also depicted in Figure A1.

|  | *S. cerevisiae* | | *C. jadinii* | | *O. parapolymorpha* | |
| --- | --- | --- | --- | --- | --- | --- |
| **Assay** | Batch | Chemostat | Batch | Chemostat | Batch | Chemostat |
| ADH | 3.3 ± 0.3 | 7.7 ± 0.6 | 0.9 ± 0.1 | 9 ± 1 | 0.57 ± 0.08 | 5.4 ± 0.3 |
| NAD^+^-ALD^a^ | 0.22 ± 0.03 | 0.52 ±0.08 | 0.002 ± 0.001 | 0.10 ± 0.03 | 0.08 ± 0.06 | 0.16 ± 0.04 |
| NADP^+^-ALD^a^ | 0.149 ± 0.006 | 2.6 ± 0.3 | 0.25 ± 0.03 | 0.53 ± 0.03 | 0.038 ± 0.007 | 1.3 ± 0.2 |
| G6PDH | 0.33 ± 0.02 | 0.24 ± 0.01 | 0.29 ± 0.02 | 0.38 ± 0.02 | 0.078 ± 0.009 | 0.121 ± 0.005 |

^a^ ALD assays for *S. cerevisiae* were performed with a lower concentration of acetaldehyde (0.1 mM) than in assays for *C. jadinii* and *O. parapolymorpha* (7 mM), due to differences in substrate affinity and sensitivity of ALDs from these yeast species.

**Figure A2.** Fluorescent microscopy of *C. jadinii* and *O. parapolymorpha* control cells without MitoTracker. A) Fluorescent microscopy images of *C. jadinii* cells grown in ethanol- (top panel) and acetate-limited (lower panel) chemostat cultures. B) Fluorescent microscopy images of *O. parapolymorpha* cell grown in ethanol- (top panel) and acetate-limited (lower panel) chemostat cultures. DIC: Differential Interference Contrast. Green and blue channel imaging was used to check for bleedthrough. The displayed images are representative samples of steady state, ethanol-limited duplicate reactors operated at dilution rate 0.10 h^-1^. Scale bars 5 µm.
